# Multidisciplinary expert consensus on clinical diagnosis and therapies for low back pain

**DOI:** 10.1038/s41413-026-00572-y

**Published:** 2026-08-27

**Authors:** Bo Gao, Rundong Chai, Guanyi Wang, Yanfang Song, Lei Shang, Jinghui Huang, Xu Cao, Yong Hai, Zhuojing Luo, Chinese Society for the Study of the Lumbar Spine, Chinese Association of Spine, Yong Hai, Zhongqiang Chen, Shiqing Feng, Jianyuan Jiang, Fang Li, Li Li, Zhuojing Luo, Yueming Song, Zhaomin Zheng, Jun Ao, Yunbing Chang, Bohua Chen, Deyu Chen, Jianting Chen, Liang Chen, Qixin Chen, Shaoqian Cui, Shucai Deng, Huiqiang Ding, Guiyou Feng, Yanzheng Gao, Tao Guo, Zhaoqing Guo, Qing He, Baorong He, Shisheng He, Yi Hong, Zhenghua Hong, Congren Huang, Han Jiang, Dianming Jiang, Bo Li, Chunde Li, Fangcai Li, Feng Li, Ming Li, Zhongshi Li, Yu Liang, Baoge Liu, Bo Liu, Haiying Liu, Hao Liu, Hongjian Liu, Yang Liu, Yang Liu, Yuzeng Liu, Shibao Lu, Feizhou Lv, Xuexiao Ma, Haiqing Mao, Wei Mei, Zhibin Meng, Lin Nie, Baogan Peng, Qiang Qi, Bangping Qian, Longxi Ren, Dike Ruan, Zengwu Shao, Cailiang Shen, Yong Shen, Weibin Sheng, Jiangang Shi, Changtai Sun, Zhiming Sun, Xun Tang, Ye Tian, Feng Wang, Huadong Wang, Huan Wang, Peng Wang, Zhe Wang, Zili Wang, Wenwen Wu, Yongchao Wu, Zenghui Wu, Ji Wu, Chunyang Xi, Qun Xia, Jie Xiao, Zengming Xiao, Huazi Xu, Jianguang Xu, Jianzhong Xu, Jinglong Yan, Huilin Yang, Qun Yang, Zhengxu Ye, Guoyong Yin, Qiang Zhang, Wenzhi Zhang, Zhongmin Zhang, Guangmin Zhao, Jie Zhao, Changqing Zhao, Qiang Zhou, Yue Zhou, Qingsan Zhu, Yue Zhu

**Affiliations:** 1 Department of Orthopedic Surgery, Xijing Hospital, Fourth Military Medical University, Xi’an, China; 2 Department of quality management, Xijing Hospital, Fourth Military Medical University, Xi’an, China; 3 Department of Health Statistics, School of Public Health, Fourth Military Medical University, Xi’an, China; 4 Department of Orthopaedic Surgery, The Johns Hopkins University School of Medicine, Baltimore, MD USA; 5 Department of Orthopedic Surgery, Beijing Chao-Yang Hospital, Capital Medical University, Beijing, China; 6 Department of Orthopedics, Peking University Third Hospital, Peking University, Beijing, China; 7 Department of Orthopedics, General Hospital of Tianjin Medical University, Tianjin Medical University, Tianjin, China; 8 Department of Orthopedics, Huashan Hospital, Fudan University, Shanghai, China; 9 Department of Orthopedics, Beijing Military General Hospital, Beijing, China; 10 Department of Orthopedics, First Affiliated Hospital of Chinese PLA General Hospital, Beijing, China; 11 Department of Orthopedics, Fourth Military Medical University, Xi’an, China; 12 Department of Orthopedics, West China Hospital, Sichuan University, Chengdu, China; 13 Department of Orthopedics, The First Affiliated Hospital of Sun Yat-sen University, Guangzhou, Guangdong, China; 14 Department of Orthopedics, The Affiliated Hospital of Zunyi Medical University, Zunyi, China; 15 Department of Orthopedics, Guangdong Provincial People’s Hospital, Guangzhou, Guangdong, China; 16 Department of Spinal Surgery, Affiliated Hospital of Qingdao University, Qingdao, China; 17 Department of Spine Surgery, Changzheng Hospital, Naval Medical University, Shanghai, China; 18 Department of Orthopedics, Nanfang Hospital, Southern Medical University, Guangzhou, China; 19 Department of Orthopedic Surgery, Orthopedic Institute, The First Affiliated Hospital of Soochow University, Suzhou, China; 20 The Department of Orthopedics, The Second Affiliated Hospital of Zhejiang University School of Medicine, Hangzhou, China; 21 Department of Spine Surgery, Shengjing Hospital of China Medical University, Shenyang, China; 22 Department of Spine Surgery, Tianjin Hospital, Tianjin, China; 23 Department of Spinal Orthopedics, General Hospital of Ningxia Medical University, Yinchuan, China; 24 Department of Orthopedics and Traumatology, Alice Ho Miu Ling Nethersole Hospital, Tai Po, China; 25 Department of Spine and Spinal Cord Surgery, Henan Provincial People’s Hospital, People’s Hospital of Zhengzhou University, Zhengzhou, China; 26 Department of Orthopedic Surgery, Guizhou Provincial People’s hospital, Guiyang, China; 27 Department of Orthopedic Surgery, Peking University Third Hospital & Peking University International Hospital, Beijing, China; 28 Department of Orthopedic Surgery, Naval General Hospital of Chinese People’s Liberation Army, Beijing, China; 29 Department of Orthopedic Surgery, Xi’an Honghui Hospital, Xi’an, China; 30 Department of Orthopedic Surgery, Shanghai Tenth People’s Hospital, Tongji University, Shanghai, China; 31 Department of Orthopedic Surgery, China Rehabilitation Research Center, Beijing, China; 32 Department of Orthopedic Surgery, Taizhou Hospital of Zhejiang Province, Linhai, China; 33 Department of Orthopedic Surgery, Xiamen Changgeng Hospital, Xiamen, Fujian, China; 34 Department of Orthopedic Surgery, Tianjin Third Central Hospital, Tianjin, China; 35 Department of Orthopedic Surgery, The First Affiliated Hospital of Chongqing Medical University, Chongqing, China; 36 Department of Orthopedics, Guizhou Provincial People’s Hospital, Guiyang, China; 37 Department of Orthopedic Spine Surgery, Peking University First Hospital, Beijing, China; 38 Department of Orthopedics, The Second Affiliated Hospital, Zhejiang University School of Medicine, Hangzhou, China; 39 Department of Orthopedic Surgery and Biological Engineering and Regenerative Medicine Center, Tongji Hospital, Huazhong University of Science and Technology, Wuhan, China; 40 Department of Spine Surgery, Changhai Hospital, Second Military Medical University, Shanghai, China; 41 Department of Spine Surgery, Chinese Japanese Friendship Hospital, Beijing, China; 42 Department of Orthopedics, Ruijin Hospital, Shanghai Jiao Tong University School of Medicine, Shanghai, China; 43 Department of Orthopedic Surgery, Beijing Tiantan Hospital, Capital Medical University, Beijing, China; 44 Department of Spine Surgery, Beijing Jishuitan Hospital, Capital Medical University, Beijing, China; 45 Department of Spine Surgery, Peking University People’s Hospital, Beijing, China; 46 Department of Orthopedic Surgery, West China Hospital, Sichuan University, Chengdu, China; 47 Department of Orthopedics, The First Affiliated Hospital of Zhengzhou University, Zhengzhou, China; 48 Department of Orthopedics, The First Affiliated Hospital of Dalian Medical University, Dalian, China; 49 Department of Orthopedics, Changzheng Hospital, Naval Medical University (Second Military Medical University), Shanghai, China; 50 Department of Orthopedic Surgery, Beijing Xuanwu Hospital, Capital Medical University, Beijing, China; 51 Department of Orthopedics, The Affiliated Hospital of Qingdao University, Qingdao, China; 52 Department of Orthopedic Surgery, The First Affiliated Hospital of Soochow University, Suzhou, China; 53 Department of Spine Surgery, Zhengzhou Orthopedics Hospital, Zhengzhou, China; 54 Department of Spine Surgery, The First Affiliated Hospital of Hainan Medical University, Haikou, China; 55 Department of Spine Surgery, Qilu Hospital of Shandong University, Jinan, China; 56 Department of Orthopedics, The Third Medical Center, General Hospital of the Chinese People’s Liberation Army, Beijing, China; 57 Department of Orthopedics, Peking University Third Hospital, Beijing, China; 58 Division of Spine Surgery, Nanjing Drum Tower Hospital, Affiliated Hospital of Medical School, Nanjing University, Nanjing, China; 59 Department of Orthopedics, ChuiYangLiu Hospital Affiliated to Tsinghua University, Beijing, China; 60 Department of Orthopedic Surgery, The Sixth Medical Centre of PLA General Hospital, Beijing, China; 61 Department of Orthopedics, Union Hospital, Tongji Medical College, Huazhong University of Science and Technology, Wuhan, China; 62 Department of Orthopedics and Spine Surgery, The First Affiliated Hospital, Anhui Medical University, Hefei, China; 63 Department of Spine Surgery, The Third Hospital of Hebei Medical University, Shijiazhuang, Hebei, China; 64 Department of Spinal Surgery, The First Affiliated Hospital of Xinjiang Medical University, Urumqi, China; 65 Department of Spine Surgery, Orthopedic Hospital, Shanghai Changzheng Hospital, Naval Medical University (Second Military Medical University), Shanghai, China; 66 Department of Orthopedics, Beijing Hospital, National Center of Gerontology, Institute of Geriatric Medicine, Chinese Academy of Medical Sciences, Beijing, China; 67 Department of Orthopedics, Tianjin Huanhu Hospital, Tianjin, China; 68 Department of Orthopedics, Kunming General Hospital of Chengdu Military Command, Kunming, China; 69 Department of Orthopedics, Peking Union Medical College Hospital, Peking Union Medical College, Beijing, China; 70 Department of Orthopedics, The First Hospital of China Medical University, Shenyang, China; 71 Department of Orthopedics, The First Affiliated Hospital of General Hospital of PLA, Beijing, China; 72 Department of Orthopedic Surgery, Shengjing Hospital of China Medical University, Shenyang, China; 73 Department of Orthopedic Surgery, Sun Yat-sen Memorial Hospital, Sun Yat-sen University, Guangzhou, Guangdong, China; 74 Department of Orthopedic Surgery, General Hospital of Ningxia Medical University, Yinchuan, China; 75 Department of Orthopedic Surgery, The First Affiliated Hospital of Chinese PLA General Hospital, Beijing, China; 76 Department of Orthopedic Surgery, Xiehe Hospital, Tongji Medical College, Huazhong University of Science and Technology, Wuhan, China; 77 Department of Orthopedic Surgery, Guangzhou General Hospital, Guangzhou, Guangdong, China; 78 Department of Orthopedic Surgery, Air Force General Hospital, Beijing, China; 79 Department of Orthopedic Surgery, The Second Affiliated Hospital of Harbin Medical University, Harbin, China; 80 Department of Orthopedic Surgery, Affiliated Hospital of Logistics University of Chinese People’s Armed Police Force, Tianjin, China; 81 Department of Spine Surgery, The Fourth People’s Hospital of Guiyang, Guiyang, China; 82 Department of Spine and Osteopathy Surgery, The First Affiliated Hospital of Guangxi Medical University, Nanning, China; 83 Department of Orthopedics, The Second Affiliated Hospital & Yuying Children’s Hospital of Wenzhou Medical University, Wenzhou, China; 84 Department of Orthopedic Surgery, Shanghai Sixth People’s Hospital, Shanghai Jiao Tong University, Shanghai, China; 85 Department of Orthopedics, Southwest Hospital, Army Medical University (Third Military Medical University), Chongqing, China; 86 Department of Orthopedics, The First Affiliated Hospital of Soochow University, Suzhou, China; 87 Department of Spine Surgery, First Affiliated Hospital of Dalian Medical University, Dalian, China; 88 Department of Orthopedics, The First Affiliated Hospital of Nanjing Medical University, Nanjing, China; 89 Department of Orthopedics, Beijing Ditan Hospital, Capital Medical University, Beijing, China; 90 Department of Orthopedics & Traumatology, Li Ka Shing Faculty of Medicine, The University of Hong Kong, Pokfulam, China; 91 Department of Spine Surgery, Department of Orthopedics, Nanfang Hospital, Southern Medical University, Guangzhou, China; 92 Department of Orthopedics, Southwest Hospital, Army Medical University, Chongqing, China; 93 Department of Orthopedics, Shanghai Key Laboratory of Orthopedic Implants, Ninth People’s Hospital, Shanghai Jiao Tong University School of Medicine, Shanghai, China; 94 Department of Orthopedics, Xinqiao Hospital, Army Medical University, Chongqing, China; 95 Department of Orthopedics of the China-Japan Union Hospital of Jilin University, Changchun, Jilin, China

**Keywords:** Pathogenesis, Bone

## Abstract

Low back pain (LBP) is one of the most frequently encountered conditions in orthopedics, physical therapy and rehabilitation, and pain medicine. Its etiology is multifactorial and its management options are diverse. However, a unified, standardized, and evidence-based clinical pathway for LBP remains lacking, leading to substantial variation in diagnostic reasoning, treatment selection, outcome assessment, and long-term care across healthcare settings and professional disciplines. To address this gap, Chinese Society for the Study of the Lumbar Spine, Chinese Association of Spine initiated this multidisciplinary expert consensus. The project was organized and implemented by Xijing Hospital of Fourth Military Medical University and Beijing Chaoyang Hospital of Capital Medical University, in collaboration with a national multicenter panel of clinical experts and methodologists. Using rigorous evidence-based methodology, the panel developed an expert consensus on recommendation levels for clinical diagnosis and treatment pathways in LBP. Comprehensive searches of multiple databases and guideline sources were performed from 1991 to December 2025 in this consensus. A total of 4 703 publications were initially retrieved, and 653 publications were ultimately selected to inform the development of the consensus statements, including 70 systematic reviews and 145 original studies that directly supported the formulation and grading of recommendations. Centered on key elements across the entire care continuum, including precise diagnostic assessment, stepwise treatment selection, multimodal comprehensive interventions, long-term outcome management, recurrence prevention, patient education, and self-management, this consensus provides a structured set of recommendations. It clarifies the core diagnostic criteria and intervention strategies for patients with low back pain caused by different etiologies, and classifies interventions as “strong recommendation”, “weak recommendation”, “weak recommendation against”, or “strong recommendation against”. The recommendations emphasize individualized and stepwise decision-making tailored to patient-specific circumstances, reaffirm the foundational role of conservative management, and define strict indications for surgery and other invasive interventions. In addition, the consensus evaluates the potential synergistic effects of combined treatment strategies and highlights the importance of recurrence prevention and functional rehabilitation in long-term care. It also provides guidance on multidisciplinary collaboration and referral criteria within the clinical pathway. Overall, this consensus aims to provide clinicians at all levels of healthcare with a clear and authoritative decision-making framework. By promoting a shift from experience-based and fragmented practice toward systematic, standardized, and evidence-based management, this consensus is expected to improve clinical outcomes, enhance patients’ quality of life, and support more efficient use and distribution of healthcare resources.

**Figure:**
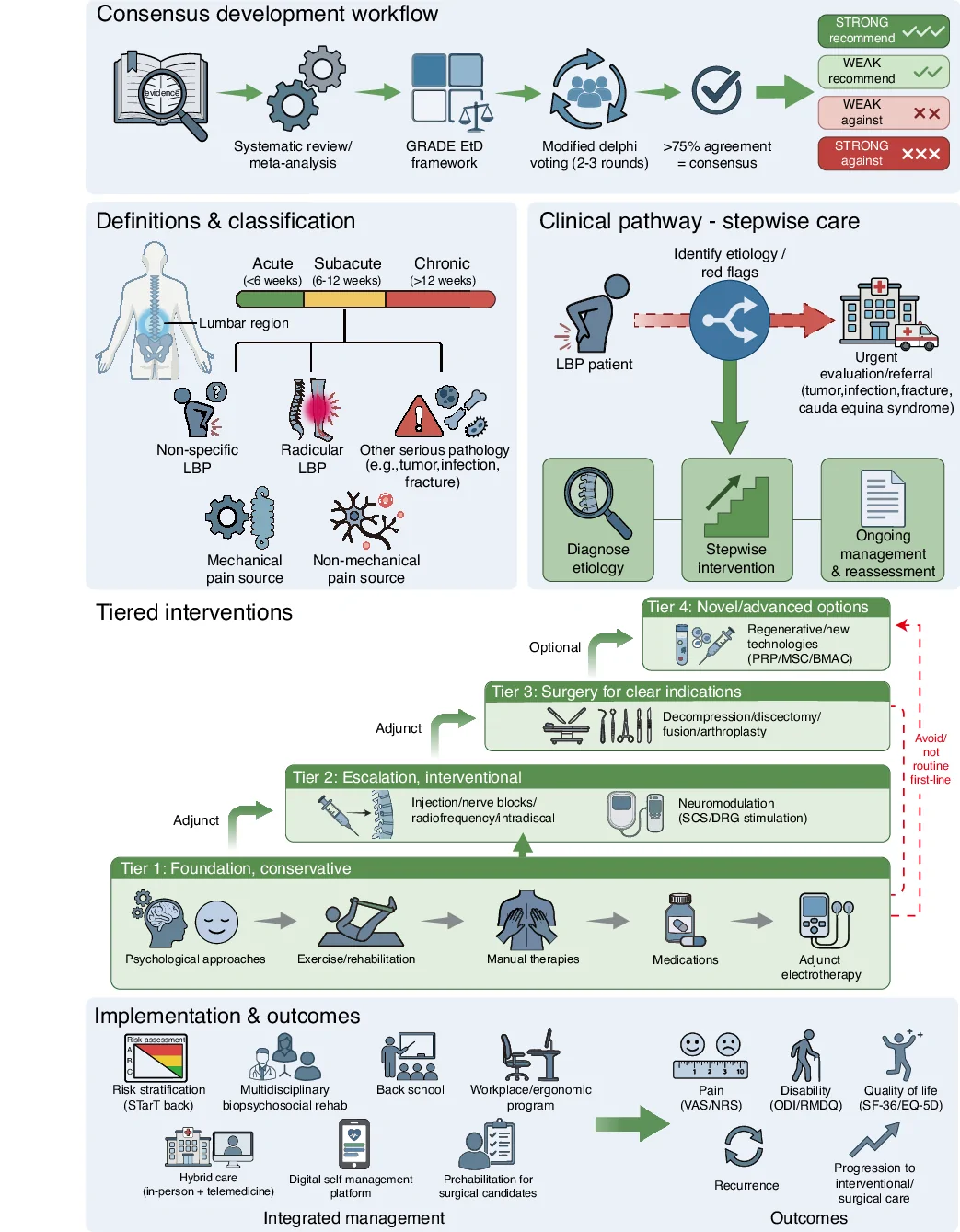

## Introduction

Low back pain (LBP) is one of the most common musculoskeletal disorders worldwide and has become a major public health problem contributing to increasing functional impairment and disability burden.^1^ Epidemiological studies indicate that approximately 90% of individuals experience at least one episode of LBP during their lifetime, and a substantial proportion may progress to chronic low back pain, profoundly reducing quality of life and imposing a significant burden on healthcare systems and socioeconomic development.^2^ Moreover, with accelerating population aging, the widespread adoption of sedentary lifestyles, and rising psychosocial stress, the prevalence and recurrence of LBP continue to increase. LBP is characterized by marked etiological heterogeneity, and its onset and progression involve multiple levels, including intervertebral disc degeneration, facet joint dysfunction, ligament and muscle functional impairment, neural sensitization, and psychosocial factors, resulting in highly heterogeneous clinical manifestations.^3^ Imaging studies show that degenerative lumbar changes are highly prevalent in middle-aged and older adults; however, imaging abnormalities do not fully correspond to clinical symptoms, further highlighting the complexity of LBP in both pathophysiological mechanisms and clinical presentation. Consequently, this etiological complexity makes it difficult for any single discipline or single therapeutic modality to comprehensively address the diagnostic and management needs of LBP, underscoring the importance of a multidisciplinary model of care.

In recent years, the clinical management paradigm for LBP has gradually shifted from single-modality approaches toward a comprehensive biopsychosocial model. Current evidence indicates that multiple treatment modalities may play important roles in different subgroups of patients with LBP. These include exercise and rehabilitation training, psychological and behavioral interventions, pharmacotherapy, interventional procedures, surgery, and emerging digital health and neuromodulation technologies.^4^ However, in current clinical practice, substantial variability remains across treatment modalities with respect to indication selection, care pathways, dosing parameters, and outcome evaluation, reflecting the absence of unified, systematic, and operational guidance for practice. Although multiple guidelines and expert consensuses related to LBP have been published nationally and internationally, most focus on a single discipline or specific intervention and have not yet evolved into an integrated consensus document that spans multiple disciplines and modalities while balancing evidence-based medicine with real-world clinical practice.^5,6^ This gap between evidence and practice creates considerable uncertainty for clinicians in selecting treatment strategies, determining intervention parameters, and implementing stratified management, thereby limiting the standardized application of LBP care. In addition, terminology and concepts are used inconsistently across the LBP literature. For example, when describing exercise interventions, psychological therapies, or integrated management strategies, operational definitions, intervention dosage, and implementation pathways vary widely. Such inconsistencies in terminology and methodology fragment the evidence base and reduce comparability across studies. Therefore, it is necessary to systematically appraise and synthesize existing evidence and, on the basis of harmonized concepts and a standardized methodological framework, develop consensus-based guidance for clinical practice. By incorporating the most recent evidence and management concepts, this updated consensus emphasizes patient-centered lifestyle management and multidisciplinary, multimodal interventions, with the aim of improving the scientific rigor, standardization, and practical applicability of LBP care in clinical settings.

In summary, considering the continuously evolving evidence base in low back pain research and the growing needs of clinical practice, it is both important and urgent to develop the multidisciplinary expert consensus on clinical diagnosis and therapies for low back pain. This consensus was developed in strict accordance with evidence-based methodology, including comprehensive literature searches, appraisal of evidence quality, and systematic synthesis of findings, based on which a draft was produced. During the consensus development process, opinions from experts across multiple disciplines—including orthopedics, sports medicine, rehabilitation medicine, pain medicine, nutritional medicine, psychosomatic/psychological medicine, and geriatrics—were extensively solicited and integrated. Through multiple rounds of group discussion and iterative revisions, consensus was ultimately achieved for each recommendation based on the available clinical evidence. This document systematically summarizes the major etiological categories of low back pain and their corresponding diagnostic and therapeutic strategies, clarifies the applicability and recommendation level of different treatment modalities across different patient populations, and aims to provide clinicians with scientific, standardized, and operational decision support. Comprehensive searches of multiple databases and guideline sources were performed from 1991 to December 2025 in this consensus. A total of 4 703 publications were initially retrieved, and 653 publications were ultimately selected to inform the development of the consensus statements, including 70 systematic reviews and 145 original studies that directly supported the formulation and grading of recommendations. By synthesizing evidence across different study designs, the consensus seeks to strike a reasonable balance between certainty of evidence and clinical feasibility, and to deliver evidence-based, comprehensive recommendations with the capacity for sustainable updates. With the development and dissemination of this multidisciplinary expert consensus, it is expected not only to provide a more unified and standardized basis for the clinical management of low back pain, but also to improve overall quality of care, facilitate multidisciplinary collaboration and the effective implementation of individualized treatment strategies, and lay a foundation for future research and continuous optimization of clinical practice—ultimately improving long-term health outcomes for patients with low back pain and reducing the societal burden of the disease.

## Materials And Methods

**Fig. 1:**
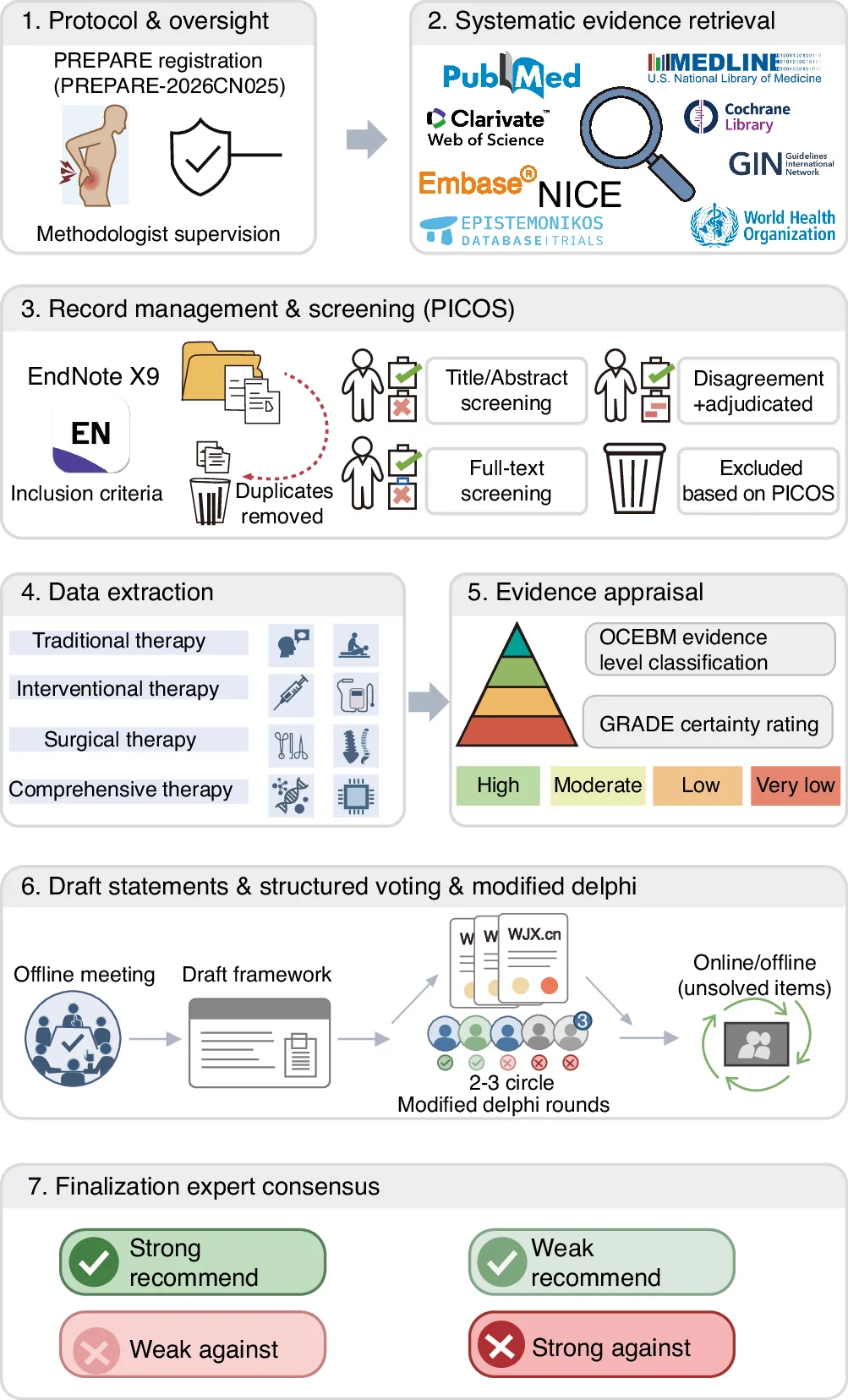
Workflow for developing the multidisciplinary expert consensus using a modified Delphi approach. The process consisted of seven sequential steps: (1) protocol registration (PREPARE-2026CN025) and continuous methodologist oversight; (2) systematic evidence retrieval from multiple databases and guideline sources up to December 2025; (3) record management, de-duplication, and screening based on PICOS using reference-management software and structured eligibility criteria; (4) data extraction and categorization of interventions into predefined clusters; (5) evidence appraisal using evidence-level classification and GRADE certainty ratings; (6) drafting recommendation statements followed by structured voting through 2–3 rounds of anonymous modified Delphi surveys (wjx.cn) with feedback and revision, and an online face-to-face consensus meeting for unresolved items; and (7) finalization of graded recommendations, categorized as strong/weak recommendations for or against an intervention

This consensus was developed using a modified Delphi approach. The process incorporated a systematic review of the literature and integration of clinical expert opinions, and the recommendations were discussed and finalized through two rounds of anonymous questionnaire surveys (www.wjx.cn) followed by a third round of online, face-to-face consensus meetings as shown in Fig. 1 (September 2025 to January 2026). A methodology expert reviewed the development methods and provided continuous oversight throughout the process. The study protocol was registered with the platform for the registration of practice guidelines in healthcare (No.PREPARE-2026CN025) and can be accessed via (http://guidelines-registry.org/). The first step in developing the consensus was to systematically collate and summarize existing evidence related to low back pain treatments. The research team searched multiple bibliographic databases and completed evidence synthesis and grading. On this basis, experts from relevant disciplines were invited to form a consensus panel, which evaluated each proposed recommendation in terms of recommendation strength and the level of agreement. After multiple rounds of anonymous scoring, feedback, and discussion, the final set of recommendations and the consensus document were established and confirmed.

### Initiation of the consensus and supporting organizations

This consensus was initiated by Chinese Society for the Study of the Lumbar Spine, Chinese Association of Spine. It was organized, implemented, and developed by Xijing Hospital of Fourth Military Medical University and Beijing Chaoyang Hospital of Capital Medical University, with methodological support provided by the GRADE China Center/Lanzhou University Evidence-Based Medicine Center (Grade of Recommendation Assessment, Development and Evaluation, GRADE).

### Information retrieval and sources

This consensus was developed on the basis of evidence from systematic reviews and meta-analyses, with additional inclusion of primary studies when high-quality review evidence was unavailable. The databases searched included PubMed, Embase, Medline, the Cochrane Library, Web of Science, and Epistemonikos. In addition, commonly used international clinical guideline websites were systematically searched, including the U.S. National Guideline Clearinghouse (NGC), the Guidelines International Network (GIN), the Scottish Intercollegiate Guidelines Network (SIGN), the National Institute for Health and Care Excellence (NICE), and the official website of the World Health Organization (WHO). Google Scholar, UpToDate, and DynaMed were also searched to identify primary research evidence, including randomized controlled trials, cohort studies, case–control studies, case series, and cross-sectional studies. The search period covered database inception through December 2025, and the language was restricted to English. Reference lists of all included articles were also hand-searched to identify potentially missed studies. Search terms focused on low back pain and its treatment and included, but were not limited to “Low Back Pain”, “Therapeutics”, “Therapy”, “Treatment”, “Lumbago”, “Low Back Ache”, “Mechanical”, “Posterior Compartment”, “Posture”, “Recurrent”, “Low Backache”, “Lower Back Pain”, “Mechanical Low Back Pain”, “Postural Low Back Pain” and “Recurrent Low Back Pain” combined with corresponding Chinese search terms where applicable. The detailed search strategy and study selection flow will be reported in the Supplementary Materials. Study screening, data extraction, and quality assessment were independently performed by at least two reviewers. Discrepancies were resolved by discussion and consensus and adjudicated by a third reviewer when necessary. Evidence quality appraisal and determination of recommendation strength were conducted with reference to the 2009 Oxford Centre for Evidence-Based Medicine (OCEBM) Levels of Evidence, and the certainty of evidence for key outcomes was additionally graded using the GRADE (Grading of Recommendations Assessment, Development and Evaluation) approach (high, moderate, low, or very low), in conjunction with the results of systematic reviews. When multiple sources of evidence supported the same recommendation, the panel comprehensively considered study design and risk of bias, inconsistency, indirectness, imprecision, and publication bias, prioritizing the highest level and certainty of evidence as the primary basis. When appropriate, evidence from different sources was integrated to determine the final strength of recommendation.

### Screening process

Under the guidance of a methodology expert, the development of this consensus strictly followed the PICOS framework—Population, Intervention, Comparison, Outcome, and Study design. Inclusion and exclusion criteria were prespecified, and a pilot screening was conducted before formal screening to harmonize operational definitions and decision rules, thereby ensuring consistency and reproducibility. First, all retrieved records were imported into EndNote X9 (Clarivate Analytics, Philadelphia, PA, USA) and managed by two reviewers. De-duplication was performed using a combination of automated detection and manual verification to prevent the same study from being counted more than once due to overlap across databases. After de-duplication, a unified screening library was established in EndNote X9 to facilitate collaborative management and to support annotation, grouping, and documentation/tracking of screening decisions. Study selection was performed in a stepwise manner:

- Title and abstract screening: Two reviewers independently screened titles and abstracts to assess eligibility based on the prespecified criteria and documented reasons for exclusion (e.g., ineligible population, non-target intervention, inappropriate study design, or non-relevant outcomes).
- Full-text screening: Full texts were obtained for records deemed potentially eligible or for which eligibility could not be determined from the abstract. The same two reviewers independently assessed full texts to determine final inclusion. Disagreements at any stage were resolved through discussion; if consensus could not be reached, a third reviewer adjudicated. To further enhance consistency and reproducibility, the research team conducted a pilot screening on a subset of sample records prior to formal screening to refine the boundaries of the inclusion/exclusion criteria and improve operational procedures.

When multiple publications originated from the same study, such as conference abstracts and full articles, or reports with different follow-up time points, the records were merged and treated as a single study after cross-checking authorship, population, trial registration information, and sample characteristics. This was done to avoid duplicate inclusion. When different reports provided complementary information, we prioritized the version with the most complete data, the most detailed methodological description, and the follow-up duration most consistent with the prespecified analysis window. Other reports were retained for supplementary data extraction.

### Eligibility criteria

The specific inclusion and exclusion criteria for references considered in this consensus were as follows:

- Population: The target population primarily included individuals diagnosed with low back pain–related conditions, including but not limited to: chronic non-specific low back pain (CNLBP)/chronic low back pain (CLBP); low back pain with or without sciatica/radicular pain; discogenic low back pain; lumbar disc herniation (LDH); degenerative-related low back pain such as lumbar spinal stenosis; facet-mediated pain/facet osteoarthritis (facet OA); sacroiliac joint–mediated pain; myofascial low back pain; low back pain associated with clinical spinal instability; and inflammatory low back pain/chronic low back pain related to ankylosing spondylitis.
  The main excluded populations were those in whom low back pain was primarily due to serious specific causes (“red flags”), such as tumor, infection, acute fracture, cauda equina syndrome, etc.; acute postoperative pain or short-term perioperative pain; conditions where low back pain was not the primary complaint (e.g., isolated neck or thoracic pain); or studies in which low back pain subtypes and/or key outcome data could not be clearly distinguished.
- Age range: All age groups were eligible.
- Interventions: Eligible interventions included, but were not limited to:

Conservative treatments: cognitive functional therapy (CFT); pain neuroscience education (PNE); acceptance and commitment therapy–informed physiotherapy (PACT); motivational interviewing (MI); mindfulness meditation; self-compassion interventions; clinical hypnosis; core stabilization and motor control training; McKenzie method; sling exercise; proprioceptive neuromuscular facilitation (PNF); blood flow restriction (BFR) training; aquatic exercise; Pilates; yoga; Tai Chi/Baduanjin/Qigong; Vojta therapy; Feldenkrais method; myofascial release; connective tissue massage; Tuina; spinal manipulation; osteopathic/visceral manipulation; instrument-assisted soft tissue mobilization (IASTM); transcutaneous electrical nerve stimulation (TENS); non-steroidal anti-inflammatory drugs (NSAIDs); acetaminophen; muscle relaxants; opioids; serotonin–norepinephrine reuptake inhibitors (SNRIs); and biologic agents.

Interventional treatments: epidural or nerve root injections; sacroiliac joint injections; trigger-point injections; facet medial branch blocks and/or radiofrequency ablation; intradiscal therapies (e.g., IDET, radiofrequency, ozone therapy); pulsed radiofrequency (PRF); spinal cord stimulation (SCS); dorsal root ganglion (DRG) stimulation; peripheral nerve field stimulation (PNFS); intradiscal platelet-rich plasma (PRP) injection; intradiscal mesenchymal stem cells; and bone marrow aspirate concentrate (BMAC).

Surgical treatments: endoscopic discectomy; spinal canal decompression; spinal fusion; artificial disc replacement; and sacroiliac joint fusion, among others.

Comprehensive/interdisciplinary management models: risk-stratified care (e.g., STarT Back); multidisciplinary biopsychosocial rehabilitation; back school programs; workplace/ergonomic interventions; hybrid care (in-person plus telehealth); digital self-management platforms; and prehabilitation prior to surgery.

(d) Comparators: Comparators included but were not limited to: placebo/sham interventions; no treatment/wait-list control; usual care; and other active comparators, such as conventional physiotherapy, usual exercise, medication comparators, different manual techniques, different dosing or frequency regimens, and comparisons with standard face-to-face physiotherapy or usual pathway management.

(e) Outcomes: Based on the existing literature, no outcomes were excluded in order to comprehensively capture study findings; however, included studies were required to report at least one of the following outcomes to support the development of this consensus: VAS, NRS/NPRS, ODI, RMDQ, SF-36, EQ-5D, etc. Secondary outcomes could include kinesiophobia (TSK), catastrophizing, self-efficacy (PSEQ), physical activity (MVPA), range of motion (ROM), muscle endurance/physical capacity, surface electromyography, recurrence rate, and clinical trajectories (e.g., progression to interventional or surgical care).

(f) Study design: Included: systematic reviews/meta-analyses; randomized controlled trials (RCTs); non-randomized controlled studies; prospective cohort/observational studies (when RCT evidence was insufficient, particularly for interventional/surgical/new technology items); registered RCT protocols and mixed-methods protocols could be included in a “research recommendations” section but were not used as direct evidence for strong clinical recommendations.

Excluded: narrative reviews, commentaries, opinion pieces; conference abstracts only when full data were unavailable; case reports and very small case series (a threshold could be applied, e.g., *n* < 10 or studies unable to provide outcome data).

### Data extraction and consistency control

**Fig. 2:**
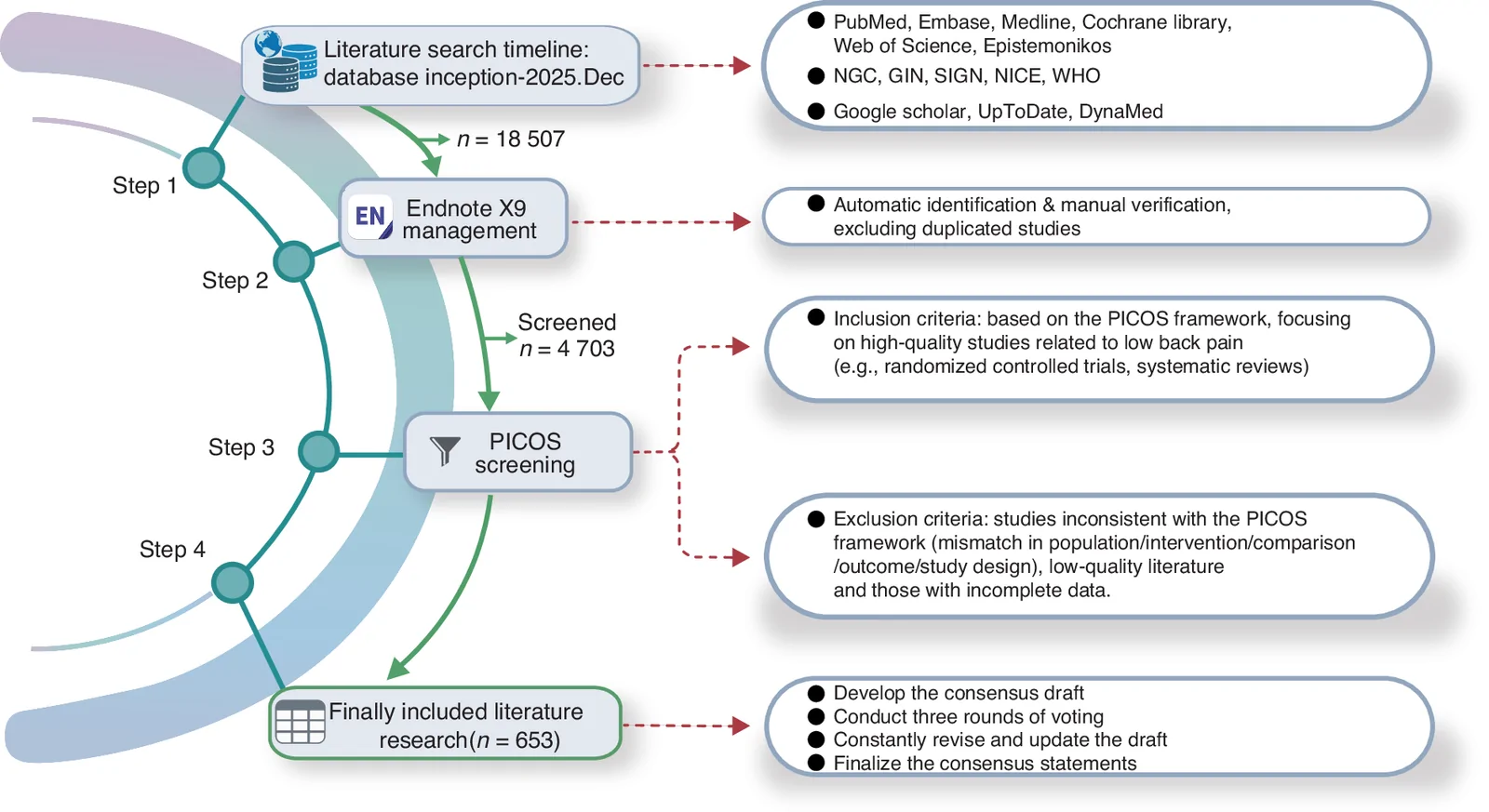
Literature search and study selection flow for evidence synthesis supporting the consensus. Records were retrieved from major bibliographic databases (PubMed, Embase, Medline, Web of Science, Epistemonikos), guideline repositories (NGC, GIN, SIGN, NICE, WHO), and supplementary sources (Google Scholar, UpToDate, DynaMed) from database inception to December 2025. All citations were imported into EndNote X9 for record management and de-duplication using automated identification with manual verification. Studies were then screened according to the PICOS framework, applying prespecified inclusion and exclusion criteria. Of 18 507 records identified, 4 703 were screened, and 653 studies were ultimately included to inform drafting, iterative voting rounds, and finalization of the consensus statements

After screening, all studies finally included proceeded to the data extraction stage. Using a prespecified standardized data extraction form, two reviewers independently extracted and cross-checked key information, including: basic study characteristics (authors, year, country/region, study design); participant characteristics (sample size, age, symptom duration, diagnostic criteria and/or stratification features); details of the intervention and comparator (type, dose/frequency, treatment duration, and delivery/implementation); follow-up time points; primary and secondary outcomes and measurement instruments; effect-size data and statistical indices (primarily means/standard deviations, risk ratios, confidence intervals, etc.); as well as adverse events and dropout/withdrawal information. If inconsistencies or missing information were identified during extraction, the two reviewers first discussed and verified the original text. When necessary, a third reviewer was consulted, or the corresponding authors were contacted to obtain critical missing data. The entire screening and extraction process followed principles of transparency and traceability. The number of records at each stage, reasons for exclusion, and the final included studies are presented in Fig. 2 to enable readers to understand the study selection pathway and evidence sources. In total, 4 703 publications were initially retrieved, and—following agreement by the expert panel—653 publications were ultimately selected to draft and support the consensus statements.

### Assessment of methodological quality and recommendations of evidence

In this study, the certainty of evidence for key outcomes was evaluated using the GRADE approach, and evidence quality was classified into four levels—high, moderate, low, and very low (as shown in Table 1). During evidence grading, judgments were made in accordance with predefined downgrading and upgrading rules. The GRADE assessments were conducted by the consensus development group under the supervision of methodologists, and all assessment results are shown in Table 2.

**Table 1:** Classification of evidence quality

GRADE	GRADE Criteria
⨁⨁⨁⨁	High-quality randomized controlled trials (RCTs); observational studies upgraded by two levels
⨁⨁⨁○	RCTs downgraded by one level; observational studies upgraded by one level
⨁⨁○○	RCTs downgraded by two levels; observational studies (no upgrading)
⨁○○○	RCTs downgraded by three levels; observational studies downgraded by one level; case series; case reports

⨁○○○: very low level of evidence, ⨁⨁○○: low level of evidence, ⨁⨁⨁○: moderate level of evidence, ⨁⨁⨁⨁: high level of evidence

**Table 2:** Evidence Level of Oxford Centre for Evidence-Based Medicine (OCEBM)

Levels of Evidence	Assessment Criteria
I	Systematic review or meta-analysis of randomized controlled trials (RCTs); high-quality prospective studies; high-quality RCTs
II	Systematic review or meta-analysis of cohort studies; lower-quality prospective studies or RCTs (e.g., inadequate randomization, lack of blinding, follow-up rate <80%)
III	Case–control studies or retrospective studies
IV	Single case-series studies
V	Expert opinion

The following four downgrading domains were primarily considered:

- Risk of bias: if there were “some concerns,” the evidence was downgraded by one level; if there was a “high risk” of bias, the evidence was downgraded by two levels.
- Inconsistency: when statistical heterogeneity was moderate (I² > 25%), the evidence was downgraded by one level; when heterogeneity was high (I² > 75%), the evidence was downgraded by two levels. If pooled results for the same outcome were inconsistent across different analyses, inconsistency was considered a serious concern; conversely, if no inconsistency was observed in the pooled outcome, it was not considered serious.
- Imprecision: when statistical power was < 80% and the direction of effect was unclear, the evidence was downgraded by one level.
- Publication bias: when Egger’s test showed *P* < 0.05, the evidence was downgraded by one level.

When applicable, evidence could be upgraded based on a large magnitude of effect, a dose–response relationship, and the presence of plausible residual confounding that would likely underestimate the true effect (i.e., negative bias).

In addition, to support overall methodological quality judgments, this study referenced the Oxford Centre for Evidence-Based Medicine (OCEBM) Levels of Evidence to stratify and categorize evidence. When multiple evidence sources were available for the same outcome, the principle of “prioritizing the totality of evidence” was applied: in general, findings from systematic reviews/meta-analyses were prioritized, followed by single primary studies (e.g., RCTs or observational studies), to achieve larger sample sizes and more stable effect estimates while minimizing duplicate reporting. Specifically, when both a meta-analysis and single-study results were available for the same outcome, the meta-analysis was cited preferentially; unless a single study provided critical information not covered by the meta-analysis (e.g., specific subgroups, unique outcomes, or longer follow-up), it was not presented separately. When multiple meta-analyses were available for the same outcome, all relevant meta-analyses were included after evaluating differences in populations, interventions, outcome definitions, and statistical methods, and an overall judgment was made based on between-study heterogeneity and clinical applicability. Ultimately, evidence certainty and recommendation strength were determined (as shown in Table 3).

**Table 3:** Summary of the evidence on treatment therapy for LBP

Experimental models	Type of evidence	Number of studies (Ref.)	Quality of the evidence	Grade	Scope
Cognitive functional therapy (CFT)	Systematic review, meta-analysis and RCT	2^12,13^	Ⅰ	⨁⨁⨁⨁	Mainly for patients presenting with maladaptive pain beliefs, fear-avoidance behaviors, activity avoidance, protective movement patterns, persistent functional deficits, lifestyle-related issues, or other psychosocial obstacles to recovery.
Pain neuroscience education (PNE)	Systematic review, meta-analysis and RCT	2^14,15^	Ⅰ	⨁⨁⨁⨁	Mainly for patients who are overly concerned about imaging findings, fear movement, catastrophize pain, show fear-avoidance behaviors, have features of central sensitization, or have reduced pain self-efficacy.
Self compassion therapy (SCT)	Systematic review, meta-analysis and RCT	1^16^	Ⅰ	⨁⨁⨁○	Mainly for patients with self-critical coping, emotional distress, pain catastrophizing, kinesiophobia, or reduced pain self-efficacy.
Physiotherapy-informed acceptance and commitment therapy (PACT)	Systematic review, meta-analysis and RCT	1^17^	II	⨁⨁⨁○	Mainly for patients with psychological inflexibility, activity avoidance, fear-avoidance beliefs, pain catastrophizing, or persistent functional disability.
Motivational interviewing (MI)	Systematic review, meta-analysis and RCT	2^18,19^	II	⨁⨁○○	Mainly for patients with poor adherence, unwillingness to exercise, a need for behavioral change, or a need for long-term self-management.
Mindfulness meditation	Systematic review, meta-analysis and RCT	2^20,21^	I/II	⨁⨁⨁○	Mainly for patients whose pain interferes with daily life, those with poor sleep, anxiety, maladaptive coping strategies, or refractory pain.
Core stabilization exercise	Systematic review, meta-analysis and RCT	2^22,23^	I/II	⨁⨁⨁○	Mainly for patients with poor movement control, impaired trunk muscle activation, poor lumbar stability, or recurrent low back pain.
Sling exercise	Systematic review, meta-analysis and RCT	2^24,25^	I/II	⨁⨁⨁○	Mainly for patients with poor core stability, poor movement control, suspected lumbar instability, or a need for progressive training.
Proprioceptive neuromuscular facilitation (PNF)	Systematic review, meta-analysis and RCT	1^26^	I	⨁⨁⨁○	Mainly for patients with impaired trunk proprioception, poor coordination, impaired balance, or poor muscular endurance.
McKenzie method	Systematic review, meta-analysis and RCT	2^27,28^	Ⅰ	⨁⨁⨁⨁	Mainly for patients showing directional preference or pain centralization, such as leg pain moving back toward the low back or markedly decreasing after certain movements.
Low-load blood flow restriction training (LL-BFRT)	Systematic review, meta-analysis and RCT	1^29^	Ⅱ	⨁⨁⨁○	Mainly for patients who cannot tolerate high-load strength training but need to improve core muscle strength or endurance.
Aquatic exercise	Systematic review, meta-analysis and RCT	2^30,31^	I	⨁⨁⨁⨁	Mainly for patients with obesity, older adults, those with marked pain, difficulty with land-based exercise, or poor physical capacity.
Pilates	Systematic review, meta-analysis and RCT	3^32–34^	Ⅰ	⨁⨁⨁○	Mainly for patients with poor core endurance, kinesiophobia, poor postural control, or a preference for gentle exercise. Patients with LBP should avoid excessive spinal flexion or extension movements.
Baduanjin or Qigong	Systematic review, meta-analysis and RCT	7^4,35–39^	I	⨁⨁⨁⨁	Mainly for patients who are unsuitable for vigorous exercise, have poor adherence, experience psychological stress, or prefer home-based exercise.
Tai Chi	Systematic review, meta-analysis and RCT	2^40,41^	I	⨁⨁⨁⨁	Mainly for patients who prefer gentle exercise, are older, have average or limited physical capacity, or need an exercise program that can be maintained long term.
Yoga	Systematic review, meta-analysis and RCT	2^42,43^	I	⨁⨁⨁○	Mainly for patients who prefer low-impact mind-body exercise and can receive standardized instruction. Patients with LBP should avoid excessive spinal flexion or extension movements.
Vojta therapy	Systematic review, meta-analysis and RCT	1^44^	Ⅱ	⨁⨁⨁○	Mainly for patients with marked functional limitation and poor trunk postural control who require treatment delivered by a trained therapist.
Feldenkrais	Systematic review, meta-analysis and RCT	1^45^	Ⅱ	⨁⨁⨁○	Mainly for patients with impaired body awareness, movement stiffness, or functional limitation.
Connective tissue massage (CTM)	Systematic review, meta-analysis and RCT	2^46,47^	Ⅱ	⨁⨁⨁○	Mainly for patients with limited lumbar mobility and reduced health-related comfort or well-being.
Spinal manipulative therapy	Systematic review, meta-analysis and RCT	3^48–50^	Ⅰ	⨁⨁⨁○	Mainly for patients with chronic non-specific low back pain; it is best used together with exercise therapy.
Visceral manipulation (VM)	Systematic review, meta-analysis and RCT	1^51^	Ⅱ	⨁⨁⨁○	Mainly for selected patients with coexisting visceral dysfunction or suspected visceral contributing factors.
Myofascial release	Systematic review, meta-analysis and RCT	3^52–54^	I	⨁⨁○○	Mainly for patients with marked muscle or fascial tightness, functional limitation, or restricted mobility.
Tuina	Systematic review, meta-analysis and RCT	3^55–57^	I	⨁⨁○○	Mainly for patients who cannot immediately perform active exercise or who prefer adjunctive manual therapy.
Instrument-assisted soft tissue mobilization (IASTM)	Systematic review, meta-analysis and RCT	2^58,59^	I/II	⨁⨁○○	Mainly for patients with soft-tissue tightness or restricted mobility; it should not be used as the core treatment.
Moxibustion	Systematic review, meta-analysis and RCT	2^60,61^	Ⅰ	⨁⨁⨁○	Mainly for patients with chronic non-specific low back pain who can accept heat-based therapy or traditional Chinese medicine treatment.
Acupuncture	Systematic review, meta-analysis and RCT	2^62,63^	Ⅰ	⨁⨁⨁⨁	Mainly for patients with chronic non-specific low back pain who prefer a non-pharmacological analgesic option.
Dry needling	Systematic review, meta-analysis and RCT	2^64,65^	I	⨁⨁○○	Mainly for patients with chronic low back pain and clearly identifiable myofascial trigger points.
Transcutaneous electrical nerve stimulation (TENS)	Systematic review, meta-analysis and RCT	3^66–68^	I	⨁⨁⨁○	Mainly for patients seeking short-term analgesia, those intolerant of medication, or those requiring an adjunct to rehabilitation.
Transcranial direct current stimulation (tDCS)	Systematic review, meta-analysis and RCT	3^69–71^	I/II	⨁⨁⨁○	Mainly for patients with refractory chronic low back pain; it may be considered especially when combined with exercise therapy.
Peripheral nerve stimulation (PNS)	Systematic review, meta-analysis and RCT	1^72^	Ⅱ	⨁⨁○○	Mainly for patients with refractory chronic low back pain after failed conservative treatment.
Intradiscal Orthobiological Injections (PRP)	Systematic review, meta-analysis and RCT	2^73,74^	Ⅰ	⨁⨁⨁○	Mainly for highly selected patients with discogenic chronic low back pain after failed conservative treatment.
Intradiscal mesenchymal stem cell injection	Systematic review, meta-analysis and RCT	3^75–77^	Ⅰ	⨁⨁○○	Mainly for highly selected patients with discogenic low back pain; it is preferably performed within ethically approved or registered clinical studies.
Non-steroidal anti-inflammatory drugs (NSAIDs)	Systematic review, meta-analysis and RCT	2^78,79^	Ⅰ	⨁⨁⨁⨁	Mainly for patients with mild-to-moderate pain, prominent localized pain, high risk from oral medication, or a desire to reduce systemic drug exposure.
Ibuprofen + paracetamol fixed-dose combination	Systematic review, meta-analysis and RCT	1^80^	Ⅰ	⨁⨁⨁○	Mainly for patients with acute low back pain requiring short-term pharmacological analgesia; hepatic, renal, and gastrointestinal risks should be considered.
Opioids	Systematic review, meta-analysis and RCT	2^81,82^	Ⅰ	⨁⨁⨁⨁	Should not be used routinely, except as low-dose, short-term rescue therapy for acute severe pain when NSAIDs are contraindicated or ineffective.
Muscle relaxants	Systematic review, meta-analysis and RCT	1^83^	I	⨁⨁⨁○	Mainly for patients with acute non-radicular low back pain accompanied by muscle spasm; long-term reliance is not recommended.
Serotonin and norepinephrine reuptake inhibitors (SNRIs)	Systematic review, meta-analysis and RCT	1^84^	I	⨁⨁⨁○	Mainly for patients with chronic non-specific low back pain, especially those with comorbid emotional problems, sleep problems, or widespread pain.
Biologic agents	Systematic review, meta-analysis and RCT	1^85^	Ⅰ	⨁⨁○○	Not suitable for routine use in common chronic low back pain.
Preoperative prehabilitation	Systematic review, meta-analysis and RCT	1^86^	Ⅰ	⨁⨁○○	Mainly for patients awaiting elective lumbar spine surgery who have a high risk of poor recovery or may recover slowly.
Individualized homeopathic (IH)	Systematic review, meta-analysis and RCT	1^87^	Ⅰ	⨁⨁○○	Should only be considered within well-designed clinical studies.
Transforaminal epidural steroid injection (TFESI)	Systematic review, meta-analysis and RCT	2^88,89^	Ⅰ/Ⅱ	⨁⨁⨁○	Mainly for patients with acute severe sciatica or marked nerve-root inflammation.
Radiofrequency denervation	Systematic review, meta-analysis and RCT	1^90^	Ⅰ	⨁⨁⨁○	Mainly for patients with confirmed chronic low back pain originating from the facet joints.
Sacroiliac joint (SIJ)	Systematic review, meta-analysis and RCT	1^91^	I	⨁⨁○○	Mainly for patients with confirmed sacroiliac joint-mediated low back pain.
Trigger point injection (TPI)	Systematic review, meta-analysis and RCT	1^92^	I	⨁⨁⨁○	Mainly for patients with acute low back pain and clearly identifiable myofascial trigger points.
Intradiscal glucocorticoid injection (IGI)	Systematic review, meta-analysis and RCT	2^93,94^	Ⅰ	⨁⨁⨁○	Mainly for patients with chronic discogenic low back pain accompanied by active discopathy or Modic changes.
Ozone injection	Systematic review, meta-analysis and RCT	2^95,96^	I/II	⨁⨁○○	Mainly for patients with lumbar disc herniation-related radicular low back and leg pain or sciatica after failed conservative treatment.
Percutaneous intradiscal radiofrequency thermocoagulation (PIRFT)	Systematic review, meta-analysis and RCT	2^97,98^	Ⅰ	⨁⨁○○	Not recommended for routine use in non-specific low back pain or ordinary discogenic low back pain.
Intradiscal electrothermal therapy (IDET)	Systematic review, meta-analysis and RCT	1^99^	I	⨁⨁○○	Should only be used in highly selected patients under research, audit, or strict governance conditions.
Plasma disc decompression (PDD)	Systematic review, meta-analysis and RCT	1^100^	Ⅰ	⨁⨁○○	Mainly for patients with sciatica related to contained disc herniation when open surgery is unsuitable or not currently being considered.
Spinal cord stimulation (SCS)	Systematic review, meta-analysis and RCT	2^101,102^	Ⅰ	⨁⨁⨁⨁	Mainly for patients with refractory low back and leg pain, failed back surgery syndrome, neuropathic or radicular pain, and successful trial stimulation.
Pulsed radiofrequency	Systematic review, meta-analysis and RCT	2^103,104^	Ⅰ	⨁⨁⨁○	Mainly for patients with refractory lumbar radicular pain or sciatica after failure of conservative treatment or epidural injection.
Dorsal root ganglion stimulation (DRGS)	Systematic review, meta-analysis and RCT	2^105,106^	I/II	⨁⨁○○	Mainly for patients with refractory postoperative radicular pain, focal lumbosacral neuropathic pain, or lower-extremity complex regional pain syndrome (CRPS).
Peripheral nerve field stimulation	Systematic review, meta-analysis and RCT	2^107,108^	Ⅰ	⨁⨁○○	Mainly for patients with persistent postoperative pain or refractory axial low back pain and a successful trial stimulation.
Endoscopic discectomy (ED)	Systematic review, meta-analysis and RCT	2^109,110^	Ⅰ	⨁⨁⨁○	Mainly for patients with clear radicular low back and leg pain caused by lumbar disc herniation, failed conservative treatment, and concordant symptoms and imaging findings.
Total disc replacement (TDR)	Systematic review, meta-analysis and RCT	2^111,112^	Ⅰ/Ⅱ	⨁⨁⨁○	Mainly for highly selected patients with discogenic low back pain, failed conservative treatment, and no obvious contraindications.
Lumbar spinal decompression	Systematic review, meta-analysis and RCT	2^113,114^	Ⅰ	⨁⨁⨁○	Mainly for patients with lumbar spinal stenosis-related neurogenic claudication or sciatica.
Lumbar interbody fusion (LIF)	Systematic review, meta-analysis and RCT	2^115,116^	Ⅰ	⨁⨁⨁○	Mainly for patients with chronic discogenic pain, degenerative spondylolisthesis, spinal instability, recurrent disc herniation with instability, deformity, pseudoarthrosis, or similar conditions.
Sacroiliac joint fusion	Systematic review, meta-analysis and RCT	2^117,118^	Ⅰ	⨁⨁⨁○	Mainly for patients with confirmed sacroiliac joint-mediated pain after failed conservative treatment, especially degenerative sacroiliitis or sacroiliac pain after lumbar fusion.
Multidisciplinary biopsychosocial rehabilitation	Systematic review, meta-analysis and RCT	2^119,120^	Ⅰ	⨁⨁⨁⨁	Mainly for patients with a long disease duration, recurrent episodes, poor function, a high risk of poor recovery, or an inadequate response to single-modality treatment.
Physiotherapy combined with structured patient education	Systematic review, meta-analysis and RCT	3^23,121,122^	Ⅰ	⨁⨁⨁⨁	Mainly for patients with persistent, recurrent, or chronic low back pain, poor self-management ability, or concerns about recurrence.
Physiotherapist-delivered cognitive-behavioral interventions	Systematic review, meta-analysis and RCT	2^123,124^	Ⅰ	⨁⨁⨁⨁	Mainly for patients with fear-avoidance, low self-efficacy, psychosocial barriers, or a high risk of poor recovery.
Group-based exercise and education intervention	Systematic review, meta-analysis and RCT	2^125,126^	Ⅰ	⨁⨁⨁⨁	Mainly for patients who need to improve self-management, exercise confidence, physical activity, and functional recovery.
Risk stratification combined with shared decision-making	Systematic review, meta-analysis and RCT	2^127,128^	Ⅰ	⨁⨁⨁○	Applicable to all patients with low back pain, especially those with a long disease duration, frequent recurrence, or complex treatment choices.
Risk-stratified tailored care	Systematic review, meta-analysis and RCT	2^127,129^	Ⅰ	⨁⨁⨁○	Mainly for patients who require different treatment intensities according to risk level, function, and psychosocial factors.
Back school	Systematic review, meta-analysis and RCT	2^130,131^	I	⨁⨁⨁○	Mainly for patients who need to improve disease understanding, self-management, and recurrence prevention.
Digital support interventions for the self-management	Systematic review, meta-analysis and RCT	2^132,133^	Ⅰ	⨁⨁⨁○	Mainly for patients undergoing home-based rehabilitation, requiring long-term follow-up, or showing poor adherence.
Stratified blended physiotherapy	Systematic review, meta-analysis and RCT	2^134,135^	Ⅰ	⨁⨁⨁○	Mainly for patients who cannot frequently attend in-person visits, require long-term management, or face high follow-up costs.
Workplace interventions ergonomic interventions	Systematic review, meta-analysis and RCT	2^136,137^	Ⅰ	⨁⨁⨁○	Mainly for sedentary office workers, manual laborers, or patients with work-related low back pain.
Digital therapeutics using artificial intelligence-assisted telerehabilitation	Systematic review, meta-analysis and RCT	2^138,139^	Ⅰ	⨁⨁⨁○	Mainly for patients suitable for remote management and those with a high need for movement feedback.
Wearable devices	Systematic review, meta-analysis and RCT	2^140,141^	Ⅰ	⨁⨁○○	Mainly for patients with sedentary behavior, low activity levels, or a need for exercise feedback and goal management.

⨁⨁⨁⨁: high level of evidence(A), ⨁⨁⨁○: moderate level of evidence(B), ⨁⨁○○: low level of evidence(C), ⨁○○○: very low level of evidence(D)

### Development of the consensus recommendations

The development of the recommendations in this consensus strictly followed the GRADE Evidence-to-Decision (EtD) framework and was complemented by the Oxford Centre for Evidence-Based Medicine (OCEBM) levels of evidence to stratify and categorize evidence types, ensuring a systematic, transparent, and traceable recommendation process. The EtD framework integrated clinical research evidence and its certainty ratings and, together with multidisciplinary expert input and professional experience, systematically evaluated key dimensions including the balance of benefits and harms, certainty of evidence, patients’/clinical stakeholders’ values and preferences, resource use and costs, potential impacts on health equity, and clinical acceptability and feasibility. Each recommendation was based on comprehensive review and synthesis of the evidence for critical outcomes, followed by multidisciplinary panel discussion and structured voting to determine the final recommendation strength. In general, a recommendation was classified as a strong recommendation only when a higher proportion of experts endorsed it (e.g., ≥ 75% agreement); otherwise, it was considered a conditional recommendation.

**Fig. 3:**
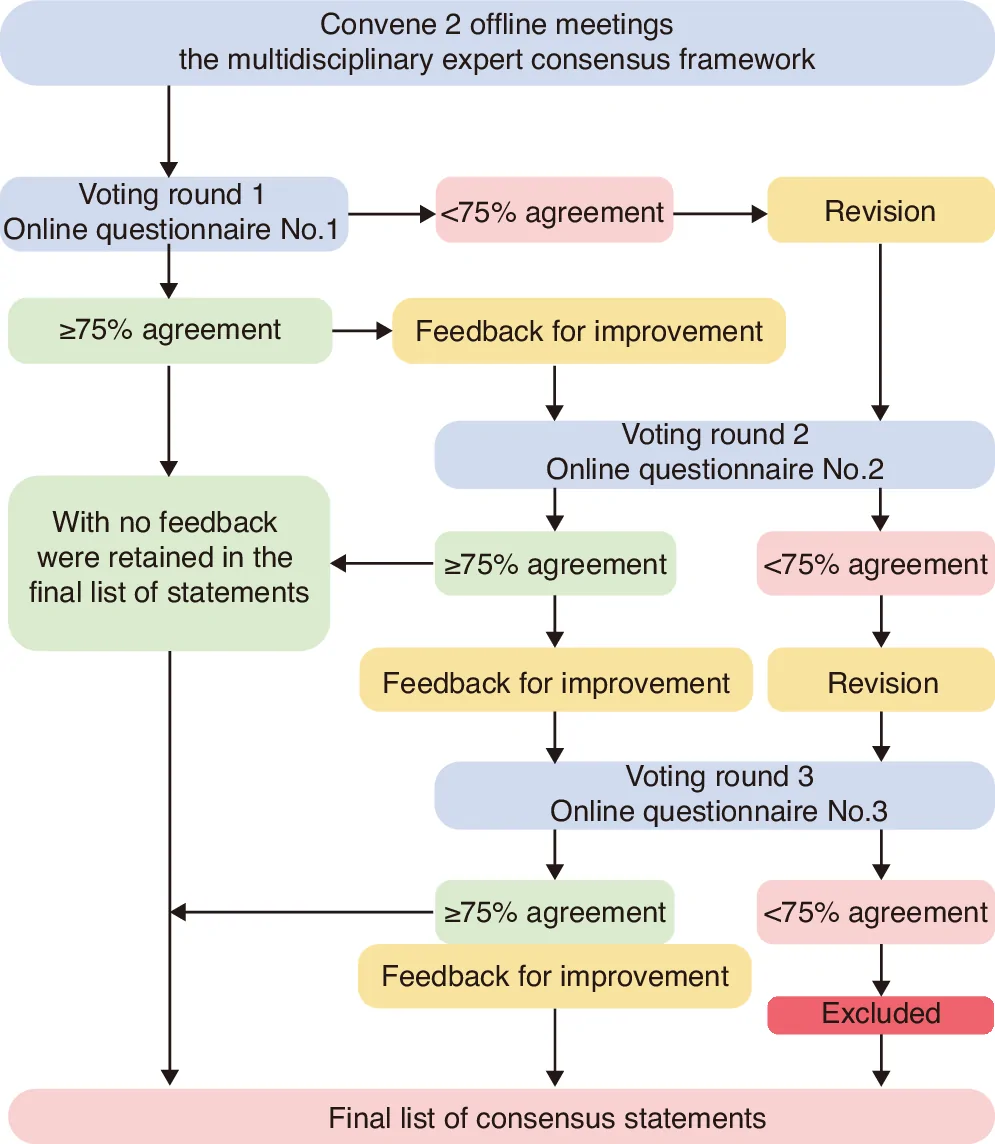
Modified Delphi voting and revision process for consensus statement development. After two in-person meetings to establish the multidisciplinary consensus framework, draft statements entered Voting Round 1 (online questionnaire). Items with ≥ 75% agreement were retained for the final statement list (no further revision required). Items with <75% agreement were revised based on panel feedback and advanced to Voting Round 2 (online questionnaire). Statements that still showed <75% agreement underwent additional revision and were discussed in Voting Round 3 (online consensus meeting). Following the meeting, statements achieving consensus (≥75% agreement / ≤ 25% disagreement) were included in the final list, whereas those with persistent <75% agreement were excluded

The consensus development group first convened two in-person meetings for thorough discussion and drafted a preliminary framework for the multidisciplinary expert consensus on clinical diagnosis and treatment models for low back pain, incorporating patients’ clinical presentations as well as surgical and imaging information. Subsequently, multidisciplinary experts were invited to participate in the consensus development process. Using structured elicitation and voting procedures, each proposed recommendation was evaluated and revised item by item to generate consensus-based recommendation ratings. Specifically, a modified Delphi questionnaire process was conducted, with 2–3 rounds of anonymous surveys to collect expert opinions and iteratively revise statements based on feedback. In each round, agreement with each statement was quantitatively assessed. A response rate of ≥ 75% was required to ensure reliability; questionnaires with incomplete ratings were considered invalid and were not included in the calculation of agreement. For each intervention, consensus was defined as an agreement rate ≥ 75%. Items that did not reach consensus were revised after synthesizing expert feedback and entered the next voting round. After the two rounds of feedback–revision–revoting, a structured online consensus meeting was held to discuss remaining items that had not reached consensus, resulting in the final set of concordant recommendations as shown in Fig. 3. All working group members reviewed and approved the grading definitions, the final recommendations, and the final manuscript version, and collectively take responsibility for the content of the consensus.

## Characteristics of the consensus expert panel

**Fig. 4:**
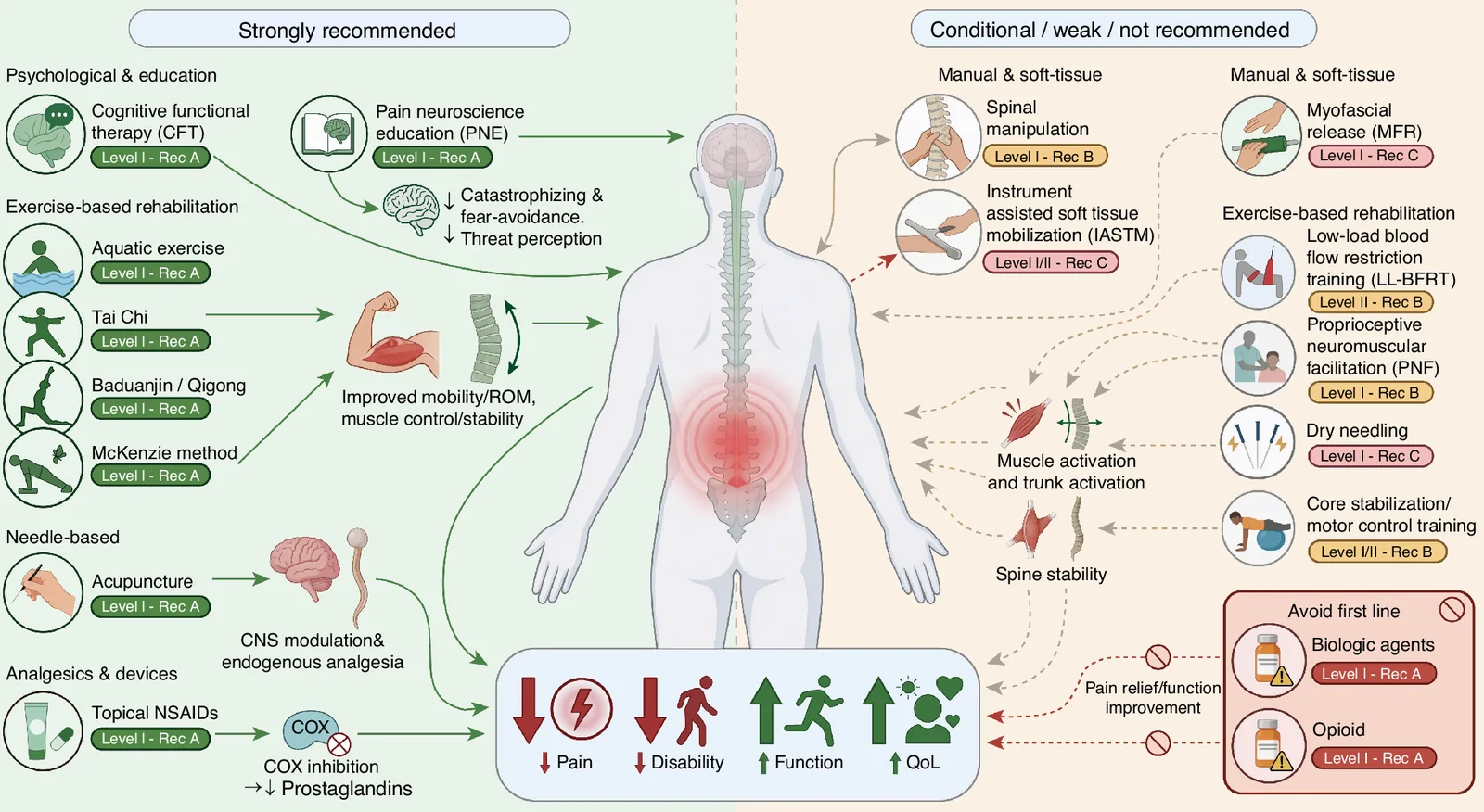
Graded recommendations for conservative therapy of low back pain. The figure summarizes conservative interventions for low back pain according to recommendation strength and level of evidence. It provides an overview of non-surgical interventions and classifies them by the strength of recommendation and supporting evidence. The left panel lists strongly recommended interventions, including cognitive functional therapy, pain neuroscience education, aquatic exercise, Tai Chi, Baduanjin/Qigong, the McKenzie method, acupuncture, and topical non-steroidal anti-inflammatory drugs. These interventions target multiple therapeutic mechanisms, including reduced pain-related threat appraisal, improved mobility and trunk stability, central pain modulation, endogenous analgesia, and inhibition of prostaglandin-mediated nociception. The right panel lists interventions with limited, weak, or insufficient effects, including spinal manipulation, myofascial release, instrument-assisted soft-tissue mobilization, low-load blood flow restriction training, proprioceptive neuromuscular facilitation, dry needling, and core stabilization or motor control training. Biologic agents and opioids may be considered only as alternative options in selected cases and should not be used as first-line treatments

To ensure the smooth implementation and high-quality completion of this consensus, the project team planned to establish four working groups responsible for evidence retrieval and appraisal, formulation of key questions and recommendations, methodological and statistical support, and manuscript drafting and overall editing, respectively. Each group has clearly defined roles and works collaboratively under a unified workflow and timeline management to ensure that the consensus development process is standardized and transparent and that the outcomes are scientifically robust and reliable (Fig. 4).

### Consensus steering committee

The Consensus Steering Committee consists of four members selected based on their national recognition and authority, comprising experts in spine surgery and consensus methodology. The main responsibilities of the Steering Committee are to: (1) define the topic and scope of the consensus; (2) establish other consensus working groups and manage their declarations of interests; (3) approve the consensus protocol; (4) oversee the consensus development process; (5) approve the recommendations and the full consensus manuscript; and (6) monitor and evaluate the need for future updates. The Steering Committee will appoint one Chief Expert.

### Consensus expert panel and characteristics

The consensus expert panel ultimately comprised 99 experts with experience in evidence appraisal and consensus development. Experts were selected from relevant disciplines—including orthopedics, sports medicine, rehabilitation medicine, pain medicine, nutritional medicine, psychology/psychiatry, and geriatrics—and were required to have more than 15 years of sustained clinical experience in the diagnosis, treatment, and management of low back pain. Panel members were drawn from eastern, central, and western regions of China to ensure geographic representativeness and a multidisciplinary perspective. All experts held doctoral degrees and were recognized leaders in their respective fields. Geographically, their primary institutional affiliations spanned 24 provinces across China, reflecting diverse regional cultures and academic backgrounds. The panel size was adequate to support robust consensus formation, and the diversity in geography and specialties strengthened the rigor of the process. This collaborative effort ensured that the final consensus reflects the collective expertise and perspectives of leading professionals in the field.

### Consensus development group

The consensus development group consisted of a secretariat and an evidence appraisal team, with 20 members primarily from the initiating institutions. This group was fully responsible for coordination, management, and development of the consensus, including: (1) completing consensus registration; (2) developing the protocol framework under the guidance of the Steering Committee; (3) collecting and organizing the literature; (4) conducting the online questionnaire surveys and compiling results; (5) drafting, revising, and refining the consensus manuscript (including literature searches, evidence appraisal, and application of the GRADE system); (6) coordinating the consensus development process (including in-person and online expert meetings); and (7) handling all matters not covered by other working groups. The head of the consensus development group also served as the consensus coordinator, responsible for coordinating all groups and guideline developers.

### External review panel

The external review panel consisted of four members. It comprised experts or relevant personnel who were associated with the topic of this consensus but did not participate in the development of the guideline/consensus. The panel’s primary responsibility was to review the initial draft and provide comments and suggestions. The work of the external review panel was completed before the official publication of the consensus.

### Specific voting process of the consensus expert panel

This consensus used a modified Delphi process,^7^ with 2–3 rounds planned to achieve agreement. In practice, two rounds of anonymous Delphi voting were completed: the first round was conducted to solicit initial feedback, and the second round was carried out after revising items based on that feedback. During voting, experts drew on their clinical experience and the evidence summaries provided by the consensus development group—including study findings, certainty/quality assessments, and levels of evidence—to independently select the strength of recommendation for each statement and provide comments. The development group then compiled and analyzed the ratings and qualitative feedback and quantitatively assessed agreement for each recommendation. Agreement was defined as the proportion of experts selecting the same recommendation category. When the agreement rate was ≥ 75%, the statement was considered to have reached strong consensus and was finalized. Statements that did not meet this threshold (<75%) were revised as needed in response to expert feedback and re-evaluated in subsequent rounds to progressively improve clarity, feasibility, and consistency.

The strength of recommendations was determined based on the totality of evidence and an overall judgment that also considered the balance of benefits and harms, patients’ values and preferences, resource use and costs, and acceptability and feasibility. Recommendation strength was expressed using four categories:

- Strong recommendation: Evidence indicates that the intervention provides clear benefits that substantially outweigh the risks and is applicable to most of the target population.
- Weak recommendation: Benefits are likely to outweigh risks; however, due to population heterogeneity, differences in preferences, or limitations in resource availability, the intervention is appropriate for selected populations and/or requires individualized decision-making.
- Weak recommendation against: Risks may outweigh benefits; therefore, routine use is generally not advised, but it may be considered with caution in specific circumstances.
- Strong recommendation against: Evidence indicates that risks clearly outweigh benefits, and the intervention should be avoided in the target population.

## Definitions and classification of low back pain terminology

### Definition

Low back pain is a common musculoskeletal symptom that may involve the muscles, ligaments, bones, and related neural structures of the lumbar region. Most guidelines define its anatomical boundaries as the area from below the costal margin (the 12th rib) to above the gluteal folds, with or without accompanying leg pain.^8^ Low back pain is a symptom description rather than a single disease diagnosis; therefore, clinical evaluation should integrate symptom duration, physical findings, and red-flag features to determine whether a specific underlying cause is present.

### Classification by symptom duration

Low back pain is commonly categorized according to symptom duration as acute (<6 weeks), subacute (6–12 weeks), and chronic (>12 weeks, i.e., longer than 3 months). In this consensus, subsequent recommendations referring to “chronic low back pain (CLBP)” or “chronic non-specific low back pain (CNLBP)” adopt > 12 weeks as the threshold for chronicity, and each recommendation will specify whether leg pain and/or radicular symptoms are present.^9^

### Classification by physiological system

To improve the accuracy of identifying pain sources in clinical practice, low back pain is commonly classified according to the underlying physiological system into the following categories^10^:

1. Non-specific low back pain (NSLBP): this category accounts for approximately 95% of all low back pain cases. Because the etiology is unclear in most patients, imaging examinations such as X-ray and CT often yield no specific positive findings, and classification in clinical practice is frequently applied only in a broad manner. Many clinical practice guidelines further subdivide NSLBP into several subtypes, such as facet joint–related, disc-related, instability-related, sacroiliac joint–related, postural, and non-anatomical subtypes.
2. Radicular low back pain: this type results from nerve root compression. Pain radiates along the nerve distribution into the leg and is often accompanied by numbness, tingling, or weakness, such as in cases caused by lumbar disc herniation or spinal stenosis.
3. Other pathological types of low back pain: this category may indicate the presence of serious underlying causes, including low back pain due to fracture, infection, tumor, or inflammatory spinal disease. It represents a relatively small proportion of the low back pain population. Clinicians should integrate red-flag features and appropriate ancillary examinations for further evaluation and differential diagnosis.

### Classification by specific pain source

Based on clinical presentation, physical examination findings, and necessary ancillary tests, the “likely pain sources and pathophysiological mechanisms” of low back pain can be categorized as follows^6,11^:

1. Mechanical pain: this is the most common clinical type. Pain onset and severity are closely related to postural changes, physical loading, and lumbar movement. Symptoms typically worsen with forward bending, prolonged sitting or standing, or loading, and are markedly relieved by bed rest or postural adjustment. The pathogenesis is often associated with biomechanical or structural abnormalities of the spine and surrounding soft tissues. Common related conditions include lumbar disc degeneration, facet joint dysfunction, lumbar muscle strain, and sacroiliac joint instability, most of which fall within the scope of non-specific low back pain described above.
2. Non-mechanical pain: the association between pain and lumbar movement is atypical. In some patients, symptoms do not change significantly after activity, and pain may even be more prominent at rest. Non-mechanical pain is often accompanied by systemic symptoms or specific signs, warranting a high index of suspicion for an underlying specific cause. It is commonly triggered by inflammation, infection, malignancy, metabolic diseases, and related conditions. Examples include inflammatory back pain due to ankylosing spondylitis (prominent morning stiffness with transient relief after movement) and persistent pain caused by spinal infection or tumor (notable night pain and rest pain). Further evaluation should integrate laboratory tests (e.g., inflammatory markers) and imaging studies.
3. Referred pain: pain manifests as low back discomfort, but the primary lesion is not located in the spine or surrounding soft tissues; instead, abnormalities in other organs or distant structures refer pain to the low back via neural reflex pathways. Common causes include pelvic organ disorders (e.g., pelvic inflammatory disease, prostate disorders) and abdominal visceral conditions (e.g., renal calculi, pancreatitis). Key differentiating features include the absence of a clear localized lumbar tenderness point, lack of correlation with lumbar mechanical activity, accompanying symptoms related to the primary disorder (e.g., urinary frequency/urgency, abdominal pain, fever), and poor response to symptomatic treatments targeting the low back. Diagnosis should be confirmed through careful history-taking and targeted investigations for the suspected primary condition.

Based on the above classification by etiology, symptom duration, and pain source, clinical management should adhere to the core principle of “identify the cause first, then implement stepped interventions.” Conservative, interventional, surgical, and novel treatment modalities should be selected in an individualized manner according to disease severity, symptom type, and underlying pathology. Conservative management, as a noninvasive and safe first-line strategy, is primarily applicable to most acute and subacute low back pain cases and to chronic non-specific low back pain without clear surgical indications. Through physical therapy, pharmacologic interventions, and rehabilitation training, conservative treatment can effectively alleviate symptoms and restore lumbar biomechanical function and thus constitutes the foundation of clinical care. For patients who do not respond to conservative therapy and whose symptoms continue to progress, interventional treatments may be considered, such as nerve root blocks and intradiscal ablation—minimally invasive approaches that target the pathological site while balancing efficacy and safety. Surgical treatment is mainly reserved for cases with definite structural pathology and clear surgical indications, such as severe disc herniation, spinal stenosis, spinal instability, or low back pain caused by specific etiologies including tumor or infection, with the goals of relieving neural compression, restoring spinal structure, and controlling the primary lesion. In addition, with advances in medical technology, emerging interventions such as stem cell therapy and targeted pharmacotherapy are being explored for refractory low back pain, providing potential options for selected patients who respond poorly to conventional treatments. Importantly, accumulating evidence suggests that degenerative remodeling of the cartilaginous and bony endplate may precede, accompany, or amplify disc failure. Therefore, discogenic low back pain should not be interpreted solely as an isolated disc disorder. In clinical decision-making, the intervertebral disc and endplate should be considered an integrated functional unit. Accordingly, disc-targeted interventions—particularly intradiscal biologic or regenerative therapies—should incorporate evaluation of endplate integrity, nutrient transport capacity, and concomitant endplate lesions, because advanced endplate sclerosis, calcification, or porous remodeling may limit biological responsiveness even when disc pathology appears radiologically prominent. Overall, low back pain management should follow an integrated framework in which conservative care serves as the foundation, interventional therapy as an adjunct, surgery for severe or clearly indicated cases, and novel approaches as potential breakthroughs—aligning interventions with the above classifications to maximize efficacy while minimizing invasiveness (Fig. 4).

## Key questions related to low back pain treatment and recommendations for consensus

### Conservative therapy: cornerstone and core strategy for first-line interventions

Traditional therapy serves as the preferred first-line management strategy for most patients with acute or subacute low back pain and for many patients with chronic nonspecific low back pain without clear structural indications for invasive intervention. This treatment level should generally be initiated once serious specific pathology has been excluded and individualized according to symptom characteristics, functional limitation, and psychosocial context. Clinical response should be reassessed after an appropriate short-term treatment period, focusing on pain relief, functional improvement, treatment adherence, and patient-reported benefit. If symptoms fail to improve adequately, worsen progressively, or if new red-flag or structural features emerge, escalation to interventional assessment or referral for more specialized evaluation should be considered.

#### Recommendation

Cognitive Functional Therapy (CFT) is strongly recommended as an individualized biopsychosocial rehabilitation option for selected patients with chronic nonspecific low back pain, particularly those with maladaptive pain beliefs, fear-avoidance behaviors, activity avoidance, protective movement patterns, persistent functional disability, lifestyle-related contributors, or other psychosocial barriers to recovery.

CFT is a physiotherapist-led, individualized intervention that integrates pain education, cognitive reassurance, controlled exposure to feared or avoided movements, functional movement retraining, graded physical activity, and lifestyle coaching. Kent et al. conducted the RESTORE trial, a randomized, controlled, three-arm, parallel-group phase 3 clinical trial involving 492 patients with chronic disabling low back pain. At 13 weeks, both CFT alone and CFT with biofeedback produced significantly greater improvements in activity limitation than usual care, with a mean difference of − 4.6 points on the Roland-Morris Disability Questionnaire in both intervention groups. The treatment effects were sustained at 52 weeks, and the addition of movement sensor biofeedback did not provide additional clinical benefit over CFT alone. Moreover, CFT was associated with improved quality-adjusted life-years and lower societal costs compared with usual care.^12^ Thiveos et al. further performed a systematic review and meta-analysis including 7 randomized controlled trials with 1011 participants.^13^ These findings suggest that CFT is particularly suitable for patients whose persistent low back pain is maintained by cognitive-behavioral, functional, emotional, or lifestyle-related factors. Based on the available Level I evidence, its favorable safety profile, sustained functional benefits, and strong alignment with the biopsychosocial mechanisms underlying persistent low back pain, CFT is recommended for appropriately selected patients when delivered by trained clinicians using standardized clinical reasoning and treatment fidelity procedures. Therefore, CFT is strongly recommended for selected patients with chronic nonspecific low back pain with prominent cognitive-behavioral, functional, or lifestyle-related contributors to disability (Level I evidence, Grade A recommendation).

#### Recommendation

Pain neuroscience education (PNE) is strongly recommended as part of a multimodal rehabilitation program for selected patients with chronic nonspecific low back pain, particularly those with maladaptive pain beliefs, pain catastrophizing, kinesiophobia, fear-avoidance behaviors, central sensitization features, or reduced pain self-efficacy

Pain neuroscience education (PNE) is an educational intervention that explains the neurobiology and neurophysiology of pain, aiming to help patients reconceptualize pain as a less threatening experience rather than a direct marker of tissue damage. Ma et al. conducted a systematic review and meta-analysis including 17 randomized controlled trials with 1 078 patients with chronic low back pain. Compared with exercise or physical therapy alone, adding PNE produced greater short-term improvements in pain and disability. PNE combined with exercise reduced pain by 1.14 points on a 0–10 scale and disability by an SMD of − 0.80, while PNE combined with physical therapy reduced pain by 1.15 points and disability by an SMD of − 0.85. The review also suggested that sessions longer than 60 minutes, 4–8 total sessions, a 7–12-week intervention period, and group-based delivery may be associated with greater analgesic effects.^14^ Medina Viedma et al. further performed a systematic review and meta-analysis including 15 randomized controlled trials with 810 patients. PNE significantly reduced pain, disability, kinesiophobia, and pain catastrophizing immediately after treatment and during follow-up, with moderate-to-large effect sizes across several outcomes.^15^ These findings suggest that PNE is particularly suitable for patients with maladaptive pain beliefs, pain catastrophizing, kinesiophobia, fear-avoidance behaviors, central sensitization features, or reduced pain self-efficacy. Based on the available Level I evidence, its low-risk and low-cost profile, and its consistent benefits when integrated with exercise or physical therapy, PNE is strongly recommended as part of a multimodal rehabilitation program for selected patients with chronic nonspecific low back pain (Level I evidence, Grade A recommendation).

#### Recommendation

Self compassion therapy (SCT) may be considered as a weakly recommended adjunctive psychological intervention for selected patients with chronic nonspecific low back pain, particularly those with self-critical coping, emotional distress, pain catastrophizing, kinesiophobia, or reduced pain self-efficacy.

SCT is a structured, therapist-guided approach that cultivates self-kindness, common humanity, and mindfulness through techniques such as loving-kindness meditation, self-compassion psychoeducation, and compassionate self-reflection. In this systematic review of 4 randomized or longitudinal clinical trials (total *n* = 112), SCT consistently demonstrated benefits across pain and mental health outcomes. Berry et al. reported significant reductions in pain intensity and disability, alongside increased self-compassion. Chapin et al. observed large effect sizes for pain intensity reduction and pain acceptance improvement. Zheng et al. found moderate anxiety reduction in patients combining SCT with core stability exercise, while Carson et al. (2005) documented decreased psychological distress and pain intensity. Interventions ranged from 2 to 9 weeks, with session durations of 90 min to 6 h, and were delivered by trained instructors.^16^ Given its reproducibility, safety profile, and alignment with biopsychosocial pain mechanisms, SCT is recommended as a complementary non-pharmacological option to enhance pain management, emotional regulation, and functional recovery in chronic low back pain care (Level I evidence, Grade B recommendation).

#### Recommendation

Physiotherapy-informed acceptance and commitment therapy (PACT) may be considered as a weakly recommended adjunctive rehabilitation option for selected patients with chronic low back pain, particularly those with psychological inflexibility, activity avoidance, fear-avoidance beliefs, pain catastrophizing, or persistent functional disability.

PACT is a physiotherapist-delivered, psychologically informed rehabilitation intervention that integrates principles of acceptance and commitment therapy into routine physical therapy practice. It aims to improve function and self-management by shifting the therapeutic focus from pain elimination to valued activity participation, psychological flexibility, acceptance-based coping, individualized exercise prescription, and reduction of avoidance-related disability. Godfrey et al. conducted a phase II, assessor-blinded, two-arm, multicenter randomized controlled trial comparing PACT with usual care physical therapy in 248 adults with chronic low back pain. At 3 months, PACT produced a significantly greater improvement in back pain-related disability than usual care, with an adjusted mean difference of − 1.07 points on the Roland-Morris Disability Questionnaire. PACT was also associated with better patient-specific functioning, physical health, work and social adjustment, and treatment credibility at 3 months. However, these between-group differences were not sustained at 12 months, and no significant advantages were observed for pain intensity, mood, self-efficacy, pain acceptance, or committed action.^17^ These findings suggest that PACT may be suitable for selected patients whose disability is influenced by psychological inflexibility, fear-avoidance beliefs, activity avoidance, pain catastrophizing, or difficulty engaging in valued activities. Given its modest short-term effect, lack of sustained superiority at 12 months, and the need for trained physiotherapists, PACT may be considered as a weakly recommended adjunctive rehabilitation option for selected patients with chronic low back pain and prominent psychological or behavioral barriers to functional recovery (Level II evidence, Grade B recommendation).

#### Recommendation

Integrating Motivational Interviewing (MI) into multimodal treatment can be considered as a weak recommendation for patients with chronic non-specific low back pain; however, its advantage over standard care for patients with subacute low back pain remains unclear.

MI is a patient-centered counseling strategy designed to enhance intrinsic motivation, resolve ambivalence, support self-efficacy, and promote health-related behavior change. In chronic nonspecific low back pain, MI is usually delivered as an adjunct to multimodal rehabilitation rather than as a stand-alone treatment, and may be integrated with exercise therapy, pain education, manual therapy, home-based activity programs, or conventional physiotherapy to improve adherence and self-management. Akinrolie et al. conducted a systematic review and meta-analysis including 3 randomized controlled trials with 175 adults with chronic low back pain. Compared with control interventions, MI combined with exercise did not significantly improve pain intensity, disability, or physical functioning.^18^ Hardt et al. further reviewed MI for musculoskeletal pain and found that, although some studies suggested possible short-term benefits, the overall evidence remained insufficient, with most studies showing high risk of bias and low to very low certainty of evidence.^19^ These findings suggest that MI may be considered for selected patients with chronic nonspecific low back pain who have low treatment motivation, poor exercise adherence, ambivalence toward behavior change, fear-avoidance beliefs, maladaptive coping, or difficulty engaging in active self-management. Given the limited and inconsistent evidence, MI should be regarded as an optional adjunct within multimodal rehabilitation rather than an essential component of routine treatment. Therefore, integrating motivational interviewing into multimodal treatment may be considered for selected patients with chronic nonspecific low back pain, while its advantage over standard care for subacute low back pain remains unclear (Level II evidence, Grade C recommendation).

#### Recommendation

Mindfulness meditation may be considered as a weakly recommended mind–body intervention for selected patients with chronic nonspecific low back pain, particularly those with pain interference, psychological distress, maladaptive pain coping, sleep disturbance, reduced self-efficacy, or opioid-treated refractory pain.

Mindfulness meditation is an 8-week, group-based mind–body intervention that integrates mindfulness meditation, body awareness, gentle yoga, and daily home practice to promote present-moment awareness and nonjudgmental acceptance of pain-related physical and emotional experiences. Cherkin et al. conducted a randomized clinical trial involving 342 adults with chronic low back pain, comparing mindfulness meditation, cognitive behavioral therapy, and usual care. At 26 weeks, mindfulness meditation produced significantly greater clinically meaningful improvements in functional limitation and pain bothersomeness than usual care, with benefits sustained at 52 weeks. No significant differences were observed between mindfulness meditation and cognitive behavioral therapy.^20^ Paschali et al. further performed a systematic review and meta-analysis including 18 studies with 873 participants, showing that mindfulness-based interventions were associated with reduced pain intensity and consistent within-group improvements in patients with chronic low back pain. Adverse events were minimal, and no serious harms were reported.^21^ These findings suggest that mindfulness meditation may be particularly suitable for patients with pain interference, psychological distress, maladaptive pain coping, sleep disturbance, reduced self-efficacy, or preference for self-directed nonpharmacological pain-management strategies. Based on the available Level I/II evidence, its favorable safety profile, durable functional benefits, and alignment with biopsychosocial pain mechanisms, mindfulness meditation may be considered as a weakly recommended mind–body intervention for selected patients with chronic low back pain (Level I/II evidence, Grade B recommendation).

#### Recommendation

Core stabilization exercise or motor control training can be considered as a weak recommendation for patients with chronic nonspecific low back pain, particularly those with movement control impairment, impaired trunk muscle activation, reduced lumbar stability, or persistent functional disability.

Core stabilization exercise or motor control training is a physiotherapist-led, individualized intervention designed to improve deep trunk muscle activation, correct impaired spinal movement patterns, restore lumbar stability, and integrate these changes into functional activities. Luomajoki et al. conducted a systematic review and meta-analysis including 11 randomized controlled trials with 781 patients with chronic nonspecific low back pain. Compared with control or other interventions, motor control exercise produced greater improvements in activity limitation and pain intensity at the end of treatment, and the improvement in activity limitation was sustained up to 12 months. However, the long-term effect on pain was no longer statistically significant. Subgroup analysis suggested that patients screened and confirmed to have movement control impairment achieved greater short-term improvements in function and pain, whereas evidence from unselected populations was of very low quality.^22^ The 2021 clinical practice guideline from the Academy of Orthopedic Physical Therapy further provides a Grade B recommendation for motor control exercise or trunk mobility exercise in patients with chronic low back pain.^23^ These findings suggest that core stabilization or motor control training is particularly suitable for patients with movement control impairment, impaired trunk muscle activation, reduced lumbar stability, or persistent functional disability. Given its favorable safety profile, modest functional benefits, study heterogeneity, and uncertainty regarding long-term pain relief, core stabilization exercise or motor control training may be considered as a weakly recommended rehabilitation option for selected patients with chronic nonspecific low back pain when delivered by trained physical therapists using standardized movement-control assessment(Level I/II evidence, Grade B recommendation).

#### Recommendation

Sling exercise may be considered as a weakly recommended active rehabilitation option for selected patients with chronic low back pain, particularly those with impaired trunk control, reduced core stability, movement control deficits, or clinical signs of lumbar spinal instability.

Sling exercise therapy is an active rehabilitation approach that uses suspension ropes, slings, and sometimes elastic support to reduce body weight loading while creating an unstable training environment. It aims to facilitate pain-free activation of deep trunk stabilizers, improve proprioception, enhance neuromuscular coordination, and retrain movement control of the lumbar spine. Drummond et al. conducted a systematic review and meta-analysis including 12 studies with 631 participants with chronic low back pain. Compared with general exercise, sling exercise showed no significant additional benefit for pain; however, it produced greater improvements in pain and disability than motor control training or lumbar stabilization exercise, and was superior to no treatment for pain. It also showed potential benefits for muscle-related outcomes, including core muscle activation, strength, thickness, and activation timing. However, the certainty of evidence ranged from very low to moderate because of heterogeneity, small sample sizes, variable comparators, and methodological limitations.^24^ Kim et al. further conducted a randomized assessor-blind controlled trial in patients with chronic low back pain and clinical signs of lumbar spinal instability. Both traditional trunk stabilization exercise and sling exercise improved pain and disability after 12 weeks and at 3-month follow-up, but sling exercise produced significantly greater improvements in Numeric Pain Rating Scale and Oswestry Disability Index scores, with no treatment-related adverse events.^25^ These findings suggest that sling exercise may be particularly suitable for patients with impaired trunk control, delayed stabilizer activation, reduced trunk endurance, poor postural adjustment, or suspected lumbar instability. Given its potential functional benefits, favorable safety profile, and limited certainty of evidence, sling exercise may be considered as a weakly recommended active rehabilitation option for selected patients with chronic low back pain, particularly those with trunk control deficits or clinical signs of lumbar instability (Level I/II evidence, Grade B recommendation).

#### Recommendation

Proprioceptive neuromuscular facilitation (PNF) may be considered as a weakly recommended active rehabilitation option for selected patients with chronic low back pain, particularly those with impaired trunk proprioception, neuromuscular coordination deficits, reduced trunk endurance, balance impairment, or persistent functional disability.

PNF training is therapist-guided neuromuscular exercise centered on rhythmic stabilization, diagonal limb and pelvic movement patterns alongside contract-relax and dynamic reversal techniques, commonly delivered 3—7 sessions weekly over 4 to 6 weeks. Arcanjo et al. completed a systematic review and meta-analysis incorporating 16 eligible randomized controlled trials involving 722 patients with chronic low back pain. Compared with blank control interventions, PNF generated significant pain improvement (SMD = − 2.6, 95%CI − 4.2 to − 0.9, *n* = 174) and disability reduction (SMD = − 3.29, 95%CI − 5.3 to − 1.3, *n* = 144). When contrasted with core strengthening training, PNF achieved superior pain relief (MD = − 1.8, 95%CI − 2.2 to − 0.3, *n* = 177) and better functional disability improvement (MD = − 6.6, 95%CI − 9.3 to − 3.8, n = 113); PNF also outperformed conventional physical therapy including TENS, hot pack and ultrasound in pain reduction (SMD = − 2.75, 95%CI − 3.9 to − 1.6, *n* = 106).^26^ GRADE evaluation denoted low-to-moderate overall evidence quality due to substantial inter-study heterogeneity and methodological defects of included trials. Based on available pooled meta-analytic Level II evidence, evident pain and disability improvement benefits and acceptable clinical safety, PNF is applicable for suitable patients when implemented by certified therapists following standardized technical specifications. Therefore, PNF training is conditionally recommended for adult patients with chronic nonspecific low back pain complicated by proprioceptive and motor control deficits (Level I evidence, Grade B recommendation).

#### Recommendation

The McKenzie method is strongly recommended as an individualized exercise-based rehabilitation option for selected patients with chronic low back pain, particularly those demonstrating directional preference or centralization during mechanical assessment.

The McKenzie method is a physiotherapist-led, classification-based intervention that uses repeated movement testing to identify directional preference or centralization and provides individualized exercise-based rehabilitation with an emphasis on self-management and postural education. Hennemann et al. conducted a systematic review and meta-analysis including 5 randomized controlled trials with 743 patients with chronic low back pain and directional preference. The review found low- to moderate-certainty evidence that the McKenzie method delivered by credentialed therapists produced clinically important short-term pain reduction and intermediate-term disability improvement compared with other conservative interventions. Compared with other exercise approaches, it showed clinically important short-term benefits for both pain and disability; compared with minimal intervention, it showed clinically important intermediate-term improvements in pain and disability and long-term improvement in disability.^27^ Lam et al. further performed a meta-analysis including 17 studies and reported moderate- to high-quality evidence that the McKenzie method was superior to other rehabilitation interventions for reducing pain and disability in chronic low back pain, with small to moderate effect sizes.^28^ These findings suggest that the McKenzie method is particularly suitable for patients who demonstrate directional preference or centralization during mechanical assessment. Based on the available Level I evidence, clinically important benefits, favorable safety profile, and the need for standardized assessment by trained therapists, the McKenzie method is strongly recommended as an individualized exercise-based rehabilitation option for selected patients with chronic low back pain (Level I evidence, Grade A recommendation).

#### Recommendation

Low-load blood flow restriction training (LL-BFRT) may be considered as a weakly recommended adjunctive strengthening strategy for selected patients with chronic nonspecific low back pain, particularly those with impaired core muscle strength or endurance who cannot tolerate high-load resistance training.

LL-BFRT is a supervised resistance training with limb cuff blood flow occlusion, delivered at 30% 1-repetition maximum (1RM) load and 70% arterial occlusion pressure, containing deep squat, pull-down, bench-press and seated crunch movements. Liu et al. performed a randomized controlled trial including 26 CNLBP male athletes randomly split into LL-BFRT (*n* = 13) and conventional heavy-load resistance training (HL-RT, *n* = 13), receiving 4 weekly training sessions for 4 weeks. At post-intervention, LL-BFRT yielded highly significant reductions in VAS and ODI scores (*P* < 0.001, ES = 1.44–1.84), superior to statistically moderate improvements in HL-RT (*P* < 0.05, ES = 0.70–0.71). Both groups significantly elevated isometric core extensor endurance (LL-BFRT: *P* = 0.016, ES = 0.78; HL-RT: *P* = 0.011, ES = 0.83); LL-BFRT improved extensor PT/BW at 120°/s (*P* = 0.045, ES = 0.62) while HL-RT gained strength at 30°/s (*P* = 0.013, ES = 0.81), with equivalent overall core strength enhancement. LL-BFRT exerts analgesic effects via endogenous pain modulation and reduces spinal loading risks associated with high-intensity training.^29^ Without supporting pooled meta-analytic data, available evidence originates from a single RCT. Based on Level II trial evidence, reliable pain and functional benefits and favorable safety features, LL-BFRT is suitable for eligible athletes when implemented per standardized protocol by qualified therapists. Therefore, LL-BFRT is conditionally recommended for male collegiate athletes with CNLBP unable to bear heavy-load exercise (Level II evidence, Grade B recommendation).

#### Recommendation

Aquatic exercise is strongly recommended as an active exercise-based rehabilitation option for selected patients with chronic low back pain, particularly those who have difficulty tolerating land-based exercise.

Aquatic exercise is a structured active rehabilitation approach performed in a water-based environment, using buoyancy, hydrostatic pressure, water resistance, and thermal effects to facilitate movement, reduce spinal loading, improve muscle relaxation, and enable progressive exercise with less pain provocation. It may include walking, stretching, trunk mobility training, strengthening, balance exercise, aerobic activity, and functional movement practice in water. Ma et al. conducted a systematic review and meta-analysis including 13 randomized controlled trials with 597 patients with chronic low back pain. Compared with land-based or non-aquatic comparators, aquatic physical therapy significantly reduced pain intensity, improved physical and mental components of quality of life, and decreased disability measured by the Roland-Morris Disability Questionnaire and Oswestry Disability Index. However, no significant benefit was found for pain at rest, and the certainty of evidence was rated very low to low because of risk of bias and methodological limitations.^30^ The 2021 clinical practice guideline from the Academy of Orthopedic Physical Therapy further recommends exercise interventions for chronic low back pain with Grade A strength, including trunk strengthening, endurance exercise, multimodal exercise, aerobic exercise, aquatic exercise, and general exercise.^31^ These findings suggest that aquatic exercise is particularly suitable for patients who have difficulty tolerating land-based exercise because of pain, obesity, deconditioning, fear of movement, reduced balance, joint comorbidity, or poor baseline exercise capacity. Given its favorable safety profile, reduced mechanical loading, and guideline-supported role as an active exercise option, aquatic exercise is strongly recommended for selected patients with chronic low back pain, especially those who cannot tolerate conventional land-based exercise (Level I evidence, Grade A recommendation).

#### Recommendation

Pilates training may be considered as a weakly recommended mind–body exercise-based rehabilitation option for selected patients with chronic nonspecific low back pain, particularly those with impaired trunk control, reduced core endurance, kinesiophobia, pain catastrophizing, or persistent functional disability.

Pilates training is a mind–body exercise intervention delivered by physiotherapists, focusing on core activation, neutral spine control, breathing coordination, and movement precision. It aims to improve neuromuscular control of deep trunk muscles (e.g., transversus abdominis, multifidus) and restore lumbar stability. Patti et al. conducted a systematic review (36 RCTs, 2030 participants) confirming that Pilates significantly improved pain and disability compared with no exercise, but showed no significant difference compared with non‑specific exercise.^32^ Wong et al. conducted a systematic review (11 RCTs) showing that Pilates had a small benefit over general exercise for pain (low‑certainty evidence), but no significant difference compared with direction‑specific or spinal stabilization exercise.^33^ Li et al. conducted a network meta‑analysis (75 RCTs, 5 254 patients) showing that Pilates was superior to conventional rehabilitation and no intervention for pain, and superior to no intervention for function.^34^ These findings suggest that Pilates is particularly suitable for patients with impaired trunk control, reduced core endurance, kinesiophobia, pain catastrophizing, or persistent functional disability. Based on low‑ to moderate‑quality evidence, considerable heterogeneity, non‑inferior but not superior results compared with other active exercises, and a favorable safety profile, Pilates is recommended for appropriately selected patients when delivered by trained physiotherapists after standardized assessment. Therefore, for selected patients with chronic nonspecific low back pain, Pilates training may be considered as a weakly recommended mind–body exercise‑based rehabilitation option (Level I evidence, Grade B recommendation).

#### Recommendation

Baduanjin or Qigong is strongly recommended as a low-intensity mind–body exercise option for selected patients with chronic nonspecific low back pain, particularly those who prefer home-based exercise or have reduced physical capacity, poor exercise adherence, psychological stress, or difficulty tolerating conventional exercise.

Baduanjin is a standardized traditional Chinese mind-body qigong exercise consisting of eight coordinated slow movements combining posture control, rhythmic breathing and neuromuscular regulation, implemented at moderate intensity without high spinal loading, with common intervention specifications of 30 min per session, 3–5 times weekly over 4–12 weeks under standardized coaching guidance.^35,36^ Yang et al. conducted a single-center randomized controlled trial involving 60 patients with chronic nonspecific low back pain, comparing a 4-week supervised Baduanjin program with ordinary walking. Compared with walking, Baduanjin produced greater reductions in pain intensity and disability, as reflected by improvements in NPRS and ODI scores. Surface electromyography also showed improved erector spinae activation and flexion–relaxation responses in the Baduanjin group.^37^ Yu et al. and Zou et al. further performed systematic reviews and meta-analyses of randomized controlled trials, showing that Baduanjin or Qigong significantly reduced pain and disability in patients with chronic nonspecific low back pain.^4,35,37^ Additional pooled evidence on traditional Chinese exercises also supported beneficial effects on pain relief, trunk endurance, and functional recovery.^38^ These findings suggest that Baduanjin may improve chronic low back pain through enhanced paraspinal muscle function, improved movement control, autonomic regulation, and reduced pain-related stress, with few adverse events reported across trials. However, the evidence is limited by heterogeneity in training duration, exercise protocols, supervision intensity, and participant characteristics. Given its favorable safety profile, low physical burden, and consistent short-term benefits for pain and function, Baduanjin or Qigong may be considered for selected patients with chronic nonspecific low back pain, particularly those who prefer home-based low-intensity exercise, have reduced physical capacity, poor exercise adherence, psychological stress, or difficulty tolerating conventional exercise^4,35,37–39^ (Level I evidence, Grade B recommendation).

#### Recommendation

Tai Chi is strongly recommended as an initial non-pharmacological exercise option for selected adults with chronic nonspecific low back pain, particularly when a low-impact mind-body program aligns with patient preference and access to supervised instruction.

Tai Chi is a traditional mind–body exercise that integrates slow controlled movements, postural alignment, diaphragmatic breathing, balance training, and mindful attention. It may improve chronic low back pain through enhanced trunk and lower-limb strength, flexibility, balance, proprioception, movement confidence, relaxation, and pain coping. Li et al. conducted a systematic review and meta-analysis including 14 randomized controlled trials with 960 adults with chronic low back pain. Compared with control interventions, Tai Chi significantly reduced pain intensity and improved disability. The certainty of evidence was moderate for pain reduction but very low for disability because of heterogeneity, small sample sizes, and methodological limitations.^40^ Subgroup analyses suggested that Chen-style Tai Chi and training more than three times per week may be associated with greater pain reduction, although the optimal style, dose, and duration remain uncertain. The American College of Physicians guideline recommends non-pharmacological treatments, including Tai Chi, as initial management options for adults with chronic low back pain before pharmacological therapy.^41^ These findings suggest that Tai Chi is particularly suitable for patients who prefer low-impact mind–body exercise, have a fear of movement, reduced balance, deconditioning, psychological stress, or poor tolerance of higher-intensity land-based exercise. Given its favorable safety profile, accessibility, and guideline-supported role as an initial non-pharmacological intervention, Tai Chi is strongly recommended for selected adults with chronic nonspecific or chronic primary low back pain when delivered by trained instructors and adapted to individual mobility and comorbidity status (Level I evidence, Grade A recommendation).

#### Recommendation

Yoga is weakly recommended as an initial non-pharmacological exercise option for selected adults with chronic nonspecific low back pain, when a low-impact mind-body program aligns with patient preference and access to appropriate instruction.

Yoga is a low-impact mind–body exercise that integrates physical postures, stretching, breathing techniques, relaxation, and, in some programs, meditation or lifestyle education. It may improve chronic low back pain through enhanced flexibility, trunk and hip muscle endurance, postural control, body awareness, relaxation, and movement confidence. The systematic review supporting the American College of Physicians guideline found that yoga produced small to moderate improvements in pain and function in patients with chronic low back pain, particularly compared with education, usual care, or wait-list control.^42^ Anheyer et al. further conducted a systematic review and meta-analysis including 30 articles from 27 randomized controlled trials with 2 702 participants. Compared with passive control interventions, yoga improved short-term pain intensity, pain-related disability, mental health, and physical functioning. Improvements in pain, disability, and physical functioning were generally sustained at longer-term follow-up, whereas benefits for mental health were less consistent. However, yoga was not clearly superior to other active exercise interventions.^43^ Adverse events were mostly nonserious and commonly related to transient musculoskeletal discomfort. These findings suggest that yoga may be suitable for selected adults with chronic nonspecific or chronic primary low back pain who prefer a gentle mind–body approach and have access to appropriately trained instructors. Given its modest benefits, favorable safety profile, and lack of clear superiority over other active exercise programs, yoga is weakly recommended as an initial non-pharmacological exercise option when adapted to pain irritability, physical capacity, balance, and comorbidities, and when provocative postures or excessive lumbar flexion, extension, or loading are avoided (Level I evidence, Grade B recommendation).

#### Recommendation

Vojta therapy may be weakly recommended as an adjunct, therapist-guided neuromotor rehabilitation option for selected patients with chronic nonspecific low back pain, particularly those with marked functional limitation and suspected deficits in trunk postural control, when it is delivered as part of a multimodal rehabilitation program and tailored to patient tolerance.

Vojta therapy is a neurophysiological physical intervention relying on targeted compression of specific reflex activation zones to induce spontaneous creeping and rolling reflexes, improving automatic trunk and spinal postural control; it is delivered in 20-minute single sessions alongside conventional hydrotherapy, massage, electrotherapy and routine rehabilitation programs. Iosub et al. completed a single-center randomized controlled trial including 148 eligible chronic low back pain participants, randomly allocated into combined Vojta plus standard care group (*n* = 82) and standard-care-only control group (*n* = 66), with a 10-day total intervention cycle. After treatment, both groups achieved significant pain relief on VAS, while the Vojta combined group obtained statistically superior improvements in ODI (*P* < 0.001), Roland-Morris Disability scores (*P* < 0.001), Hamilton depression scale and Rosenberg self-esteem scale compared with the control cohort.^44^ Correlation analysis confirmed reductions in depression were accompanied by improved self-esteem and lower disability degree, and no adverse treatment events were recorded throughout the trial. Currently no published pooled meta-analyses synthesizing multiple RCTs concerning Vojta for chronic low back pain are available. Based on available single-center RCT-derived Level II evidence, favorable safety profile and verified combined treatment benefits on physical and psychological outcomes, Vojta therapy should be administered by trained physiotherapists following standardized reflex stimulation protocols. Therefore, Vojta therapy is conditionally recommended as supplementary intervention for chronic low back pain patients with postural dysfunction and mild depressive symptoms (Level Ⅱ evidence, Grade B recommendation).

#### Recommendation

Feldenkrais method may be weakly recommended as an active, movement re-education–based rehabilitation option for selected patients with chronic nonspecific low back pain, particularly those with marked functional limitation or impaired body awareness, when delivered by trained instructors and integrated into a structured rehabilitation program.

Feldenkrais therapy is a sensory-awareness-based movement intervention focusing on gentle guided motion to improve body perception and relieve fear-avoidance behavior, while DNS relies on developmental movement patterns to strengthen core and optimize spinal alignment; the combined program is implemented 3 sessions weekly for 8 weeks, each session combining standard DNS training plus supplementary Feldenkrais guided movement practice. Saki and Ziya completed a single-center randomized controlled trial recruiting 30 older women aged 60–80 years, equally allocated into DNS-only group (*n* = 15) and combined intervention group (*n* = 15). After 8-week supervised training, both groups achieved significant within-group improvements in VAS pain, TSK kinesiophobia score and lumbar flexion/extension ROM, whereas the combined group produced statistically superior clinical gains across all indicators (VAS *P* = 0.010, TSK *P* < 0.001), lumbar flexion (*P* = 0.007), lumbar extension (*P* < 0.001), with small-to-medium between-group effect sizes.^45^ No adverse events were observed throughout the trial, yet available evidence is limited to this single RCT without relevant published meta-analysis data. Based on available Level II evidence, acceptable safety profile and verified synergistic clinical benefits targeting pain, movement phobia and spinal mobility, the combined program should be delivered by qualified rehabilitation therapists following standardized training specifications. Therefore, Feldenkrais combined with DNS exercise is conditionally recommended for elderly females with chronic nonspecific low back pain and evident kinesiophobia and restricted lumbar mobility (Level Ⅱ evidence, Grade B recommendation).

#### Recommendation

Connective tissue massage (CTM) may be weakly recommended as an adjunct soft-tissue manual therapy option for selected patients with chronic nonspecific low back pain, particularly those with restricted lumbar mobility and reduced health-related well-being.

CTM is a fascial-focused manual therapy relying on hooked pulling manipulation targeting subcutaneous connective tissue to regulate cutaneous-visceral reflex and autonomic nervous activity; classical massage mainly adopts stroking and kneading techniques to ease muscular tension and promote endogenous analgesic secretion. Both massage protocols are applied alongside hot compress and matched lumbar exercise, scheduled five sessions per week for a total 20 treatments.^46,47^ Er et al. implemented single-center RCT recruiting 70 eligible patients randomly divided into CT group (*n* = 35) and CM group (*n* = 35); another three-arm RCT by Dal et al enrolled 30 subjects into CTM, CM and routine physiotherapy subgroups respectively. After 4-week intervention and prolonged 6-week follow-up observation, both massage modalities generated statistically significant reductions in VAS pain, ODI disability index and PSQI sleep disorder plus improved SF-36 life quality (*P* < 0.05) for all within-group comparisons. CM achieved faster early pain relief at the second treatment week (*P* = 0.04), while CTM presented more prominent overall improvement in multiple quality-of-life domains and peripheral skin temperature related autonomic indexes; between-group overall efficacy was comparable in pain and disability improvement (*P* > 0.05). No adverse treatment events were reported across trials, while no published pooled meta-analysis covering these two massage modalities is available presently. Based on existing independent RCT-derived Level II evidence, favorable safety profile and confirmed persistent therapeutic benefits on pain, function and autonomic regulation, CTM and CM should be delivered by certified massage therapists following standardized manipulation specifications. Therefore, connective tissue massage and classical massage are conditionally recommended as optional manual rehabilitation interventions for patients with chronic mechanical low back pain (Level Ⅱ evidence, Grade B recommendation).

#### Recommendation

Spinal manipulative therapy may be weakly recommended as an adjunct, manual therapy option for selected patients with chronic nonspecific low back pain, particularly when delivered as part of a treatment package including exercise.

Spinal manipulative therapy is a clinician-led, hands-on intervention delivered by physiotherapists, chiropractors, or physicians, including high-velocity manipulation and low-velocity mobilization. It aims to improve joint mobility, reduce pain, and restore spinal function. Rubinstein et al. conducted a systematic review and meta-analysis including 47 randomized controlled trials with 9 211 patients with chronic low back pain. Moderate-quality evidence showed that spinal manipulative therapy had similar effects to recommended therapies for short-term pain relief and slightly better effects for short-term function, while providing small clinical benefits compared with non-recommended therapies.^48^ Paige et al. further conducted a systematic review including 26 randomized controlled trials in acute low back pain and found moderate-quality evidence that spinal manipulative therapy was associated with modest improvements in pain and function for up to 6 weeks.^49^ The VA/DoD clinical practice guideline also recommends spinal mobilization as part of a multimodal treatment program.^50^ These findings suggest that spinal manipulative therapy may be suitable for selected patients with mechanical or nonspecific low back pain without red flags or serious structural pathology. Given its modest benefits, favorable safety profile when delivered by trained clinicians, and comparable effects to other recommended conservative therapies, spinal manipulative therapy may be weakly recommended as an adjunct manual therapy option, particularly when combined with exercise and education rather than used as a stand-alone treatment (Level I evidence, Grade B recommendation).

#### Recommendation

Visceral manipulation (VM) may be weakly recommended as an adjunct manual therapy option for selected patients with chronic nonspecific low back pain, particularly those with coexisting visceral dysfunction or clinical features suggestive of a visceral contribution.

Visceral manipulation (VM) is a specialized osteopathic manual intervention targeting abdominal visceral fascia and sphincter tissues via localized gentle deep manipulation, which aims to improve visceral fascial mobility, cut down abnormal visceral nociceptive afferent input toward lumbar spinal segments and further ameliorate lumbosacral biomechanical restriction; the standard intervention scheme consists of one session weekly for five consecutive weeks, each treatment allocates 10 min of targeted visceral manipulation after 40 min standardized conventional physiotherapy. Villalta Santos et al. conducted a double-blind randomized controlled trial recruiting 20 eligible participants equally split into combined treatment group and placebo visceral manipulation plus conventional physiotherapy control group. After five-week intervention and one-week follow-up observation, no intergroup statistically significant difference was detected in VAS pain index, whereas the combined VM group achieved obviously superior improvement in lumbar spinal mobility measured by Schober test and patient-specific functional scale scores (*P* < 0.05); conventional rehabilitation alone obtained equal pain relief effect but failed to improve localized lumbar mobility and individual-specific daily function.^51^ No severe adverse events were recorded throughout the trial, yet presently there is a lack of relevant large-sample RCTs and pooled meta-analysis evidence concerning visceral manipulation for low back pain. Based on existing single randomized controlled trial Level II evidence and acceptable safety performance, visceral manipulation can be applied as supplementary adjuvant treatment together with regular physiotherapy under professional osteopath guidance for CNLBP patients accompanied with various abdominal and pelvic visceral dysfunction. (Level Ⅱ evidence, Grade B recommendation).

#### Recommendation

Myofascial release may be weakly recommended as an adjunct soft tissue manual therapy option for selected patients with chronic nonspecific low back pain, particularly those with marked functional limitation.

Myofascial release is a soft tissue manual therapy targeting the fascia, muscles, and related connective tissues of the lumbopelvic region. It typically involves sustained low-load manual pressure, stretching, and tissue mobilization to reduce myofascial tension, improve tissue mobility, modulate pain, and restore functional movement. Chen et al. conducted a systematic review and meta-analysis including 8 randomized controlled trials with 386 patients with low back pain. Myofascial release significantly reduced disability compared with control interventions, but showed no significant effects on pain intensity, quality of life, or lumbar range of motion.^52^ Wu et al. further included 8 randomized controlled trials with 375 patients with chronic low back pain and found significant improvements in pain and physical function, but no clear benefits for quality of life, balance, pain pressure threshold, trunk mobility, or mental health.^53^ Ozóg et al. reviewed 6 randomized controlled trials of isolated myofascial release in adults with chronic low back pain and reported improvements in pain intensity, range of motion, functional disability, fear-avoidance beliefs, and paraspinal muscle activity after repeated treatment sessions, whereas evidence for single-session interventions was inconsistent.^54^ Overall, the evidence is limited by small sample sizes, heterogeneous treatment protocols, variable comparators, and insufficient long-term follow-up. These findings suggest that myofascial release may be most suitable for selected patients with chronic nonspecific low back pain who have soft tissue sensitivity, restricted mobility, or marked functional limitation. Given its potential short-term functional benefits but uncertain broader and long-term effects, myofascial release should not be used as a stand-alone curative treatment, but may be considered as an adjunct to education, active exercise, and self-management when delivered by trained clinicians and continued only if meaningful functional improvement is observed (Level I evidence, Grade C recommendation).

#### Recommendation

Tuina may be weakly recommended as an active manual therapy–based rehabilitation option for selected patients with chronic nonspecific low back pain, particularly those who have difficulty tolerating land-based or other active exercise programs.

Tuina is a traditional Chinese manual therapy that uses techniques such as pressing, kneading, rolling, rubbing, stretching, passive mobilization, and acupoint stimulation to reduce pain, relieve soft-tissue tension, improve local circulation, and restore lumbar mobility. Yang et al. conducted a systematic review and meta-analysis including 15 randomized controlled trials with 1 390 patients with chronic nonspecific low back pain. Compared with control interventions, Tuina significantly improved pain intensity and physical function, with standardized mean differences of − 0.82 and − 0.91, respectively. However, no significant improvement was observed in quality of life, and the certainty of evidence was rated low because of methodological limitations, heterogeneity, and imprecision.^55^ Ma et al. further conducted a randomized controlled trial involving 204 patients, comparing Tuina, physiotherapy, and combined Tuina plus physiotherapy. All groups showed improvements in pain and disability after treatment and at 20-week follow-up, but no significant between-group differences were found.^56^ Another randomized controlled trial similarly reported that Tuina produced improvements in pain, disability, and physical quality of life comparable to physical therapy or combined treatment, with high patient satisfaction and a favorable safety profile.^57^ These findings suggest that Tuina may be useful as a non-pharmacological manual therapy option for selected patients who prefer therapist-assisted treatment, have difficulty tolerating active exercise, or require a bridge toward active rehabilitation. Given the limited certainty of evidence and lack of clear superiority over physiotherapy, Tuina should be integrated with education, self-management, and progressive activity rather than used as a stand-alone definitive treatment. Therefore, Tuina may be weakly recommended for selected patients with chronic nonspecific low back pain (Level I evidence, Grade C recommendation).

#### Recommendation

Instrument-assisted soft tissue mobilization (IASTM) may be considered as a weakly recommended adjunct for chronic low back pain.

IASTM is a manual therapy technique that uses rigid handheld instruments to address soft tissue restriction, fascial stiffness, and myofascial tightness. It is intended to improve tissue mobility, increase local circulation, reduce soft tissue tension, and facilitate subsequent stretching or active rehabilitation. Gul et al. conducted a randomized controlled trial including 50 patients with chronic mechanical low back pain. Participants received either manual myofascial release or IASTM for 9 sessions over 3 weeks, with both groups also receiving moist heat and stretching. Pain, lumbar range of motion, and Oswestry Disability Index scores improved significantly within both groups, but between-group differences were generally not significant, suggesting that IASTM may be comparable to manual soft tissue mobilization rather than clearly superior.^58^ Yildirim et al. conducted a double-blind randomized controlled trial involving 60 patients with lumbar disc herniation. IASTM combined with conventional physiotherapy produced greater improvements in lumbar range of motion and disability than conventional physiotherapy alone, but no significant between-group differences were observed for pain intensity or most quality-of-life outcomes.^59^ No important harms were reported. These findings suggest that IASTM may be useful as an adjunctive soft tissue intervention for selected patients with chronic low back pain associated with soft tissue restriction, reduced lumbar mobility, or functional limitation. Given the limited evidence base, small sample sizes, short follow-up, and uncertain long-term effects, IASTM should not be used as a stand-alone treatment, but may be considered as an adjunct to education, exercise therapy, stretching, and progressive functional rehabilitation (Level I/II evidence, Grade C recommendation).

#### Recommendation

Moxibustion may be recommended as an adjunct non-pharmacological, heat-based therapy option for selected patients with chronic nonspecific low back pain, particularly when integrated into a multimodal rehabilitation package.

Moxibustion is a traditional Chinese medicine therapy with a history of thousands of years, involving the ignition of moxa velvet or moxa sticks to burn or fumigate specific acupoints, leveraging heat and medicinal properties to prevent and treat diseases. Rooted in meridian theory, it works by warming meridians, promoting qi and blood circulation, and dispelling cold-dampness pathogens. Yao et al. conducted a meta-analysis including 21 randomized controlled trials with 2 198 patients to evaluate thunder-fire moxibustion for low back pain. Compared with control interventions, thunder-fire moxibustion significantly reduced pain intensity and disability, improved Japanese Orthopedic Association scores, and produced short-term improvements in quality of life, particularly in the bodily pain domain. The most frequently used acupoints were BL23, Ashi points, and GV3. Safety was generally favorable, with only rare, mild, and self-limiting skin reactions reported.^60^ Chen et al. further conducted a meta-analysis of 10 randomized controlled trials with 987 patients evaluating general moxibustion for chronic low back pain. The results suggested that moxibustion reduced pain and improved function, particularly when combined with other treatments; however, the certainty of evidence was very low to low because of methodological limitations, heterogeneity, and imprecision.^61^ No clear advantage was observed over core stability training alone. These findings suggest that moxibustion may be considered as an adjunct heat-based non-pharmacological therapy for selected patients with chronic nonspecific low back pain, particularly those who prefer traditional Chinese medicine approaches or tolerate thermal therapy well. Given its favorable safety profile but limited certainty of evidence, moxibustion may be weakly recommended as part of a multimodal rehabilitation package rather than as a stand-alone treatment (Level I evidence, Grade B recommendation).

#### Recommendation

Acupuncture is strongly recommended as a first-line non-pharmacological option for chronic nonspecific low back pain.

Acupuncture is a needle-based non-pharmacological intervention that may produce analgesic effects through activation of descending inhibitory pathways, modulation of pain-related brain activity, and local effects on myofascial pain generators. It can be delivered as individualized treatment based on traditional Chinese medicine diagnosis or as a standardized protocol. Cherkin et al. conducted a randomized controlled trial involving 638 adults with chronic mechanical low back pain, comparing individualized acupuncture, standardized acupuncture, simulated acupuncture, and usual care. After 10 treatments over 7 weeks, all three acupuncture groups showed greater improvement in back-related dysfunction than usual care at 8 weeks, with mean Roland-Morris Disability Questionnaire improvements of 4.4, 4.5, and 4.4 points, respectively, compared with 2.1 points in the usual care group. Approximately 60% of patients receiving acupuncture achieved clinically meaningful functional improvement, compared with 39% receiving usual care, and the benefits were sustained at 52 weeks. No significant differences were observed between real and simulated acupuncture, suggesting that contextual or non-specific neurophysiological mechanisms may contribute substantially to the observed effects. Adverse events were mild and uncommon, and medication use decreased more in the acupuncture groups than in usual care.^62^ Cashin et al. further developed a Cochrane overview protocol to synthesize evidence on non-pharmacological and non-surgical treatments for low back pain, including acupuncture as a key non-drug intervention.^63^ These findings suggest that acupuncture is particularly suitable for patients with chronic nonspecific low back pain who prefer non-pharmacological analgesia or wish to reduce medication use. Based on the available Level I evidence, its favorable safety profile, sustained functional benefits, and role as a non-drug treatment option, acupuncture is strongly recommended as a first-line non-pharmacological treatment for selected patients with chronic nonspecific low back pain (Level I evidence, Grade A recommendation).

Recommendation 5.1.27: Dry needling may be weakly recommended as an adjunct intervention for selected patients with chronic low back pain, particularly those with myofascial trigger points.

Dry needling is a minimally invasive intervention in which a thin filiform needle is inserted into active or latent myofascial trigger points to reduce trigger point irritability, modulate peripheral and central sensitization, decrease muscle tone, and improve pain-related movement tolerance. Liu et al. conducted a systematic review and meta-analysis of 11 randomized controlled trials involving 802 patients with low back pain associated with myofascial trigger points. Compared with other treatments, dry needling produced significant post-intervention reductions in pain intensity and functional disability, although follow-up effects remained unclear and the evidence was limited by heterogeneity, variable treatment protocols, small sample sizes, and risk-of-bias concerns.^64^ Lara-Palomo et al. further performed a systematic review and meta-analysis including 8 randomized controlled trials with 414 patients with chronic low back pain. Dry needling, particularly when combined with other physical therapy interventions, was more effective than comparator treatments in reducing pain intensity immediately after treatment and at short-term follow-up. However, there was no clear evidence of meaningful improvement in disability either immediately after treatment or during short-term follow-up.^65^ These findings suggest that dry needling may be most suitable for selected patients with identifiable myofascial trigger points, localized muscle tenderness, or pain-related movement limitation. Given its short-term analgesic benefit, uncertain functional and long-term effects, and protocol heterogeneity, dry needling should not be used as a stand-alone treatment, but may be considered as an adjunct to exercise therapy, education, manual therapy, and active rehabilitation when delivered by appropriately trained clinicians. Therefore, dry needling is weakly recommended as an adjunct intervention for selected patients with chronic low back pain, particularly those with myofascial trigger points (Level I evidence, Grade C recommendation).

Recommendation 5.1.28: Transcutaneous electrical nerve stimulation (TENS) may be weakly recommended for low back pain; a short trial may be considered as an adjunct for selected patients.

TENS is a non-invasive electrical stimulation therapy that delivers low-voltage impulses through surface electrodes placed over or near the painful lumbar region. It is proposed to relieve pain through segmental inhibitory mechanisms and activation of descending pain-modulatory pathways. Verville et al. conducted a systematic review and meta-analysis to inform the World Health Organization guideline on chronic primary low back pain, including 17 randomized controlled trials with 1 027 adults. Compared with sham stimulation, TENS produced a statistically significant but small immediate-term reduction in pain at approximately 2 weeks, with a mean difference of − 0.90 points on a 0–10 pain scale. This effect did not reach the prespecified threshold for a minimally important clinical difference. The review found little or no difference in function, health-related quality of life, depression, medication use, or adverse events, and no evidence was available for intermediate- or long-term outcomes. The certainty of evidence was rated very low because of risk of bias, inconsistency, indirectness, and imprecision.^66^ Earlier Cochrane evidence also found limited and inconsistent support for TENS as an isolated treatment.^67^ In the multicenter LOMBOTENS trial involving 236 patients with chronic low back pain with or without radicular pain, active TENS did not provide significant functional benefit compared with sham TENS at 6 weeks or 3 months.^68^ These findings suggest that TENS may provide short-term symptomatic relief in some patients, but should not replace exercise therapy, education, or biopsychosocial management. Given its accessibility, non-invasive nature, and generally favorable safety profile, TENS may be considered as a time-limited adjunctive option for selected patients with low back pain, particularly when used to facilitate participation in active rehabilitation and discontinued if no clinically meaningful benefit is observed after a short trial (Level I evidence, Grade B recommendation).

Recommendation 5.1.29: Transcranial direct current stimulation (tDCS) may be weakly recommended as an adjunct neuromodulation option for selected patients with chronic nonspecific low back pain, particularly when combined with structured exercise therapy to achieve modest short-term pain reduction, given low-to-moderate certainty and inconsistent functional benefits.

tDCS is a non-invasive neuromodulation technique that delivers weak direct currents through scalp electrodes to modulate cortical excitability within pain-processing networks. Luedtke et al. conducted a double-blind randomized controlled trial including 135 patients with chronic low back pain and found that five daily sessions of 20-min, 2 mA anodal tDCS over the left motor cortex did not significantly improve pain or disability compared with sham stimulation, with a mean difference of 1 mm on the pain visual analogue scale and 1 point on the ODI.^69^ Alwardat et al. further performed a systematic review and meta-analysis including 9 randomized controlled trials with 411 participants and found no significant effect of tDCS on pain or disability.^70^ Similarly, Armbrust et al. conducted a randomized trial involving 72 patients and showed that adding tDCS to osteopathic manipulative treatment provided no additional benefit over osteopathic manipulative treatment alone for pain or functional outcomes.^71^ These findings indicate that current evidence does not support tDCS as a stand-alone or routine treatment for chronic nonspecific low back pain. However, given its favorable safety profile and the possibility of modest short-term pain relief when combined with structured exercise in selected patients, tDCS may be considered as a weakly recommended adjunct neuromodulation option for patients with chronic nonspecific low back pain who respond insufficiently to conventional rehabilitation, particularly when used alongside active exercise-based therapy rather than as an isolated intervention (Level I/II evidence, Grade B recommendation).

Recommendation 5.1.30: Peripheral nerve stimulation (PNS) may be considered as a weakly recommended neuromodulation option for selected patients with refractory chronic low back pain.

PNS is a minimally invasive, non-ablative neuromodulation technique that uses percutaneously placed fine-wire leads near target peripheral nerves to modulate afferent input and pain-processing pathways. It is mainly considered for selected patients with refractory chronic axial low back pain without dominant radicular leg pain after failure of conventional non-surgical treatments, including medications, physical therapy, injections, or radiofrequency procedures. Gilmore et al. conducted a prospective multicenter case series including 74 patients with chronic axial low back pain, with a mean pain duration of 16.0 years and a mean of 5.3 failed prior treatments. Patients received 60-day percutaneous PNS targeting the lumbar medial branch nerves. At 2 months, 73% of patients achieved at least 30% reduction in average pain intensity, with a mean pain reduction of 58%; 73% achieved at least 10-point improvement in Oswestry Disability Index; and 73% achieved at least 30% reduction in pain interference. Benefits were partly sustained after lead removal, with 58% maintaining at least 30% pain improvement, 50% maintaining at least 10-point ODI improvement, and 56% maintaining at least 30% reduction in pain interference at 14 months. Among patients using opioids at baseline, 75% reduced opioid consumption, and no serious or unanticipated study-related adverse events were reported.^72^ These findings suggest that PNS may provide durable improvements in pain, disability, and pain interference in carefully selected patients with refractory chronic axial low back pain. However, the current evidence is limited by the absence of randomized or sham-controlled trials. Therefore, PNS may be considered as a weakly recommended neuromodulation option for selected patients with refractory chronic low back pain after failure of conventional treatments (Level II evidence, Grade C recommendation).

#### Recommendation

Intradiscal orthobiological injections (e.g., platelet-rich plasma) can be considered as a weak recommendation for patients with ankylosing spondylitis–related chronic low-back pain.

PRP consists of an increased concentration of autologous platelets suspended in a small amount of plasma, releasing growth factors that promote tissue repair and reduce inflammation.^73^ A recent meta-analysis pooling five RCTs reported a ≥ 3-point decrease in the numeric rating scale (NRS) at six months, with benefits persisting for 12 months.^74^ A prospective randomized controlled trial further demonstrated that ultrasound-guided sacroiliac PRP injection outperforms corticosteroid in both pain relief and functional improvement.^73^Based on the available Level Ⅰ evidence, its favorable safety profile, sustained pain reduction benefits, and potential for functional improvement in refractory cases, intradiscal Orthobiological therapy is recommended for appropriately selected patients when delivered by trained clinicians using standardized injection protocols and imaging guidance. Therefore, PRP injection is weakly recommended for selected patients with chronic discogenic low back pain with prominent degenerative disc disease, failed conservative management, or structural contributors to disability refractory to conventional interventions (Level I evidence, Grade B recommendation).

#### Recommendation

Intradiscal mesenchymal stem cell injection may be considered as a weakly recommended investigational treatment option for carefully selected patients with discogenic chronic low back pain, preferably within ethically approved or registered clinical study settings.

Intradiscal mesenchymal stem cell injection is an investigational biological therapy that involves injecting culture-expanded mesenchymal stromal or stem cells into a degenerated intervertebral disc to modulate local inflammation and potentially support extracellular matrix repair. Available evidence suggests possible benefits for pain relief and functional improvement in selected patients with discogenic chronic low back pain, but superiority over placebo has not been clearly established and long-term safety and efficacy remain uncertain. A meta-analysis including 21 relevant studies suggested that intradiscal mesenchymal stem cell injection may improve clinical outcomes in discogenic low back pain.^75^ However, in a randomized placebo-controlled trial involving 114 patients aged 18–60 years, a single intradiscal injection of 20 million allogeneic bone marrow-derived mesenchymal stem cells did not produce superior improvements in Visual Analog Scale or Oswestry Disability Index scores compared with placebo at 24 months, although the safety profile was favorable.^76^ A small case series of 5 patients who had failed conservative treatment reported meaningful pain reduction lasting up to 24 weeks in 3 patients, with associated improvement in disability.^77^ These findings suggest that intradiscal mesenchymal stem cell injection may be considered only for carefully selected patients with confirmed discogenic chronic low back pain after failed conservative management. Patient selection should consider not only concordant discogenic symptoms, but also the biological status of the disc–endplate unit, because advanced endplate sclerosis, calcification, or structural remodeling may impair nutrient diffusion, cell survival, and matrix repair. Given the investigational nature of this therapy, the lack of clear placebo superiority, and unresolved long-term safety and efficacy, intradiscal mesenchymal stem cell injection may be weakly recommended only within ethically approved or registered clinical study settings for carefully selected patients with discogenic chronic low back pain (Level I evidence, Grade C recommendation).

#### Recommendation

For patients with acute low back pain requiring pharmacologic intervention, oral non-steroidal anti-inflammatory drugs (NSAIDs) can be considered as a strong recommendation for superior short-term efficacy.

NSAIDs, including non-selective NSAIDs and selective COX-2 inhibitors, are commonly used for acute low back pain. Their analgesic and anti-inflammatory effects are mainly mediated through cyclooxygenase inhibition and reduced prostaglandin synthesis. Van der Gaag et al. conducted a Cochrane systematic review including 32 randomized controlled trials with 5 356 patients with acute low back pain. Compared with placebo, oral NSAIDs produced greater short-term pain reduction within 3 weeks, with a mean difference of − 7.29 points on a 0–100 visual analogue scale, and greater disability improvement on the Roland-Morris Disability Questionnaire, with a mean difference of − 2.02 points. NSAIDs also showed a small advantage for global improvement. However, these effects did not reach the prespecified minimal clinically important differences, indicating limited clinical relevance. No clear difference in adverse events was found between NSAIDs and placebo, but the short follow-up periods were insufficient to assess rare or long-term harms.^78^ Fukase et al. further conducted a randomized active-controlled trial in 60 patients with low back pain and showed that oral diclofenac was associated with a substantially higher incidence of gastroduodenal ulcers or erosions than a diclofenac systemic patch, highlighting the gastrointestinal risks of oral NSAIDs.^79^ These findings suggest that oral NSAIDs can provide short-term symptomatic benefit in acute low back pain, but their effects are modest and must be balanced against gastrointestinal, renal, and cardiovascular risks. Therefore, oral NSAIDs are strongly recommended for patients with acute low back pain requiring pharmacological treatment, provided they are used at the lowest effective dose for the shortest necessary duration, with individualized risk assessment and gastroprotection or topical/transdermal alternatives considered for patients at increased gastrointestinal risk (Level I evidence, Grade A recommendation).

#### Recommendation

For patients requiring pharmacologic therapy for acute low back pain, the fixed-dose combination of ibuprofen and paracetamol can be considered as a weak recommendation.

The fixed-dose combination of ibuprofen and paracetamol (acetaminophen) is an oral analgesic option that combines two agents with different mechanisms of action. Paracetamol acts mainly through central analgesic pathways, whereas ibuprofen provides peripheral anti-inflammatory and analgesic effects through cyclooxygenase inhibition. Cao et al. conducted a systematic review and meta-analysis including 22 studies evaluating paracetamol plus NSAID combinations for low back pain and osteoarthritis, with one randomized controlled trial specifically involving acute low back pain. In patients with acute low back pain, ibuprofen plus paracetamol produced greater immediate-term pain reduction than ibuprofen alone, with a mean difference of − 6.2 points on a 0–100 scale, and greater disability improvement, with a mean difference of − 9.2 points on a 0–100 scale. The combination did not increase adverse events compared with ibuprofen alone.^80^ However, the evidence for acute low back pain is based on a single trial, and long-term efficacy and safety data are lacking. These findings suggest that the fixed-dose combination of ibuprofen and paracetamol may be considered for short-term symptomatic relief in selected patients with acute low back pain, particularly when response to ibuprofen monotherapy is inadequate. Given the limited evidence base but moderate-quality short-term findings, this combination may be weakly recommended as a pharmacological option for acute low back pain (Level I evidence, Grade B recommendation).

#### Recommendation

Opioids are strongly not recommended for routine management of low back pain; If considered for acute severe pain, use only as a short, low-dose rescue option when NSAIDs are contraindicated or ineffective.

Opioids are potent centrally acting analgesics, but current high-quality evidence does not support their routine use for acute nonspecific low back pain. Jones et al. conducted the OPAL trial, a multicenter, triple-blind, placebo-controlled randomized trial involving 347 patients with acute nonspecific low back or neck pain. Participants received oxycodone–naloxone, up to 20 mg oxycodone daily, or placebo for up to 6 weeks. At 6 weeks, opioids did not significantly improve pain compared with placebo, with mean pain scores of 2.78 versus 2.25, respectively. At 52 weeks, pain scores were worse in the opioid group than in the placebo group, and the risk of opioid misuse was higher with opioids than placebo, at 20% versus 10%. Adverse events were also more frequent in the opioid group, particularly constipation.^81^ Friedman et al. further conducted a randomized trial in emergency department patients with acute low back pain and found that adding oxycodone/acetaminophen to naproxen provided no additional functional benefit over naproxen plus placebo at 1 week, while increasing adverse events.^82^ These findings indicate that opioids do not provide meaningful short- or long-term benefits for acute nonspecific low back pain and may increase the risk of adverse events and later misuse. Therefore, opioids are strongly not recommended for routine management of low back pain; if considered for acute severe pain, they should be used only as a short-term, low-dose rescue option when NSAIDs are contraindicated or ineffective (Level I evidence, Grade A recommendation).

#### Recommendation

For patients with acute non-radicular low back pain, it can be considered as a weak recommendation to routinely use muscle relaxants (such as baclofen, metaxalone, or tizanidine) for a short period of time.

Skeletal muscle relaxants are commonly prescribed for acute low back pain, particularly when painful muscle spasm, marked movement limitation, or sleep disturbance is present. Their role is short-term symptomatic relief rather than modification of the natural course of low back pain. Friedman et al. conducted a randomized, double-blind, placebo-controlled, four-arm trial involving 320 emergency department patients with acute, non-radicular, non-traumatic low back pain of no more than 2 weeks’ duration. All participants received ibuprofen 600 mg as needed and standardized education, and were randomized to additional placebo, baclofen, metaxalone, or tizanidine for 1 week. At 1-week follow-up, improvement in Roland-Morris Disability Questionnaire scores was similar across groups, with mean improvements of 11.1, 10.6, 10.1, and 11.2 points, respectively.^83^ The proportions of patients reporting moderate or severe pain were also comparable among groups. Adverse effects were generally mild and included drowsiness, dizziness, dry mouth, nausea, and headache, with no serious drug-related events. These findings suggest that adding baclofen, metaxalone, or tizanidine to NSAID therapy does not provide clinically meaningful additional benefit for most patients with acute non-radicular low back pain. Given the limited added efficacy and potential sedative effects, skeletal muscle relaxants should not be used routinely or for prolonged periods, but may be considered as a short-course adjunct in carefully selected patients after individualized assessment of sedation, fall risk, driving safety, drug interactions, and central nervous system depression (Level I evidence, Grade B recommendation).

#### Recommendation

For patients with chronic non-specific low back pain, serotonin and norepinephrine reuptake inhibitors (SNRIs, such as duloxetine) can be considered as a weak recommendation.

SNRIs, particularly duloxetine, are centrally acting medications that may reduce pain by enhancing descending inhibitory pain pathways. Ferraro et al. conducted an updated Cochrane systematic review including 26 randomized controlled trials with 2 932 participants to evaluate antidepressants for nonspecific low back pain and spine-related leg pain. For nonspecific low back pain, moderate-certainty evidence showed that SNRIs produced a small short-term reduction in pain compared with placebo, with a mean difference of − 5.25 points on a 0–100 pain scale across 4 trials involving 1 415 participants. However, the effect on disability was trivial, with a mean difference of − 0.91 points on the Roland-Morris Disability Questionnaire across 4 trials involving 1 348 participants. SNRIs also increased the risk of adverse events, with a risk ratio of 1.17 across 5 trials involving 1 510 participants, while evidence for serious adverse events remained very uncertain.^84^ These findings suggest that SNRIs may provide modest short-term analgesic benefit but limited functional improvement in chronic nonspecific low back pain. Therefore, SNRIs should not be used as routine first-line therapy or as a stand-alone substitute for education, exercise, and biopsychosocial management. They may be considered for selected patients, particularly those with persistent pain accompanied by mood symptoms, sleep disturbance, or features of centralized pain, after individualized assessment of contraindications, comorbidities, drug interactions, expected benefits, and tolerability (Level I evidence, Grade B recommendation).

#### Recommendation

Routine use of biologic agents, including anti-TNF-α and anti-NGF therapies, is strongly recommended against for the general management of chronic low back pain, with or without sciatica, because of limited clinical efficacy and an unfavorable or uncertain risk–benefit profile.

Biologic agents, including anti-nerve growth factor (NGF) monoclonal antibodies and anti-tumor necrosis factor-α therapies, have been investigated for chronic low back pain by targeting neuroinflammatory or immune-mediated pain pathways. However, current evidence shows limited clinical efficacy and important safety concerns. Dakin et al. conducted a phase II/III randomized, double-blind, placebo-controlled trial involving 563 patients with moderate-to-severe chronic low back pain who had inadequate response or intolerance to acetaminophen, NSAIDs, and opioids. Patients received different doses of fasinumab or placebo. At week 16, only the 9 mg subcutaneous and 9 mg intravenous fasinumab groups showed statistically significant pain reduction compared with placebo, with least-squares mean differences of − 0.7 points on a 0–10 low back pain intensity scale; the 6 mg group showed no significant benefit. Functional improvement measured by the Roland-Morris Disability Questionnaire was greater than placebo in all fasinumab groups, but the effect sizes were small.^85^ Although overall treatment-emergent adverse events were similar between fasinumab and placebo, joint-related adverse events were more frequent with fasinumab, particularly rapidly progressive osteoarthritis, and occurred mainly in patients with concomitant peripheral osteoarthritis. These findings suggest that anti-NGF therapy provides only modest analgesic and functional benefits in chronic low back pain, with a clinically relevant safety concern in selected patients. Other biologics, including anti-TNF-α agents, have not shown reliable efficacy for nonspecific low back pain. Therefore, routine use of biologic agents is strongly recommended against for the general management of chronic low back pain, with or without sciatica, because of limited clinical efficacy and an unfavorable or uncertain risk–benefit profile (Level I evidence, Grade C recommendation).

#### Recommendation

Preoperative prehabilitation may be weakly recommended as an adjunct optimization strategy for selected patients with low back pain awaiting elective lumbar spine surgery, particularly those at risk of slow recovery

Preoperative prehabilitation is a multimodal intervention delivered before elective lumbar spine surgery, primarily including exercise training, cognitive behavioral therapy (CBT), or pain neuroscience education, aimed at improving postoperative pain, disability, and health-related costs. Oliveira et al. conducted a systematic review and meta-analysis including 8 randomized controlled trials (739 participants). Exercise-based prehabilitation was superior to usual care for disability at 3 months (mean difference − 2.56, 95% CI − 4.98 to − 0.15) and for back pain at 6 months (mean difference − 6.65, 95% CI − 13.25 to − 0.05), and significantly reduced health-related costs at 6 months (mean difference 2 572.8, 95% CI 1 963.0 to 3 182.5). CBT-based prehabilitation was superior to usual care for back pain at 3 months (mean difference − 7.3, 95% CI − 14.5 to − 0.05).^86^ However, all outcomes were supported by very low certainty evidence, primarily limited by heterogeneity, small sample sizes, and risk of bias. Consequently, preoperative prehabilitation may be weakly recommended as an adjunct optimization strategy for selected patients with low back pain awaiting elective lumbar spine surgery, particularly those at risk of slow recovery, but clinical decisions should fully acknowledge the uncertainty of the available evidence, and future high-quality, large-scale, homogeneous randomized controlled trials are warranted (Level I evidence, Grade C recommendation).

Recommendation 5.1.40: Individualized homeopathy is strongly not recommended for routine management of low back pain; consider only within well-designed clinical trials

Individualized homeopathy is an individualized complementary therapy in which homeopathic medicinal products are selected according to patient-specific symptoms and clinical characteristics. A double-blind, randomized, placebo-controlled trial enrolled 60 patients with chronic low back pain and compared individualized homeopathy plus concomitant care with placebo plus concomitant care over 4 months. The individualized homeopathy group showed statistically greater improvement than placebo in Oswestry Disability Index score, with a between-group difference of − 7.8 points. Secondary outcomes also favored individualized homeopathy, including the Short-Form McGill Pain Questionnaire total score and the Roland-Morris Disability Questionnaire score. Adverse events were minor and infrequent, with one case reported in each group.^87^ However, the ODI improvement did not reach the minimal clinically important difference of 12.8 points, and the evidence is limited by the small sample size, short follow-up, and lack of replication. These findings suggest that individualized homeopathy may have statistically detectable effects on pain and disability, but its clinical relevance remains uncertain. Therefore, individualized homeopathy should not be recommended for routine management of chronic low back pain and may only be considered in well-designed clinical studies or, with full disclosure of uncertainty, as a patient-preference-driven adjunct that does not replace evidence-based standard care (Level I evidence, Grade C recommendation).

### Interventional therapy: precise application of minimally invasive techniques and efficacy evaluation

Interventional therapy is primarily applicable to patients with persistent symptoms despite appropriate conservative management, especially when a relatively identifiable pain generator or structural correlate is present. The initiation of this treatment level should be based on concordance between clinical findings and the presumed target structure, together with a clear benefit–risk assessment. Efficacy should be reassessed after short-term follow-up, with attention to pain reduction, functional improvement, duration of effect, and reduction in disability or medication reliance. If repeated interventions fail to provide meaningful or durable benefit, referral for surgical evaluation or broader multidisciplinary reassessment should be considered.

#### Recommendation

Transforaminal epidural steroid injection (TFESI) may be considered as a weak recommendation for selected patients with acute severe sciatica.

Transforaminal epidural steroid injection (TFESI) is an image-guided interventional procedure in which corticosteroid and local anaesthetic are injected around the affected nerve root to reduce nerve-root inflammation and radicular pain. It is mainly considered for selected patients with acute severe sciatica caused by lumbar disc herniation, particularly when pain substantially limits function and responds inadequately to conventional analgesics. Ter Meulen et al. conducted the STAR trial, a randomized controlled trial involving 141 patients with acute sciatica and MRI-confirmed disc herniation with nerve-root impingement. Patients received usual care plus TFESI, usual care plus transforaminal local anaesthetic/saline injection, or usual care alone. Over 6 months, TFESI did not provide clinically relevant additional benefit for most co-primary outcomes, including back pain, physical function, and global perceived recovery. Leg pain was statistically lower with TFESI plus usual care than with usual care alone, but the between-group difference did not reach the minimal clinically important difference. No significant differences were observed between the two injection groups. Post hoc analysis suggested that TFESI increased the proportion of patients achieving at least 50% improvement in leg pain at 3 months and reduced opioid use at 3 and 6 months, with no serious adverse events reported.^88^ A related cost-effectiveness analysis showed no significant improvement in quality-adjusted life years, pain, function, or recovery, and the probability of TFESI being cost-effective remained below 70% across willingness-to-pay thresholds.^89^ These findings suggest that TFESI should not be used routinely for all patients with acute sciatica, but may be considered for carefully selected patients with severe acute radicular pain after inadequate response to conventional care, particularly when short-term leg pain relief or opioid-sparing effects are clinically important. Therefore, TFESI may be weakly recommended for selected patients with acute severe sciatica (Level I/II evidence, Grade B recommendation).

#### Recommendation

For patients with chronic low back pain diagnosed as originating from the lumbar facet joints, radiofrequency (RF) sensory denervation can be considered as a weak recommendation for providing significant and sustained relief of pain and improvement in functional disability.

Radiofrequency denervation is a minimally invasive interventional technique that uses radiofrequency energy to interrupt nociceptive signaling from the medial branch sensory nerves, thereby reducing pain arising from the lumbar facet joints. It is typically considered for patients with suspected facetogenic chronic low back pain who have a positive response to diagnostic medial branch block. Li et al. conducted a systematic review and network meta-analysis including 10 randomized controlled trials with 715 patients with facet joint–derived chronic low back pain. Compared with sham control, conventional radiofrequency denervation produced greater pain relief in both the short term and at 12 months, with moderate-quality evidence. Endoscopic radiofrequency ablation also showed significant short- and long-term pain reduction, while pulsed radiofrequency, pulsed radiofrequency of the dorsal root ganglion, and radiofrequency facet capsule denervation showed benefits at selected time points. Direct comparisons between different radiofrequency techniques generally showed no statistically significant differences.^90^ However, the included trials were limited by small sample sizes, incomplete long-term follow-up, heterogeneous techniques, and limited adverse-event reporting. These findings suggest that radiofrequency denervation, particularly conventional radiofrequency denervation and endoscopic radiofrequency ablation, may provide meaningful pain relief in carefully selected patients with confirmed facet joint–mediated chronic low back pain. Given the moderate-to-fair certainty of evidence, procedure-related risks, and remaining uncertainty regarding long-term durability, radiofrequency denervation may be weakly recommended for selected patients with lumbar facetogenic chronic low back pain after appropriate diagnostic confirmation (Level I evidence, Grade B recommendation).

#### Recommendation

Image-guided sacroiliac joint (SIJ) injection may be considered a weak recommendation for selected patients with confirmed SIJ-mediated low back pain

Image-guided SIJ injection is an interventional technique performed under fluoroscopy, computed tomography (CT) or ultrasound visualization, which delivers local anesthetic combined with corticosteroid into the intra-articular space to relieve pain originating from SIJ lesions. Rajesh et al. completed a systematic review and meta-analysis enrolling a total of 14 qualified studies including 11 RCTs and 3 observational studies, with all eligible trials adopting imaging-guided injection and follow-up duration no less than 3 months¹. Single-arm pooled analysis demonstrated that Numeric Rating Scale (NRS) pain score reduced by approximately 2.979 points and Oswestry Disability Index (ODI) decreased by 18.057 points at 3-month follow-up; at 6 months, the pooled NRS reduction was 3.069 points while functional improvement declined obviously. Among included researches, only 5 RCTs and 2 observational studies achieved positive therapeutic outcomes, and significant heterogeneity existed in injection regimens, diagnostic criteria and research designs across included trials.^91^ These data indicate the treatment obtains limited overall efficacy and inconsistent clinical effects. Based on available Level I evidence, image-guided SIJ injection is only applicable to patients definitely diagnosed with SIJ-mediated low back pain who have failed routine conservative management. Therefore, image-guided SIJ injection may be considered a weak recommendation for selected patients with confirmed SIJ-mediated low back pain (Level I evidence, Grade C recommendation).

#### Recommendation

For patients with acute low back pain related to myofascial trigger points, trigger point injection (TPI) may be considered as a weak recommendation.

TPI is an invasive pain management procedure, which injects 1% lidocaine into confirmed myofascial trigger points to alleviate pain originating from myofascial lesions. Yanuck et al. completed a single-center prospective randomized pragmatic trial, enrolling a total of 52 eligible participants diagnosed with acute myofascial neck and low back pain¹. Subjects were randomly divided into TPI intervention group and conventional medication control group; after 20 min of treatment adjustment for baseline pain, the intergroup median difference of NRS pain score was − 3.01 (95%CI − 4.20 ~ − 1.83, *P* < 0.001). Meanwhile, the median emergency department staying time was obviously shorter in TPI group (2.61 h vs 4.63 h), and the rate of discharge opioid prescription was markedly reduced (2.9% vs 47.4%). Besides, over 88% of patients receiving TPI expressed willingness to receive repeated treatment when similar pain recurs.^92^ These results demonstrate TPI achieves superior immediate analgesic effect and can lower opioid application for myofascial-originated acute low back pain. Based on available Level I evidence, TPI may be selected for acute low back pain patients caused by myofascial trigger points. Therefore, for patients with acute low back pain related to myofascial trigger points, TPI may be considered as a weak recommendation (Level I evidence, Grade B recommendation).

#### Recommendation

Intradiscal glucocorticoid injection (IGI) corticosteroid injection may be considered a weak recommendation for selected patients with chronic discogenic low back pain with active discopathy or modic changes.

IGI is a minimally invasive interventional treatment for discogenic low back pain, delivering glucocorticoids directly into the degenerated intervertebral disc to target local inflammation, particularly indicated for patients with chronic low back pain refractory to conservative management. Nguyen et al. conducted a multicenter randomized controlled trial involving 135 patients with chronic low back pain associated with active discopathy (Modic type 1 changes), demonstrating that a single IGI achieved a significantly higher pain responder rate at 1 month compared with discography alone (55.4% vs 33.3%), although no between-group differences were observed in pain intensity or functional improvement at 12 months.^93^ A 2023 systematic review and meta-analysis of 7 RCTs (*n* = 626) further confirmed that IGI significantly reduced short-term (<3 months) pain scores (mean difference −20.1) and improved physical function (ODI mean difference −9.9), with functional benefits persisting at intermediate-term (3–6 months) but no significant long-term (≥6 months) superiority over control interventions; subgroup analyses indicated that Modic change subtypes did not influence IGI efficacy.^94^ Based on current evidence, IGI may serve as a transitional option for short-term pain relief and functional improvement, though careful patient selection and awareness of its limited long-term durability are warranted (Level I evidence, Grade B recommendation).

#### Recommendation

Image-guided ozone injection may be weakly recommended as an adjunctive option for selected patients with lumbar disc herniation-related radicular low back pain or sciatica after failed conservative care

Image-guided ozone injection is a minimally invasive interventional therapy that injects oxygen-ozone mixed gas into intervertebral disc or periradicular space under fluoroscopy or CT guidance to relieve radicular pain caused by lumbar disc herniation. Llombart-Blanco et al. carried out a systematic review and meta-analysis including nine comparative studies with a total of 1 711 enrolled participants. Pooled analysis showed the standardized mean difference (SMD) of ODI was − 0.28 (95%CI − 0.51 ~ − 0.06) and Visual Analogue Scale (VAS) was − 0.12 (95% CI − 0.24 ~ − 0.01) in overall follow-up; subgroup analysis revealed only 1-month follow-up obtained statistically significant functional improvement, while no obvious intergroup difference was observed at 2 weeks, 3 months and 6 months.^95^ Another relevant meta-analysis including 45 eligible trials further verified that image-guided ozone injection yielded superior pain relief efficacy compared with landmark-guided blind injection, though therapeutic efficacy varied among different age groups and injection dosages.^96^ These pooled data indicated the analgesic benefit of this regimen is transient and inconsistent across long-term follow-up. Based on available Level I/II evidence, image-guided ozone injection can be chosen as supplementary treatment for appropriate patients whose radicular low back pain or sciatica derived from lumbar disc herniation fails conventional conservative management. Therefore, Image-guided ozone injection may be weakly recommended as an adjunctive option for selected patients with lumbar disc herniation-related radicular low back pain or sciatica after failed conservative care (Level I/II evidence, Grade C recommendation).

#### Recommendation

Percutaneous intradiscal radiofrequency thermocoagulation is not recommended as routine care for nonspecific or discogenic low back pain; it may only be considered under research, audit, or strict governance conditions for highly selected patients with chronic discogenic low back pain after failed conservative treatment.

Percutaneous intradiscal radiofrequency thermocoagulation (PIRFT) is an interventional procedure that applies radiofrequency energy to the posterior annulus fibrosus, aiming to reduce chronic discogenic low back pain by modulating or ablating nociceptive fibers within the annulus. Kvarstein et al. conducted a prospective, randomized, double-blind, placebo-controlled trial using the discTRODE™ probe in 20 highly selected patients with single-level positive provocation discography. At the planned 6-month interim analysis, no trend toward benefit was observed for the primary outcome of pain intensity. At 12 months, pain decreased from baseline, but there was no significant difference between active PIRFT and sham treatment, and both groups included patients with increased pain. The authors concluded that intra-annular thermal therapy with the discTRODE™ probe could not be recommended.^97^ Liu et al. later performed a systematic review and meta-analysis including 11 studies, of which 8 were randomized controlled trials, with 374 patients. The pooled analysis suggested that intradiscal radiofrequency procedures may reduce pain and improve Oswestry Disability Index scores at short- and mid-term follow-up, and subgroup analysis suggested that moderate-temperature, short-duration protocols may have a more favorable efficacy and safety profile.^98^ However, the evidence remains limited by heterogeneous techniques, small study sizes, variable comparators, and limited long-term data. Reported adverse events included transient pain, swelling, transient radiculopathy, and one case of discitis, and the procedure does not restore disc structure or clearly prevent recurrence. These findings indicate conflicting evidence, with one high-quality sham-controlled trial showing no superiority over placebo and later pooled analyses suggesting possible benefit in selected settings. Therefore, PIRFT is not recommended as routine care for nonspecific or unselected discogenic low back pain, but may only be considered for highly selected patients with confirmed chronic discogenic pain after failed conservative treatment, preferably under research, audit, or strict governance conditions (Level I evidence, Grade C recommendation).

#### Recommendation

Intradiscal electrothermal therapy may be considered only under research, audit, or strict governance conditions for highly selected patients with chronic discogenic low back pain after failed conservative treatment; routine use for nonspecific low back pain or unselected discogenic pain is not supported.

Intradiscal electrothermal therapy (IDET) is an invasive thermal annular interventional procedure, which delivers conductive heat via intradiscal catheter to denervate inflammatory nociceptive nerve in growth within damaged annulus fibrosus for chronic discogenic low back pain. Helm et al. performed a systematic review including total 4 qualified randomized controlled trials (RCTs) and multiple observational studies. Among the included RCTs, one high-quality trial confirmed obvious pain and functional improvement after IDET, while another moderate-quality controlled study showed no significant therapeutic difference between IDET and sham intervention; pooled observational data revealed inconsistent long-term clinical efficacy across different cohorts.^99^ Comprehensive evidence analysis demonstrated mixed beneficial and negative clinical findings for IDET, lacking stable long-term efficacy data for universal clinical application. Based on available Level I evidence, IDET cannot be applied routinely for ordinary nonspecific low back pain or unselected discogenic patients and is restricted to highly screened sufferers with chronic discogenic low back pain who have exhausted standard conservative treatment and can only be implemented within research, clinical audit or strict standardized management settings. Therefore, Intradiscal electrothermal therapy may be considered only under research, audit, or strict governance conditions for highly selected patients with chronic discogenic low back pain after failed conservative treatment; routine use for nonspecific low back pain or unselected discogenic pain is not supported (Level I evidence, Grade C recommendation).

#### Recommendation

Image-guided plasma disc decompression may be weakly recommended as a minimally invasive option for selected patients with contained lumbar disc herniation-related radicular low back pain or sciatica after failed conservative care, particularly when open surgery is not suitable

Plasma disc decompression (PDD) is a minimally invasive percutaneous technique that uses radiofrequency energy to create a plasma field, thereby reducing intradiscal pressure and decompressing contained disc herniations. It is mainly intended for selected patients with symptomatic contained lumbar disc herniation and persistent radicular pain after failed conservative treatment. Nikoobakht et al. conducted a prospective, single-blinded randomized controlled trial comparing PDD with standard physiotherapy in 177 patients with lumbosacral radicular syndrome and MRI-confirmed contained disc herniation smaller than 6 mm. At 12 months, PDD produced greater reductions in leg pain and visual analogue scale scores than physiotherapy, as well as greater improvements in Oswestry Disability Index and the physical component of the SF-36. Subgroup analyses suggested better outcomes in younger patients, men, patients with lower body mass index, and those with subacute rather than chronic radiculopathy. No major procedure-related complications, including infection or nerve root injury, were reported.^100^ These findings suggest that PDD may provide short- to medium-term benefit for carefully selected patients with contained lumbar disc herniation and radicular pain. However, the evidence is limited by the absence of follow-up beyond 12 months, lack of direct comparison with surgery, and strict selection criteria that limit generalizability. Therefore, image-guided plasma disc decompression may be weakly recommended as a minimally invasive option for selected patients with contained lumbar disc herniation-related radicular low back pain or sciatica after failed conservative care, particularly when open surgery is unsuitable or not preferred after shared decision-making (Level I evidence, Grade C recommendation).

#### Recommendation

Spinal cord stimulation may be weakly recommended as an adjunctive neuromodulation option for selected patients with refractory chronic low back and leg pain, particularly failed back surgery syndrome, persistent spinal pain syndrome, neuropathic or radicular pain, or nonsurgical refractory back pain after failed conservative care and successful trial stimulation

Spinal cord stimulation (SCS), particularly high-frequency 10 kHz SCS, is an implantable neuromodulation technique that delivers paresthesia-free stimulation to the spinal dorsal horn to reduce chronic back and/or leg pain. It is mainly considered for patients with refractory chronic low back and leg pain, including nonsurgical refractory back pain, failed back surgery syndrome, persistent spinal pain syndrome, or neuropathic/radicular pain, after failure of conventional medical management and successful trial stimulation. Kapural et al. conducted a pragmatic, multicenter randomized controlled trial involving 159 patients with nonsurgical refractory back pain, comparing 10 kHz SCS plus conventional medical management with conventional medical management alone. At 3 months, at least 50% pain relief was achieved by 80.9% of patients in the SCS group versus 1.3% in the control group. At 6 months, 78.5% of SCS-treated patients achieved at least 10-point improvement in Oswestry Disability Index, compared with 4% in controls, and mean back pain decreased by 72% in the SCS group while increasing in controls. Quality of life improved and opioid use decreased by 45.8% in the SCS group.^101^ In the 24-month follow-up reported by Patel et al., benefits remained durable, with mean back pain reduced by 73%, an 82% responder rate, a 20-point improvement in ODI, sustained improvement in EQ-5D-5L, and opioid reduction or discontinuation in 62% of baseline opioid users.^102^ Study-related serious adverse events occurred in 3.4% of patients undergoing trial stimulation, and device explantation occurred in 4.8% of implanted patients. These findings suggest that 10 kHz SCS can provide substantial and durable improvements in pain, function, quality of life, and opioid reduction in carefully selected patients with refractory chronic low back and/or leg pain. However, because SCS is invasive, requires successful trial stimulation, and lacks direct comparisons with several other interventional options, it should be used only after careful patient selection and shared decision-making. Therefore, SCS may be weakly recommended as an adjunctive neuromodulation option for selected patients with refractory chronic low back and leg pain after failed conservative care and successful trial stimulation (Level I evidence, Grade A recommendation).

#### Recommendation

Pulsed radiofrequency may be weakly recommended as an adjunctive option for selected patients with refractory chronic lumbar radicular pain or sciatica after failed conservative care or epidural injection

Pulsed radiofrequency is a neuromodulatory technique that applies short pulses of radiofrequency current with longer pauses to avoid permanent tissue damage, targeting the dorsal root ganglion (DRG) to modulate neuropathic pain signals.^103^ A systematic review and meta-analysis of 10 RCTs (*n* = 613) demonstrated that adjuvant PRF of lumbar DRG significantly improved pain scores at 3 months (MD − 1.31, 95% CI − 2.29 to − 0.33) compared to lumbar epidural injection (LEI) alone, with a higher proportion of patients achieving > 50% pain reduction (OR 9.40, 95% CI 2.66 to 33.24).^103^ However, no significant difference was observed in Oswestry Disability Index scores at any time point, and evidence quality ranged from very low to moderate.^103^ Another systematic review of 62 manuscripts (*n* = 3 157) confirmed PRF’s favorable safety profile, reporting zero serious adverse events or persistent neurological deficits, with only 1.14% transient neurological deficits and 3.58% minor adverse events (e.g., bruising, soreness).^104^ Subgroup analyses indicated enhanced efficacy when PRF duration exceeded 360 s, diagnostic blocks were performed, and steroids were not used.^103^Based on the available Level I evidence, its favorable safety profile with zero serious adverse events in over 3 000 patients, sustained analgesic benefits at 3 months, and strong alignment with neuromodulatory mechanisms underlying neuropathic pain, PRF of lumbar DRG is recommended for appropriately selected patients when delivered by trained interventional pain specialists using standardized procedural techniques and imaging guidance. Therefore, PRF is weakly recommended as an adjunctive option for selected patients with refractory chronic lumbar radicular pain or sciatica after failed conservative care or epidural injection (Level I evidence, Grade B recommendation).

#### Recommendation

Dorsal root ganglion stimulation may be weakly recommended as an implantable neuromodulation option for selected patients with refractory neuropathic low back and leg pain, particularly failed back surgery syndrome, persistent spinal pain syndrome, focal lumbosacral radicular pain, or lower-extremity CRPS after failed conservative care and successful trial stimulation

Dorsal root ganglion stimulation (DRGS) is an implantable targeted neuromodulation for refractory neuropathic lumbocrural pain via selective electrical inhibition of abnormal nociceptive conduction. Campos-Fajardo et al. published a systematic review in World Neurosurg in 2024, which included 29 studies (5 RCTs + multiple observational trials, total 987 patients). Baseline VAS averaged 7.8 points and fell to 3.6 points at 1–2 years after valid trial stimulation; analgesic intake reduced by 54.1% on average, with total complication rate 24.7%.^105^ D’Souza et al. completed another systematic review in Adv Ther (2022) containing the landmark ACCURATE RCT plus 39 observational studies and adopting GRADE evaluation. The ACCURATE trial showed DRGS yielded 81.2% and 74.2% pain relief rate at 3 and 12 months versus 55.7% and 53.0% of SCS in CRPS patients; CRPS evidence was downgraded to low Level I, and other pain disorders were supported by low-quality Level II data.^106^ Synthesized evidence indicated obvious heterogeneity across indications, so DRGS is limited to carefully selected patients with successful trial stimulation after failed conservative management rather than routine application. Therefore, Dorsal root ganglion stimulation may be weakly recommended as an implantable neuromodulation option for selected patients with refractory neuropathic low back and leg pain, particularly failed back surgery syndrome, persistent spinal pain syndrome, focal lumbosacral radicular pain, or lower-extremity CRPS after failed conservative care and successful trial stimulation (Level I/II evidence, Grade C recommendation).

#### Recommendation

Peripheral nerve stimulation may be weakly recommended as an adjunctive neuromodulation option for selected patients with refractory chronic low back pain after failed conservative care, particularly failed back surgery syndrome or persistent spinal pain syndrome with predominant axial back pain and successful trial stimulation

Peripheral nerve stimulation, also known as subcutaneous nerve stimulation (SQS), involves implantation of subcutaneous leads in the area of pain to deliver electrical stimulation, representing a less invasive alternative to spinal cord stimulation.^107^ The SubQStim RCT randomized 116 patients with failed back surgery syndrome to SQS plus optimized medical management (OMM) versus OMM alone. At nine months, 33.9% (19/56) of subjects in the SQS + OMM arm achieved ≥ 50% reduction in back pain intensity versus 1.7% (1/60) in the OMM arm (*P* < 0.000 1), with a mean absolute reduction of 33.3 mm on the 100 mm VAS.^108^ A systematic review of 29 studies (828 participants) confirmed consistent modest to moderate improvement in pain intensity with Peripheral Nerve Stimulation.^107^ Based on the available Level Ⅰ evidence, its favorable safety profile with lower complication rates compared to spinal cord stimulation, sustained pain relief benefits, and strong alignment with the neuromodulatory mechanisms underlying refractory axial low back pain, Peripheral Nerve Stimulation is recommended for appropriately selected patients when delivered by trained clinicians using standardized implantation techniques and programming protocols. Therefore, Peripheral Nerve Stimulation is weakly recommended for selected patients with chronic refractory low back pain with prominent failed surgical intervention, predominant axial pain component, successful trial stimulation response, or inadequate relief from conventional neuromodulation requiring salvage therapy (Level I evidence, Grade C recommendation).

### Surgical therapy: selection of indications and pathways for efficacy optimization

Surgical treatment is reserved for patients with definite structural pathology and clear clinical indications, including persistent disabling symptoms despite adequate non-surgical management, concordant imaging and clinical findings, or urgent neurologic or structural conditions requiring operative intervention. The decision to enter this treatment tier should be individualized and made in the context of symptom severity, neurologic status, structural progression, and expected functional gain. Reassessment for surgery should include not only pain persistence, but also neurologic findings, functional limitation, quality-of-life impact, and the adequacy of prior conservative or interventional care. When uncertainty remains, multidisciplinary review and specialist referral are encouraged before definitive surgical decision-making.

#### Recommendation

Image-guided minimally invasive endoscopic discectomy may be considered a weak recommendation for selected patients with lumbar disc herniation-related radicular low back pain

Minimally invasive endoscopic discectomy (ED), including percutaneous transforaminal endoscopic discectomy (PTED) and microendoscopic discectomy (MED), is a surgical technique that removes herniated disc material through a small incision under endoscopic and/or fluoroscopic guidance, with the aim of reducing soft-tissue and bony disruption. It is intended for selected patients with lumbar disc herniation and radicular leg pain who have failed conservative treatment and require surgical decompression. Gadjradj et al. conducted a multicenter non-inferiority randomized controlled trial comparing PTED with open microdiscectomy in patients with sciatica lasting at least 6 weeks. At 12 months, PTED was non-inferior to open microdiscectomy for leg pain reduction, and secondary outcomes including disability, back pain, quality of life, and self-perceived recovery slightly favored PTED. PTED was also associated with less blood loss, more same-day discharge, shorter scars, and lower opioid use at 12 months, while 1-year reoperation rates were comparable between groups. No dural tears or wound infections occurred in the PTED group, whereas these complications were reported in the open microdiscectomy group.^109^ Patel et al. further performed a systematic review and meta-analysis including 14 studies with 1 795 participants and found no significant differences between endoscopic and conventional techniques in back pain, leg pain, Oswestry Disability Index, complication rates, or reoperation rates. ED was associated with a significantly shorter hospital stay.^110^ These findings suggest that ED provides pain relief and functional outcomes comparable to conventional microdiscectomy, with potential advantages in recovery and tissue preservation. However, endoscopic surgery requires appropriate patient selection and is limited by a steep learning curve. Therefore, image-guided minimally invasive endoscopic discectomy may be weakly recommended as an alternative to conventional microdiscectomy for selected patients with lumbar disc herniation-related radicular low back pain after failed conservative care, particularly when performed by experienced surgeons (Level I evidence, Grade B recommendation).

#### Recommendation

Total disc replacement may be considered a weak recommendation for selected patients with chronic discogenic low back pain after failed conservative care

Total disc replacement (TDR) is a motion-preserving surgical procedure that replaces a degenerated intervertebral disc with an artificial prosthesis to relieve pain while maintaining segmental mobility. It is intended for carefully selected patients with symptomatic lumbar degenerative disc disease or discogenic low back pain who have failed adequate conservative treatment. Daniels et al. conducted a meta-analysis including 10 studies with 1 720 patients, comparing lumbar TDR with interbody fusion. No significant differences were found between TDR and fusion in operative time, blood loss, hospital stay, overall complications, reoperation rates, or leg pain. However, TDR produced a statistically significant greater reduction in back pain than fusion, with a mean difference of − 6.95 points.^111^ Wen et al. further performed a systematic review including 22 studies with 2 284 patients and a mean follow-up of 8.3 years. Mean VAS improved from 75.4 to 24.7, and mean ODI improved from 49.8 to 19.4. The mean clinical success and patient satisfaction rates were 74.8% and 86.3%, respectively, with long-term complication and reoperation rates of 18.5% and 13.6%.^112^ These findings suggest that TDR can provide durable pain and functional improvement in selected patients, with outcomes broadly comparable to fusion and a potential theoretical advantage of motion preservation. However, TDR has not shown consistent superiority over fusion across most outcomes, and its success depends strongly on patient selection, implant factors, surgical expertise, and the absence of contraindications such as severe facet arthropathy, osteoporosis, instability, deformity, or multilevel degeneration. Therefore, total disc replacement may be weakly recommended as an alternative to fusion for highly selected patients with chronic discogenic low back pain after failed conservative care, particularly when motion preservation is a clinical priority and contraindications have been excluded (Level I/II evidence, Grade B recommendation).

#### Recommendation

Lumbar spinal decompression may be considered a weak recommendation for selected patients with lumbar spinal stenosis-related neurogenic claudication or sciatica

Lumbar spinal decompression, including laminectomy, laminotomy, and minimally invasive decompression, is a surgical procedure that removes or reshapes compressive bone, ligament, or soft-tissue structures to enlarge the spinal canal and relieve neural compression. It is intended for selected patients with symptomatic lumbar spinal stenosis, with or without degenerative spondylolisthesis, who have neurogenic claudication or radicular leg pain after adequate conservative treatment. Ma et al. conducted a systematic review and meta-analysis including 9 randomized controlled trials with 1 658 patients comparing surgery with non-surgical treatment for lumbar spinal stenosis. No significant difference in Oswestry Disability Index was observed at 6 months, but surgery produced greater functional improvement at 1 year and 2 years. However, surgical treatment was associated with higher overall complication rates, and the evidence did not support one definitive surgical method over others.^113^ For patients with concomitant degenerative spondylolisthesis, the Nordsten-DS randomized non-inferiority trial included 267 patients and compared decompression alone with decompression plus instrumented fusion over 5 years. Decompression alone was non-inferior to decompression with fusion, with 63% of patients in both groups achieving at least 30% ODI improvement, similar ODI changes, and comparable reoperation rates. Decompression alone also had shorter operative time, shorter hospital stay, and lower blood loss.^114^ These findings suggest that lumbar decompression can provide superior functional improvement over conservative treatment after 1–2 years in appropriately selected patients with lumbar spinal stenosis, and that fusion is not routinely required when decompression alone is sufficient. Given the surgical risks, variability in patient selection, and lack of consistent superiority across all endpoints, lumbar spinal decompression may be weakly recommended for selected patients with lumbar spinal stenosis-related neurogenic claudication or radicular pain after failed conservative care and shared decision-making (Level I evidence, Grade B recommendation).

#### Recommendation

Lumbar interbody fusion may be weakly recommended as a surgical stabilization option for selected patients with chronic discogenic low back pain, degenerative spondylolisthesis, spinal instability, recurrent disc herniation with instability, deformity, pseudoarthrosis, or adjacent segment degeneration after failed conservative care, when symptoms and imaging are concordant

Lumbar interbody fusion is a surgical procedure that removes the degenerated intervertebral disc and fuses adjacent vertebrae using interbody cages, bone grafts, and pedicle screw instrumentation. It aims to stabilize or eliminate painful segmental motion, restore disc height, and improve spinal stability. Phillips et al. conducted a systematic review including 26 studies with 3 060 patients undergoing lumbar fusion for chronic low back pain related to degenerative disc disease refractory to non-surgical care. The weighted mean improvement was 36.8 points for back pain on a 0–100 visual analogue scale, 22.2 points for Oswestry Disability Index, and 12.5 points for the SF-36 Physical Component Summary. Patient satisfaction averaged 71.1%, the radiographic fusion rate was 89.1%, and the overall reoperation rate was 12.5%.^115^ However, Austevoll et al. conducted the NORDSTEN-DS randomized trial involving 267 patients with degenerative lumbar spondylolisthesis and spinal stenosis and found that decompression alone was non-inferior to decompression with instrumented fusion at 2 years. At least 30% ODI improvement was achieved by 71.4% of patients in the decompression-alone group and 72.9% in the fusion group, with similar mean ODI improvement. Fusion was associated with longer operative time, greater blood loss, longer hospital stay, and more incidental dural tears.^116^ These findings suggest that lumbar interbody fusion may improve pain and function in carefully selected patients with chronic discogenic low back pain or structural instability after failed conservative care, but it should not be routinely added to decompression when decompression alone is sufficient. Given its higher perioperative burden, resource use, and lack of clear superiority in several degenerative conditions, lumbar interbody fusion may be weakly recommended only for highly selected patients with concordant symptoms and imaging evidence of discogenic pain, degenerative spondylolisthesis with instability, recurrent disc herniation with instability, deformity, pseudoarthrosis, or adjacent segment degeneration after failed conservative treatment and shared decision-making (Level I evidence, Grade B recommendation).

#### Recommendation

Sacroiliac joint fusion may be weakly recommended as a surgical option for selected patients with confirmed sacroiliac joint-mediated low back pain after failed conservative care, particularly degenerative sacroiliitis, sacroiliac joint disruption, or post-lumbar fusion sacroiliac pain; routine use for nonspecific low back pain or unconfirmed sacroiliac pain is not supported.

Minimally invasive sacroiliac joint fusion (MISJF) is a surgical procedure that involves placing triangular titanium implants across the sacroiliac joint via a lateral approach to provide immediate stabilization and promote bony fusion, typically indicated for patients with confirmed sacroiliac joint-mediated pain after failed conservative care. Polly et al. conducted a randomized controlled trial of 148 patients with sacroiliac joint dysfunction comparing MISJF with nonsurgical management. At 6 months, the composite success rate was 81.4% in the MISJF group versus 26.1% in the conservative group; visual analog scale (VAS) pain improved by 52.0 points (from 82.3 to 30.4) in the surgical group compared with 12.2 points (from 82.2 to 70.3) in the conservative group; and the Oswestry Disability Index (ODI) improved by 27.4 points (from 57.2 to 29.9) versus 4.6 points (from 56.0 to 51.6), respectively. A total of 79.5% of conservative-group patients crossed over to surgery after 6 months due to insufficient response, achieving similar improvements in pain and function after crossover, with no significant difference in adverse event rates between groups.^117^ Hermans et al. further performed a systematic review and meta-analysis including 2 randomized controlled trials and one retrospective cohort study with 388 patients, demonstrating that MISJF was associated with a mean reduction of 37.03 points on VAS pain and 21.14 points on ODI compared with conservative management, with zero heterogeneity.^118^ Although the available evidence is subject to some risk of bias due to the unblinded design and industry sponsorship, given the consistent and substantial treatment effects, low adverse event profile, and cost-effectiveness (incremental cost-effectiveness ratio of $13 313 per quality-adjusted life year gained), MISJF may be weakly recommended for selected patients with confirmed sacroiliac joint-mediated low back pain after failed conservative care, particularly those with degenerative sacroiliitis, sacroiliac joint disruption, or post-lumbar fusion sacroiliac pain; routine use for nonspecific low back pain or unconfirmed sacroiliac pain is not supported (Level I evidence, Grade B recommendation).

### Comprehensive intervention: multimodal integration and pathways for personalized management

Comprehensive intervention and long-term management are particularly relevant for patients with chronic, recurrent, or biopsychosocially complex low back pain, especially when single-modality treatment has provided incomplete or non-durable benefit. This treatment level should be considered when long-term functional recovery, recurrence prevention, behavioral modification, and self-management are central goals of care. Efficacy assessment should emphasize sustained pain control, participation, quality of life, self-management ability, and recurrence risk rather than short-term symptom reduction alone. When ongoing impairment persists despite isolated interventions, transition to a coordinated multidisciplinary pathway may be more appropriate than repeated escalation within a single treatment tier.

#### Recommendation

Multidisciplinary biopsychosocial rehabilitation is strongly recommended for selected patients with persistent or chronic low back pain, particularly those with psychosocial barriers, functional disability, poor recovery risk, or inadequate response to previous single-modality treatment; high-intensity multidisciplinary functional restoration programs should be prioritized for complex or disabling cases.

Multidisciplinary biopsychosocial rehabilitation is a comprehensive intervention based on the biopsychosocial model, integrating physical, psychological, and social or occupational components and delivered by professionals from different disciplines. It aims to reduce disability, improve function, and promote social participation by addressing the multiple factors that contribute to persistent low back pain. Kamper et al. conducted a Cochrane systematic review and meta-analysis including 41 randomized controlled trials with 6 858 participants with chronic low back pain. Compared with usual care, moderate-quality evidence showed that multidisciplinary rehabilitation produced modest long-term reductions in pain and disability, equivalent to approximately 0.5 points on a 0–10 pain scale and 1.5 points on the 0–24 Roland-Morris Disability Questionnaire. Compared with physical treatments, multidisciplinary rehabilitation showed greater improvements in pain and disability and increased the likelihood of being at work at 1 year, with an odds ratio of 1.87. Compared with surgery, no significant differences were observed in pain, disability, or work outcomes, while surgery was associated with a higher risk of adverse events.^119^ The 2023 World Health Organization guideline also conditionally recommends multidisciplinary biopsychosocial team care as part of individualized, integrated management for chronic primary low back pain.^120^ These findings suggest that multidisciplinary rehabilitation is particularly suitable for patients with persistent or chronic low back pain who have psychosocial barriers, functional disability, poor recovery risk, or inadequate response to single-modality treatment. Given its favorable safety profile, long-term functional orientation, and ability to address complex biopsychosocial contributors, multidisciplinary biopsychosocial rehabilitation is strongly recommended for selected patients with chronic low back pain, with high-intensity functional restoration programs prioritized for complex or disabling cases (Level I evidence, Grade A recommendation).

#### Recommendation

Guideline-based physiotherapy combined with structured patient education is strongly recommended as a first-line conservative care package for selected patients with persistent, recurrent, or chronic low back pain, particularly those with functional limitation, poor self-management, fear avoidance, or risk of chronicity.

Guideline-based physiotherapy combined with structured patient education integrates evidence-based exercise interventions, manual therapy, and personalized educational sessions. Clinical practice guidelines strongly recommend exercise training including trunk muscle strengthening and specific trunk muscle activation for chronic low back pain.^23^ An RCT of 701 adults showed an individualized walking and education intervention reduced recurrence (HR 0.72, 95% CI 0.60–0.85; *P* = 0.000 2), with median 208 days versus 112 days to recurrence.^121^ A systematic review of 14 trials (*n* = 4 872) demonstrated patient education reduces primary care visits (SMD − 0.14; 95% CI − 0.28 to − 0.00) at 12 months.^122^Based on the available Level I evidence, its favorable safety profile, sustained functional benefits, and strong alignment with the biopsychosocial mechanisms underlying persistent low back pain, guideline-based physiotherapy combined with structured patient education is recommended for appropriately selected patients when delivered by trained clinicians using standardized clinical reasoning and treatment fidelity procedures. Therefore, this intervention is strongly recommended for selected patients with acute, subacute, or chronic nonspecific low back pain with prominent cognitive-behavioral, functional, or lifestyle-related contributors to disability (Level I evidence, Grade A recommendation).

#### Recommendation

Physiotherapist-delivered cognitive-behavioral interventions are strongly recommended as part of guideline-based rehabilitation for selected patients with persistent or chronic low back pain, particularly those with psychosocial barriers, fear avoidance, catastrophizing, low self-efficacy, functional disability, or high risk of poor recovery.

Physiotherapist-delivered cognitive-behavioral interventions integrate structured exercise therapy with cognitive-behavioral strategies to improve physical function, pain-related beliefs, and behavioral responses to pain. They are delivered by trained physiotherapists and typically include pain education, cognitive restructuring, goal setting, activity pacing, graded exposure, relaxation techniques, and home-based self-management tasks. Ho et al. conducted a systematic review and network meta-analysis including 97 randomized controlled trials with 13 136 participants with chronic nonspecific low back pain. Compared with physiotherapy care alone, cognitive-behavioral therapy combined with physiotherapy produced large improvements in physical function and pain intensity at post-intervention, with standardized mean differences of 1.01 and 0.92, respectively. It also produced a large improvement in fear avoidance, with an SMD of 1.77. Benefits for pain were sustained to mid-term follow-up, while functional benefits were sustained to short-term follow-up.^123^ Hall et al. further reviewed 5 randomized controlled trials with 1 390 participants in primary care outpatient settings and found that physiotherapist-delivered cognitive-behavioral interventions produced small but significant long-term reductions in disability and pain compared with other guideline-based active treatments. All included studies required additional physiotherapist training and used treatment manuals to support intervention fidelity.^124^ These findings suggest that physiotherapist-delivered cognitive-behavioral interventions are particularly suitable for patients with persistent or chronic low back pain who have psychosocial barriers, fear avoidance, catastrophizing, low self-efficacy, functional disability, or high risk of poor recovery. Given the Level I evidence, favorable safety profile, and alignment with guideline-based biopsychosocial rehabilitation, these interventions are strongly recommended when delivered by trained physiotherapists as part of structured rehabilitation (Level I evidence, Grade A recommendation).

#### Recommendation

Group-based exercise and education intervention is strongly recommended as a scalable first-line conservative care option for selected patients with persistent, recurrent, or chronic low back pain, particularly those who need improved self-management, physical activity, exercise confidence, and functional recovery

Group-based exercise and education is a structured physiotherapist-delivered intervention that combines supervised group exercise with education on pain understanding, activity management, self-efficacy, and self-management. A multicenter randomized controlled trial comparing cognitive functional therapy (CFT) with group-based exercise and education included 100 participants allocated to the group intervention, which consisted of up to 6 classes over 6–8 weeks. Both interventions improved disability and pain over follow-up; however, CFT produced greater reductions in disability than group-based exercise and education at 6 months and 12 months, with mean differences of 8.65 and 7.02 points, respectively.^125^ Clinical guidelines support exercise therapy as a core component of low back pain management, and group-based delivery may offer a scalable and potentially cost-effective format for selected patients.^126^ These findings suggest that group-based exercise and education is suitable for patients who require improved self-management, exercise confidence, physical activity, and functional recovery, particularly when peer-supported rehabilitation or lower-resource delivery is preferred. Given its favorable safety profile, practical scalability, and consistency with guideline-based active rehabilitation, group-based exercise and education is strongly recommended as a first-line conservative care option for selected patients with persistent, recurrent, or chronic low back pain (Level I evidence, Grade A recommendation).

#### Recommendation

Risk stratification combined with shared decision-making may be weakly recommended as a patient-centered clinical pathway for selected patients with low back pain

Risk stratification combined with shared decision-making is a patient-centered clinical pathway that uses validated prognostic tools, such as the STarT Back tool, to classify patients with low back pain into low-, medium-, or high-risk groups and match them with different treatment intensities, while incorporating patient preferences through structured decision aids. Hill et al. conducted the STarT Back randomized controlled trial involving 1 573 patients with low back pain. At 12 months, stratified care produced greater improvement in Roland-Morris Disability Questionnaire scores than usual care, with an adjusted mean difference of 1.06 points, and was associated with a mean healthcare cost saving of £34.39 and an additional 0.039 quality-adjusted life years.^127^ Stacey et al. further conducted a Cochrane systematic review of patient decision aids including 209 studies with 107 698 participants. Compared with usual care, decision aids improved patient knowledge, increased the accuracy of risk perception, improved concordance between informed choices and patient values, and did not increase decision regret.^128^ These findings suggest that risk stratification can improve care efficiency and outcomes in low back pain, while shared decision-making may support informed and preference-sensitive treatment choices. However, evidence for the combined pathway is partly indirect, and implementation requires validated tools, clinician training, and sufficient consultation time. Therefore, risk stratification combined with shared decision-making may be weakly recommended as a patient-centered management pathway for selected patients with low back pain, particularly those with clear prognostic risk, recurrent symptoms, complex treatment choices, or decisional uncertainty (Level I evidence, Grade B recommendation).

#### Recommendation

Risk-stratified tailored care may be weakly recommended as a clinical pathway for selected patients with low back pain, using validated risk tools to match low-risk patients to reassurance and self-management, medium-risk patients to guideline-based physiotherapy, and high-risk patients to psychologically informed or more intensive support.

Risk-stratified tailored care is a primary care management model that uses prognostic screening tools, such as the Keele STarT Back Screening Tool, to classify patients with low back pain into low-, medium-, and high-risk groups and match them with treatment pathways of different intensity. It aims to provide more efficient and proportionate care according to each patient’s risk of poor outcome. Hill et al. conducted the STarT Back randomized controlled trial involving 851 patients with low back pain from 10 general practices in England. At 12 months, stratified care produced greater improvement in Roland-Morris Disability Questionnaire scores than non-stratified best practice, with an adjusted between-group difference of 1.06 points; the benefit was also significant at 4 months, with a difference of 1.81 points. Medium-risk patients showed greater improvement at 12 months, and high-risk patients showed greater improvement at 4 months. Low-risk patients achieved non-inferior outcomes while receiving substantially less treatment, with only 7% referred for further physiotherapy compared with 49% of low-risk controls. Stratified care also reduced pain catastrophizing, fear avoidance, anxiety, depression, and work absence, while generating 0.039 additional quality-adjusted life years and a mean healthcare cost saving of £34.39.^127^ Whitehurst et al. further confirmed that stratified care was cost-effective across risk subgroups, with a probability of cost-effectiveness exceeding 95% for medium- and high-risk patients at a willingness-to-pay threshold of £20 000 per quality-adjusted life year.^129^ These findings suggest that risk-stratified tailored care can improve outcomes, reduce unnecessary treatment, and optimize healthcare resource use. However, implementation requires systematic screening, pathway matching, clinician training, and service capacity. Therefore, risk-stratified tailored care may be weakly recommended for patients with low back pain when primary care systems can support structured risk assessment and matched treatment pathways (Level I evidence, Grade B recommendation).

#### Recommendation

Back school may be weakly recommended as an adjunctive group-based education and exercise option for selected patients with chronic or recurrent non-specific low back pain, particularly in occupational settings were ergonomic education, self-management, graded activity

Back school is a structured education- and exercise-based intervention that combines information on spinal anatomy, pain mechanisms, posture, ergonomics, lifting techniques, self-management, and supervised therapeutic exercise. It is commonly delivered in a group format and is used for patients with chronic or recurrent nonspecific low back pain, particularly in occupational and rehabilitation settings. Straube et al. conducted a systematic review and meta-analysis including 31 randomized controlled trials evaluating back school interventions for chronic low back pain. Compared with no intervention, back school showed short-term improvements in pain and disability in some pooled analyses, but effects were inconsistent at longer follow-up, and comparisons with other active treatments were limited by substantial clinical and methodological heterogeneity.^130^ Hernandez-Lucas et al. further performed a systematic review and meta-analysis including 25 randomized controlled trials with 4 454 participants with nonspecific back pain and found that back school programs significantly reduced pain and disability with moderate-certainty evidence, although heterogeneity and possible publication bias were noted.^131^ These findings suggest that back school may provide modest benefits, particularly when combined with exercise, ergonomic education, and active self-management. Given the variability in program content, limited evidence of superiority over other active interventions, and uncertain long-term effects, back school should be considered an adjunctive component of a broader rehabilitation strategy rather than a stand-alone treatment. Therefore, back school may be weakly recommended for selected patients with chronic or recurrent nonspecific low back pain, especially when education, ergonomic modification, and graded activity can be integrated into a biopsychosocial care plan (Level I evidence, Grade B recommendation).

#### Recommendation

Digital support interventions for the self-management may be weakly recommended as an adjunctive delivery mode for selected patients with persistent, recurrent, or chronic non-specific low back pain, particularly those who need structured education, home exercise support, activity monitoring, adherence reminders, and flare-up self-management

Digital support interventions for self-management of low back pain are interactive, technology-based programs delivered via web or mobile platforms to support patients in self-managing their condition through education, symptom monitoring, and activity promotion. A systematic review of 6 completed RCTs involving 2 706 participants found that digital self-management interventions varied considerably in their nature and delivery, with only one study showing between-group differences favoring the digital intervention.^132^ A recent RCT of 2016 participants found that adding an internet intervention (SupportBack) to usual care did not significantly reduce low back pain–related disability at 12 months compared with usual care alone (adjusted mean difference 0.40, 95% CI − 0.15 to 0.96).^133^ No studies showed evidence of harm. The current evidence base for digital self-management interventions remains weak, with substantial heterogeneity limiting conclusions about optimal components or target populations. Based on the available Level I evidence, its favorable safety profile with no evidence of harm, convenience for patients, and potential scalability to increase access to self-management support, digital support interventions are conditionally recommended for appropriately selected patients when delivered through well-designed platforms with appropriate clinical oversight. Therefore, digital support interventions for self-management are weakly recommended for selected patients with low back pain who are comfortable with technology use, particularly those with limited access to face-to-face care (Level I evidence, Grade B recommendation).

#### Recommendation

Stratified blended physiotherapy may be weakly recommended as an adjunctive delivery model for selected patients with non-specific low back pain, particularly those who need risk-stratified support, structured education, home exercise guidance, activity monitoring, and improved self-management between face-to-face sessions.

Stratified blended physiotherapy is a blended care model that combines face-to-face physiotherapy with app or internet based self-management support, with treatment intensity tailored according to prognostic risk using tools such as the Keele STarT Back Screening Tool. It aims to improve self-management, exercise adherence, and continuity of care between clinical sessions through video-supported education, home exercise guidance, and activity-based modules. Koppenaal et al. conducted the e-Exercise LBP cluster randomized controlled trial involving 208 patients with nonspecific low back pain from 58 primary care physiotherapy practices. At 3 months, stratified blended physiotherapy did not significantly improve physical function compared with face-to-face physiotherapy alone, although both groups achieved clinically relevant within-group improvements. However, the blended approach produced greater reductions in fear-avoidance beliefs and better adherence to prescribed home exercises. In the high-risk subgroup, it showed greater improvements in physical function, pain intensity, and fear-avoidance beliefs.^134^ Geraghty et al. further conducted the SupportBack 2 randomized trial involving 825 UK primary care participants and found that internet-based self-management, with or without physiotherapist telephone support, did not significantly reduce low back pain-related disability compared with usual care over 12 months. However, economic evaluation suggested that digital self-management support was likely to be cost-effective, with no treatment-related serious adverse events.^135^ These findings suggest that stratified blended physiotherapy may not be superior to standard face-to-face physiotherapy for overall functional improvement in unselected patients, but may improve adherence, fear avoidance, and outcomes in higher-risk patients. Therefore, stratified blended physiotherapy may be weakly recommended as an adjunctive delivery model for selected patients who require risk-stratified support, structured education, home exercise guidance, and enhanced self-management between face-to-face sessions (Level I evidence, Grade B recommendation).

#### Recommendation

Workplace interventions may be weakly recommended as an adjunctive occupational management strategy for selected workers with occupational low back pain, recurrent work-related symptoms, reduced work ability, or sickness absence risk, particularly when workplace modification, temporary work accommodation, supervisor engagement, and return-to-work coordination are integrated with guideline-based clinical care

Workplace interventions for low back pain are multi-domain programs delivered in the work setting to reduce disability, support work participation, and facilitate return to work. They typically combine health-focused care, service coordination, and work modification, such as ergonomic adjustment, graded activity, communication with employers, and modified duties. A systematic review and meta-analysis including 14 studies found that workplace interventions significantly reduced pain, disability, and fear-avoidance related to physical activity, and improved both physical and mental components of quality of life compared with control interventions. They also significantly reduced low back pain recurrence, although reductions in the number of participants on sick leave and total sick-leave days did not reach statistical significance.^136^ Another systematic review found strong evidence that multi-domain interventions including at least two of three components—health-focused care, service coordination, and work modification—reduced time away from work for musculoskeletal or pain-related conditions.^137^ These findings suggest that workplace interventions are particularly suitable for workers with work-related low back pain, prolonged disability, fear-avoidance behaviors, ergonomic risk factors, or difficulty returning to work. Given the Level I evidence, favorable safety profile, and potential benefits for function, recurrence prevention, and work participation, workplace interventions may be weakly recommended for selected workers with low back pain when delivered through coordinated healthcare and workplace support (Level I evidence, Grade B recommendation).

#### Recommendation

Digital therapeutics using artificial intelligence-assisted telerehabilitation may be weakly recommended as an adjunctive delivery model for selected patients with persistent, recurrent, or chronic non-specific low back pain, particularly those who need home-based exercise guidance, real-time movement feedback, adherence support, and remote self-management

Artificial intelligence-assisted telerehabilitation for chronic nonspecific low back pain usually combines digital rehabilitation with either AI-based real-time motion tracking and corrective feedback or app-based personalized self-management recommendations. Xiao et al. conducted a randomized controlled trial involving 38 patients with chronic nonspecific low back pain, comparing AI-assisted multimodal exercise telerehabilitation with conventional video-guided exercise. At 4 weeks, the AI-assisted group showed greater reduction in worst pain intensity than the control group, with an adjusted mean difference of − 1.08 points on the Numerical Rating Scale, and the improvement reached the minimal clinically important difference. The effect was sustained at 8 weeks.^138^ In contrast, Marcuzzi et al. conducted a three-arm randomized clinical trial involving 294 patients with neck and/or low back pain and evaluated an AI-based self-management app that provided tailored recommendations without real-time movement correction. At 3 months, the app did not significantly improve musculoskeletal health compared with usual care or a non-tailored web-based intervention.^139^ These findings suggest that AI-assisted telerehabilitation may be more effective when it includes real-time movement detection and corrective feedback rather than passive app-based recommendations alone. However, current evidence is limited by small sample sizes, heterogeneous digital platforms, short follow-up, and inconsistent clinical effects. Therefore, AI-assisted telerehabilitation may be weakly recommended as an adjunctive delivery model for selected patients with persistent nonspecific low back pain, particularly those with mild to moderate disability who require home-based exercise guidance, adherence support, and real-time movement feedback, while patients should be informed of the current evidence limitations (Level I evidence, Grade B recommendation).

#### Recommendation

Wearable devices plus usual physiotherapy care may be weakly recommended as an adjunctive activity-monitoring and behavior-change tool for selected patients with non-specific low back pain at medium or high risk of chronicity, particularly those with physical inactivity, poor exercise adherence, or need for step-count feedback and walking progression.

Wearable devices, such as Fitbit devices and smartwatches, are commercially available activity-monitoring tools that support physical activity behavior change through self-monitoring, goal setting, and real-time feedback. When combined with physiotherapy or self-management programs, they can be used to monitor step counts, activity intensity, and walking progression between clinical sessions. Ebereime et al. conducted a systematic review including 13 studies, of which 3 randomized controlled trials involved low back pain populations. In patients with low back pain, wearable devices combined with usual physiotherapy care increased daily step counts by approximately 1 148–2 649 steps per day and improved time spent in light and moderate-to-vigorous physical activity compared with physiotherapy alone. However, effects on pain intensity were inconsistent, and most between-group differences were not statistically significant. No serious adverse events were reported, with only mild muscle soreness or skin irritation described.^140^ Ferguson et al. further conducted an umbrella review including 39 systematic reviews and 163 992 participants, showing that wearable activity trackers increased physical activity, equivalent to approximately 1 800 additional steps per day and 40 extra minutes of walking per day. Effects on pain, quality of life, and disability were generally small and often non-significant.^141^ These findings suggest that wearable devices are more useful for improving activity monitoring, exercise adherence, and behavioral engagement than for direct pain relief. Therefore, wearable devices may be weakly recommended as an adjunctive activity-monitoring and behavior-change tool for selected patients with nonspecific low back pain, particularly those with physical inactivity, poor exercise adherence, or a need for step-count feedback and walking progression (Level I evidence, Grade C recommendation).

## Future research directions

Studies over the past few decades have shown that the pathogenic factors of LBP are complex and diverse. While lumbar structures such as intervertebral discs (IVDs) and facet joints are considered plausible sources of non-specific LBP, pain cannot be reliably attributed to these anatomical structures solely through clinical examinations.^142,143^ As demonstrated in relevant studies published in The Lancet, IVD degeneration is also frequently observed in asymptomatic individuals, indicating that degeneration itself does not necessarily induce pain.^144,145^ This highlights the significant limitations of the traditional diagnostic and therapeutic approach that solely focuses on anatomical abnormalities.

Notably, the association between LBP and IVD is not absolute. Following ossification of the vertebral endplates (EPs), osteoclast-mediated resorption of calcified endplates forms porous endplates during spinal degeneration. This uncoupled remodeling process increases endplate volume, which significantly occupies the intervertebral disc (IVD) space and ultimately contributes to disc degeneration.^146,147^ This cascade suggests that intervertebral disc degeneration may be a consequence of porous endplate expansion rather than the primary cause of spinal degeneration and LBP. This clarification is also supported by clinical observations indicating that there is no direct association between disc degeneration and low back pain.^148^ Further evidence indicates that the origin of LBP may be more closely related to cartilaginous endplate–associated lesions. Therefore, future therapies should consider the IVD as an integrated functional unit, with particular attention to the endplate, which may be crucial for improving the diagnosis and treatment outcomes of LBP.^149^

Meanwhile, the clinical management of LBP is gradually shifting from a structure-centered, single-modality approach to an evidence-based, comprehensive framework oriented toward functional recovery and participation in daily life. With the rapid accumulation of evidence from randomized controlled trials, systematic reviews, and real-world studies, both international consensus statements and routine practice increasingly emphasize that LBP care should be grounded in precise assessment and risk stratification, delivered through stepwise, individualized, and multidisciplinary pathways, and guided by an integrated decision framework that balances expected benefits, potential harms, costs, feasibility of implementation, and patient preferences.

Based on a systematic appraisal of current evidence and rigorous consensus procedures, this consensus proposes a graded, recommendation-based care model. At the same time, it highlights persistent challenges in the field, including substantial heterogeneity of evidence, limited applicability across diverse healthcare settings, unclear implementation pathways, and underdeveloped mechanisms for incorporating patient preferences. To advance a care system that is implementable, evaluable, and continuously updatable, the expert panel reached consensus on future research priorities across six key domains:

1. Develop context-specific and actionable care pathways. For primary care, rehabilitation services, and pain centers, recommendation-driven digital clinical decision support tools should be developed and validated to operationalize a standardized workflow—from phenotyping and risk stratification to stepwise interventions and follow-up with relapse prevention. Core outcomes should include functional recovery, participation, recurrence risk, and efficiency of healthcare resource utilization, aligning with truly patient-centered goals.
2. Establish a dynamic evidence-updating infrastructure. Leveraging electronic health records, disease registries, digital therapeutics, and wearable monitoring, an evidence-updating platform integrating real-world data and artificial intelligence should be built to continuously evaluate long-term effectiveness and safety of interventions, identify treatment-effect heterogeneity across clinical subgroups, and provide timely evidence for periodic updates of the consensus.
3. Strengthen mechanisms for incorporating patient preferences through shared decision-making. Shared decision-making tools and patient decision aids should be systematically developed, alongside an operational workflow for eliciting and integrating patient values and preferences. The impact of these mechanisms on adherence, satisfaction, self-management capacity, and long-term functional outcomes should be quantitatively evaluated to better align clinical decisions with both evidence and patient priorities.
4. Refine clinical phenotypes and advance stratified treatment research. To address the current overemphasis on imaging-defined degeneration, prospective studies should validate a more granular LBP phenotyping system that integrates pathophysiological mechanisms, psychosocial factors, and functional status. Stratified trials are needed to test the net benefit of mind–body and function-focused pathways in specific subtypes.
5. Strengthen weak evidence areas and incorporate health economic evaluations. For interventions graded as “weakly recommended” or “weakly non recommended,” enrichment designs and adaptive trials should be employed to generate high-quality evidence in well-defined subgroups. Cost-effectiveness and cost-utility analyses should be conducted in parallel to inform resource allocation and reduce low-value care.
6. Promote implementation and translation research in primary care settings. Hybrid effectiveness–implementation randomized trials should be encouraged in community and primary care contexts, such as a 12-month individualized exercise prescription as a secondary prevention program, with concurrent evaluation of real-world effectiveness, implementation feasibility, barriers and facilitators, and cost implications—moving LBP care toward value-based healthcare with function as the central outcome.

## Conclusion

This multidisciplinary expert consensus on clinical care models and recommendation levels for low back pain (LBP), grounded in a systematic integration of current evidence and clinical experience, proposes a comprehensive framework spanning etiology identification and phenotype-based assessment, risk stratification, stepped interventions, and long-term management with recurrence prevention, and clearly delineates key clinical decision points through a structured grading of recommendation strength. It not only provides clinicians and rehabilitation-related professionals with an actionable pathway reference, but also offers researchers and healthcare administrators a relatively unified evidence framework and practical boundaries, thereby helping to move LBP management from “fragmented, discipline-specific measures” toward “integrated care centered on function and participation in daily life.” We recommend that future research and translational implementation efforts adhere, as much as possible, to the phenotyping/stratification logic and recommendation grading system proposed in this consensus, and further validate its feasibility and real-world benefits across different healthcare settings (e.g., primary care clinics, specialty centers, and rehabilitation or pain-management platforms). Importantly, the stepped-care approach advocated by this consensus is not intended to place different treatment modalities in competition. Rather, it emphasizes multimodal conservative management as the foundation, and—under clearly defined indications and patient-centered goals—rational integration of necessary interventional and surgical strategies, while embedding education, self-management, and recurrence prevention throughout the entire care continuum to achieve better treatment matching and improved long-term functional outcomes. Finally, although a unified set of recommendations has been established, broader dissemination and implementation will require continued refinement through higher-quality research. Future work should prioritize strengthening evidence on the effectiveness and safety of “weakly recommended” or “weakly not recommended” interventions in specific subgroups, and focus on removing implementation barriers—particularly those related to primary care adoption, cross-disciplinary collaboration mechanisms, digital decision-support tools, costs and resource allocation, and public health promotion. Policymakers and health system leaders may consider incorporating risk stratification and value-oriented stepped care into regional or national chronic disease and musculoskeletal health strategies, while concurrently developing usable tools, supportive environments, and training systems, to facilitate a steady transition from “expert consensus” to “replicable and sustainable routine clinical practice” in LBP care.

## Supplementary information


Supplementary information

