# Supplementary material for: Multidisciplinary expert consensus on clinical diagnosis and therapies for low back pain
Source: Bone Res. 2026 Aug 27;14:92. doi: 10.1038/s41413-026-00572-y (PMC13522480; doi:10.1038/s41413-026-00572-y)
Supplement: Supplementary file 1 [file 41413_2026_572_MOESM1_ESM.docx]

**SUPPLEMENTARY FILE 1 (SEARCH STRATEGY)**

| **Multidisciplinary expert consensus on clinical diagnosis and therapies for low back pain** | | |
| --- | --- | --- |
| **Database** | **Term** | **Results** |
| PubMed | (("Low Back Pain"[Majr]) AND ("Therapeutics"[Majr])) OR (((Therapy[Title/Abstract]) OR (Treatment[Title/Abstract])) AND (((((((((((Lumbago[Title/Abstract]) OR (Low Back Ache[Title/Abstract])) OR (Low Back Pain, Mechanical[Title/Abstract])) OR (Low Back Pain, Posterior Compartment[Title/Abstract])) OR (Low Back Pain, Postural[Title/Abstract])) OR (Low Back Pain, Recurrent[Title/Abstract])) OR (Low Backache[Title/Abstract])) OR (Lower Back Pain[Title/Abstract])) OR (Mechanical Low Back Pain[Title/Abstract])) OR (Postural Low Back Pain[Title/Abstract])) OR (Recurrent Low Back Pain[Title/Abstract]))) | **9106** |
